# *miR2118*-triggered phased siRNAs are differentially expressed during the panicle development of wild and domesticated African rice species

**DOI:** 10.1186/s12284-016-0082-9

**Published:** 2016-03-12

**Authors:** K. N. Ta, F. Sabot, H. Adam, Y. Vigouroux, S. De Mita, A. Ghesquière, N. V. Do, P. Gantet, S. Jouannic

**Affiliations:** IRD, UMR DIADE, 911, avenue Agropolis, BP64501, F-34394 Montpellier, Cedex 5 France; LMI RICE, National Key Laboratory for Plant Cell Biotechnology, Agronomical Genetics Institute, Pham Van Dong road, Hanoi, Vietnam; Université de Montpellier, UMR DIADE, Place Eugène Bataillon, F-34095 Montpellier, Cedex 5 France; Present address: INRA, Université de Lorraine, UMR 1136 Interactions Arbres/Microorganismes, F-54280 Champenoux, France

**Keywords:** Panicle, Meristem, Small RNAs, phasiRNAs, *miR2118*, Domestication, *Oryza barthii*, *Oryza glaberrima*

## Abstract

**Background:**

Rice exhibits a wide range of panicle structures. To explain these variations, much emphasis has been placed on changes in transcriptional regulation, but no large-scale study has yet reported on changes in small RNA regulation in the various rice species. To evaluate this aspect, we performed deep sequencing and expression profiling of small RNAs from two closely related species with contrasting panicle development: the cultivated African rice *Oryza glaberrima* and its wild relative *Oryza barthii*.

**Results:**

Our RNA-seq analysis revealed a dramatic difference between the two species in the 21 nucleotide small RNA population, corresponding mainly to *miR2118*-triggered phased siRNAs. A detailed expression profiling during the panicle development of *O. glaberrima* and *O. barthii* using qRT-PCRs and *in situ* hybridization, confirmed a delayed expression of the phased siRNAs as well as their lncRNA precursors and regulators (*miR2118* and *MEL1* gene) in *O. glaberrima* compared to *O. barthii*. We provide evidence that the 21-nt phasiRNA pathway in rice is associated with male-gametogenesis but is initiated in spikelet meristems.

**Conclusion:**

Differential expression of the *miR2118*-triggered 21-nt phasiRNA pathway between the two African rice species reflects differential rates of determinate fate acquisition of panicle meristems between the two species.

**Electronic supplementary material:**

The online version of this article (doi:10.1186/s12284-016-0082-9) contains supplementary material, which is available to authorized users.

## Background

A prevailing view in evolutionary developmental biology (Evo-Devo) is that morphological traits evolved mostly by changes in expression patterns of functionally conserved genes rather than through the emergence of new genes (Doebley and Lukens 1998; Carroll 2008). Although much emphasis was placed on changes in transcriptional regulation, gene expression is regulated at many levels. In this context, the regulation of genome expression by small RNAs appears to be an important mechanism in the control of plant development (morphogenesis and phase transition), through post-transcriptional regulation of mRNA abundance (by *miRNAs* and secondary *siRNAs*) and silencing of gene expression (by *siRNAs*) *via* site-specific DNA methylation (Jones-Rhoades et al. 2006; Arikit et al. 2013). Secondary, phased small interfering RNAs (phasiRNAs) are emerging regulators of gene expression in plants (Zheng et al. 2015; Xia et al. 2013). Analyses in large panel of plant species covering plant kingdom revealed that a huge diversity of phasiRNA-generating loci types in plants (Zheng et al. 2015; Xia et al. 2013). An unsuspected diversity of phasiRNAs originating from protein-coding genes was observed. However, most of these loci were clade- or species-specific with distinct expression patterns, suggesting recent and independent evolutionary origins (Xia et al. 2013).

Several types of phasiRNA-generating loci were reported from Asian rice *Oryza sativa* and its wild relative *Oryza rufipogon* from various tissues or organs (Arikit et al. 2013; Zheng et al. 2015; Liu et al. 2013). In *O. sativa*, phasiRNAs triggered by specific miRNAs and originating from non-coding loci, were shown to be involved in panicle development and more specifically in male gametogenesis (Arikit et al. 2013; Johnson et al. 2009; Komiya et al. 2014; Song et al. 2012a, b). The *TAS3*-associated trans-acting siRNAs (or ta-siRNAs), triggered by the microRNA *miR390*, are known to target mRNAs of *Auxin Response Factor* (*ARFs*) involved in various developmental processes including floret and stamen development (Allen et al. 2005; Liu et al. 2007; Nogueira et al. 2007; Song et al. 2012b). Other detected phasiRNAs are panicle-specific 21- or 24-nucleotide (nt) small RNAs. They are produced from numerous polyA-tailed long non-coding RNA (lncRNA) generating loci through an RDR6-dependent pathway triggered by the microRNAs *miR2118* and *miR2275* respectively (Komiya et al. 2014; Song et al. 2012a, b). The 21-nt phasiRNAs may act in a complex with the gamete-specific Argonaute (AGO) protein MEIOSIS ARRESTED AT LEPTOTENE1 (MEL1) (Komiya et al. 2014). In maize, each phasiRNA type exhibits independent spatiotemporal regulation, with 21-nt premeiotic phasiRNAs dependent on stamen epidermal differentiation and 24-nt meiotic phasiRNAs dependent on tapetal cell differentiation (Zhai et al. 2015). However, their function during male gametogenesis remains unclear.

African rice *Oryza glaberrima* was domesticated about 2000 to 3000 years ago from *Oryza barthii*, whereas *O. sativa* was domesticated in Asia almost 10,000 years ago from *Oryza rufipogon* (Second 1982; Linares 2002; Caicedo et al. 2007; Vaughan et al. 2008; Huang et al. 2012; Orjuela et al. 2014; Wang et al. 2014; Zhang et al. 2014a. African rice domestication has a single origin in West Africa (Linares, 2002; Wang et al. 2014) and is associated with a severe genetic bottleneck (Li et al. 2011a, b; Nabholz et al. 2014; Orjuela et al. 2014). It is also associated with major morphological and physiological changes. For example, *O. barthii* and *O. glaberrima* exhibit strikingly different panicle architectures, from a low branching complexity and small grain number in *O. barthii* to a more complex panicle and higher grain number in *O. glaberrima* (Linares 2002). A similar divergence of domestication-related traits was also observed between the Asian wild rice and its cultivated counterpart (Vaughan et al. 2008). It has been suggested that several small RNA loci, such as miRNA-triggered phasiRNAs loci including ta-siRNA locus *TAS3a2,* as well as the microRNA loci *MIR164e*, *MIR390* and *MIR395a/b,* have experienced direct selection during Asian rice domestication (Liu et al. 2013; Wang et al. 2010; Wang et al. 2012). However, a large-scale study of small RNA expression in relation to the early stages of panicle development in African rice is still lacking. To investigate whether the latter varies between the two African species in relation to changes in small RNA expression, we conducted a comparative analysis of small RNA populations accumulating during early panicle development in the two African species*.* We found conservation of the male gametogenesis-associated *miR2118*-triggered phasiRNA pathway in these African rice species. Our data revealed significant changes in expression of these gamete-specific 21-nt phasiRNAs between *O. barthii* and *O. glaberrima*, associated with differential expression of their regulators, namely *miR2118* and *MEL1*. This is suggestive of a differential rate of determinate meristem fate acquisition during the initial panicle development stages between the two species.

## Results

### Panicle-associated small RNAs in African rice species

To conduct a comprehensive survey of panicle-derived small RNAs in African rice and characterise qualitative and quantitative differences between the two species, we compared small RNA populations in panicles of *O. glaberrima* and *O. barthii*, using genome-wide small RNA-seq analysis. In order to focus on differences in expression resulting from inter-specific variations and to buffer genotypic variations, we used two RNA bulks extracted from developing panicles at same visual stage (from early rachis elongation to early floret differentiation stages) of 10 genotypes for each species (Additional file 1: Table S1). Over 33.1 and 33.9 million high quality reads were generated from *O. glaberrima* and *O. barthii* libraries respectively (Additional file 1: Table S2). A total of 64 % and 64.5 % of small RNA clusters (distinct small RNAs) ranging from 18 to 28 nucleotides from *O. barthii* and *O. glaberrima*, respectively, were mapped to the reference genome *O. sativa* ssp *japonica* cv Nipponbare MSU v7.0, and were found to be similarly distributed over the *O. sativa* genome (Additional file 1: Table S3, Additional files 2 and 3). As expected, the 21- and 24-nucleotide (nt) small RNAs were the predominant populations of small RNAs in the two species, with a higher number of distinct 24-nt small RNAs (Additional file 2). The high quality small RNA sequences were then categorized, using a BLAST-based filtering annotation pipeline (Additional file 4), into five distinct classes corresponding to the different functional compartments of the rice genome: miRNAs, ncRNAs, repeats, genes (CDS, intron, UTR) and unannotated regions of *O. sativa* reference genome (Additional file 1: Table S4).

**Fig. 1:**
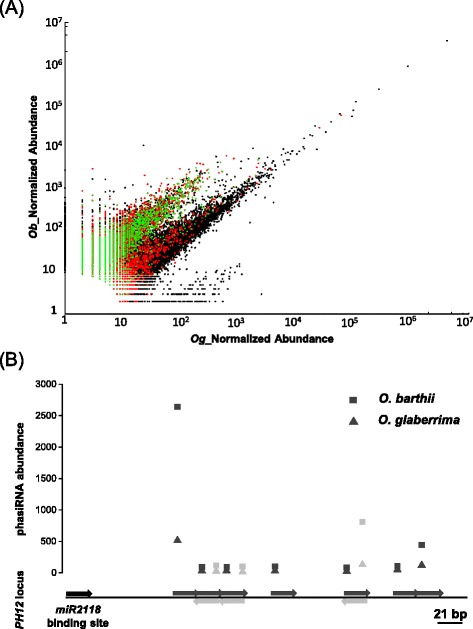
Relative abundance of panicle-derived 21-nt small RNAs from *O. barthii* and *O. glaberrima*. **a** Relative abundance of 21-nt small RNAs between *O. barthii* (*Ob*) and *O. glaberrima* (*Og*). Normalized abundance of distinct small RNA sequences. Black dots represent global 21-nt small RNAs, red dots unannotated 21-nt small RNAs, and green dots 21-nt small RNAs associated with detected phased loci. **b** Relative abundance of detected phasiRNAs generated from a single locus (*PH-12*) between *O. barthii* and *O. glaberrima.* The lower panel illustrates the position of the *miR2118* binding site and the detected phasiRNAs from the *PH12* locus in the two species (above or below the line if originating from plus or minus strand respectively). The upper panel illustrates the abundance of the corresponding phasiRNAs in *O. glaberrima* (triangles) and *O. barthii* (squares). The abundances of reads from the plus strand are in dark grey, and reads from the minus strand are in light grey

The 21-nt small RNA population from the two species, corresponding to 23 798 distinct sequences, exhibited a surprisingly large fraction of small RNAs with a higher expression in *O. barthii* than in *O. glaberrima* (Fig. 1a). This subpopulation corresponds to 29 % of the 21-nt small RNAs mapped, when considering small RNAs that were at least 5-fold more accumulated in *O. barthii*. Other size classes (from 18- to 28-nt, except 21-nt) were not distinguished by such a pattern of distribution between the two species (Additional file 5). The overall 21-nt small RNAs were categorized into five distinct classes corresponding to the different functional compartments of the rice genome: miRNAs (11.3 %), ncRNAs (13.1 %), repeats (12.4 %), genes (CDS, intron, UTR: 25.5 %) and unannotated regions (37.7 %) of *O. sativa* reference genome. However, the distribution of the over-expressed fraction in the wild species showed a strong bias towards unannotated regions (81.4 %; *p*-value = 0.0) with the remaining classes all severely under-represented: miRNAs (2.5 %; *p*-value = 8.0 × 10^−139^), ncRNAs (0.6 %; *p*-value = 3.5 × 10^−301^), repeats (0.8 %; *p*-value = 2.3 × 10^−261^) and genes (14.8 %; *p*-value = 1.2 × 10^−83^) (Fig. 1a; Additional file 5). This indicated that the over-expressed 21-nt small RNAs detected in *O. barthii* panicles were mainly derived from unannotated regions of the *O. sativa* reference genome (Fig. 1a; Additional file 5).

### 21-nt phasiRNAs are over-represented in African wild rice transcriptomes

Previous studies of *O. sativa* showed that the *miR2118*-triggered 21-nt phasiRNAs specifically expressed in the rice panicle originate from unannotated regions of the genome (Johnson et al. 2009; Song et al. 2012a; Komiya et al. 2014). To determine whether the over-represented 21-nt small RNA fraction in *O. barthii* also corresponds to this class of 21-nt phasiRNAs, we used a dedicated program (see Material and methods), and detected 4 100 distinct phasiRNAs from *O. barthii* and *O. glaberrima*, distributed across 892 loci (denoted “phased loci”), using the *O. sativa* genome as reference (Additional file 6). In addition, 952 distinct 21-nt small RNAs mapped to these 892 loci, but were not detected as phased siRNAs by our procedure. In total, 5 052 distinct 21-nt small RNA sequences associated with these 892 phased loci were found, corresponding to 21.2 % of the panicle-derived 21-nt small RNA population from the two species. Out of these 5 052 small RNAs, 3 694 were detected in both species, 1 352 were detected only in *O. barthii* and six only in *O. glaberrima* (Additional file 6). As previously reported, the detected phased loci were present on all 12 chromosomes but not at the same density, and were organized in clusters or super-clusters, such as on chromosome 12 (Additional file 7). As expected, 86.5 % of these 21-nt phasiRNAs mapped to unannotated regions of the *O. sativa* genome. This fraction of 21-nt phasiRNAs represents 49 % of the unannotated 21-nt small RNA population (4 418 over 8 977 sequences). The 892 phased loci were scanned for the presence of the 22-nt miRNA *miR2118* recognition site in their vicinity (Johnson et al. 2009; Song et al. 2012a; Komiya et al. 2014). 529 phased loci (59.3 %) shared the conserved motif close to one end (from two to 452 bp from the end). Between two and 91 distinct sequences were detected per phased locus, with an average of 6.0 (±4.8, SD) distinct sequences, the majority being 3 or 5 sequences per phased locus (Additional file 5). As expected for double strand RNA processed small RNAs, most of the detected phased loci generated small RNAs from both strands (92.5 %) (Additional file 6).

For 96 % of the phased loci, there was a significant difference in read counts between the two species, and about 71 % of them displayed an abundance ratio higher than 5 (Additional file 6). These 21-nt siRNAs were mainly over-accumulated in *O. barthii* and contributed to 52 % of the *O. barthii* five fold over-accumulated 21-nt small RNAs (Fig. 1a). The abundance of different phasiRNAs from a given locus was variable, with the predominance of one or two phasiRNAs (Fig. 1b; Additional file 8). For the differentially expressed loci, this unequal distribution over the locus was conserved between the two species, with over-accumulation of all the detected phasiRNAs from a single locus (Fig. 1b). Interestingly, the proportion of phased loci sharing the *miR2118* recognition site was significantly higher (*p*-value = 1.5 × 10^−08^) in the differentially expressed group (i.e. higher expression in *O. barthii*) (61 %, *n* = 854) than in the group of non-differentially expressed phased loci (16 %, *n* = 38) (Additional file 6). Therefore, phased loci over-expression in *O. barthii* versus *O. glaberrima* may result from differences in *miR2118* regulation between the two species.

### Panicle-associated microRNAs in African rice species

Comprehensive analysis of conserved miRNAs led to the identification of 146 annotated miRNA families expressed in African rice panicles, including 62 canonical families, 20 variant ones and 64 siRNA-like ones, according to the classification of Jeong et al. (2011) (Additional files 6 and 9). Some of the siRNA-like families were previously reported to be associated with transposable elements (TEs) in *O. sativa*, namely *TE-MIRs* (Li et al. 2011b; Yan et al. 2011). In the same way, other annotated *MIR* loci were evidenced in our analysis of 21-nt phased loci as overlapping loci, suggesting that these loci are phasiRNA-generating loci rather than *MIR* ones: *osa*-*MIR5486, osa-MIR5488*, *osa*-*MIR5506*, *osa*-*MIR5514*, osa-*miR5516*, osa-*miR5519*, *osa*-*MIR5517*, *osa*-*MIR5527*, *osa*-*MIR5530*, *osa*-*MIR5791*, *osa*-*MIR5796*, *osa-MIR5800* and *osa*-*MIR5822* (Additional file 6). Thus, the annotations of these two classes of *MIR* loci (i.e. variant and siRNA-like) should be reconsidered. Similarly to what was reported for *O. sativa* (Jeong et al. 2011), the canonical and variant miRNA families expressed in African rice panicles can be classified according to the length of the highest accumulated small RNA: length ranging from 19- to 25-nt, with 3 main types corresponding to 21-nt (48 %), 24-nt (31 %) and 22-nt (9 %) long mature miRNAs (Additional files 6 and 9).

**Fig. 2:**
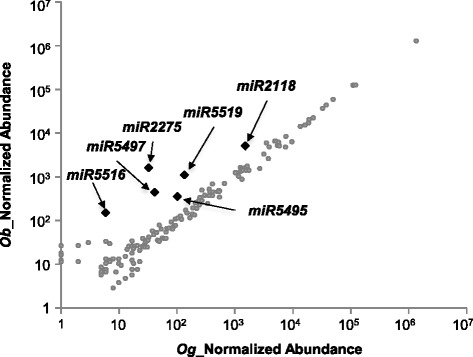
Relative abundance of miRNA families between *O. barthii* (*Ob*) and *O. glaberrima (Og)*. Normalized abundance of miRNA reads. Black diamonds represent the differentially expressed miRNA families, and grey points to non-differentially expressed miRNA families between *O. barthii* and *O. glaberrima*

Most miRNA families were expressed at similar levels in *O. barthii* and *O. glaberrima.* Interestingly, the *miR159/319* families contributed to 64 % of mature miRNA expressed in young panicles of both African species (Additional file 6), in contrast to previous studies of panicle-derived miRNAs in *O. sativa* (Jeong et al. 2011; Peng et al. 2011). This may be related to the specific developmental stages used in our study or may reflect differences between Asian and African rice species. Differential expression affected only a few miRNA families with a trend towards higher expression in *O. barthii* (Fig. 2). These families included the canonical 22-nt *miR2118* and five other ones, namely *miR2275*, *miR5495*, *miR5497*, *miR5516* and *miR5519* (Fig. 2). The latter two were identified in our study as panicle-expressed 21-nt phasiRNAs (Fig. 2; Additional file 6). The canonical 22-nt miRNA *miR2275* was previously reported to trigger 24-nt phasiRNAs (Johnson et al. 2009; Song et al. 2012a). However, despite over-expression of *miR2275* in *O. barthii,* no major over-expression of a large subset of the 24-nt small RNAs was detected (Additional file 5). The canonical miRNAs *miR5495* and *miR5497* were previously shown in *O. sativa* to be miRNAs specifically expressed in pollen (Wei et al. 2011).

### Variations in the expression of *miR2118*, 21-nt phasiRNAs and associated precursors during African rice panicle development

**Fig. 3:**
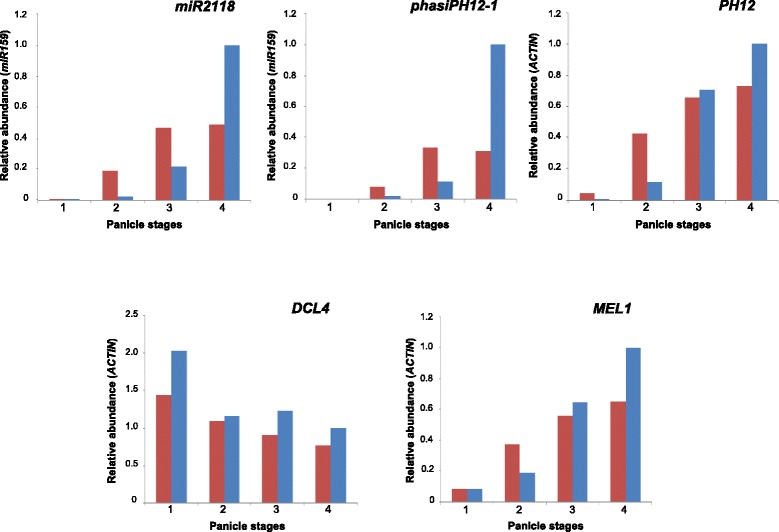
Time course expression analysis of small RNAs and related-regulators during panicle development. qRT-PCR analysis of *miR2118*, *PH12* precursor, *phasiPH12-1*, *DCL4* and *MEL1* ortholog expression levels during panicle development (from stage 1 to stage 4) in *O. barthii* (red) and *O. glaberrima* (blue). Expression values are relative to *O. glaberrima* stage 4 and the gene or miRNA used as reference is indicated. Stage 1, Inflorescence meristem stage, after initiation of primary branch (i.e. rachis and primary branch meristems); stage 2, early branching stage (panicle with elongated primary and higher order branch development); stage 3, late branching stage (i.e. panicle with elongated primary and secondary branches); stage 4, young flowers with differentiated organs

The differential accumulation of specific microRNAs and 21-nt phasiRNAs observed between *O. barthii* and *O. glaberrima* may result from differences in their expression levels, but may also reflect a shift in the timing of their expression during panicle development. To test these hypotheses, expression analysis of *miR2118,* 21-nt phasiRNAs and their RNA precursors was performed on 4 distinct morphological stages of panicles collected from *O. barthii* (accession B88) and *O. glaberrima* (variety CG14) (Fig. 3; see Additional file 10 for histological description). Four different phased loci from distinct clusters and chromosomes were considered for the RNA precursors as well as the phasiRNA with the highest level of accumulation for each locus (Fig. 3; Additional files 8, 11 and 12).

The differential accumulation of *miR2118*, selected phasiRNAs and their corresponding precursors between the two species were validated through Northern-blotting and/or RT-PCRs on RNA bulks similar to the ones used for small RNA deep sequencing (Additional file 8). While neither mature *miR2118* nor 21-nt phasiRNA *phasiPH12-1* were detected at the early morphological stage of panicle development (inflorescence meristem after reproductive transition, stage 1), their expression was initiated earlier in *O. barthii* (early branching stage, stage 2) than in *O. glaberrima* (late branching stage, stage 3). The expression level reached a peak at stage 3 in *O. barthii* and remained at the same level at stage 4 (young flowers with differentiated organs). In *O. glaberrima* the miRNA and phasiRNA were more abundant at stage 4 and displayed a higher expression level than in *O. barthii* at this stage (Fig. 3). Similar patterns of expression were observed during panicle development for 21-nt phasiRNAs from the four phased loci tested (Additional file 12). These findings indicate that the later accumulation of 21-nt phasiRNAs during the panicle developmental time-course in *O. glaberrima* is associated with a later accumulation of *miR2118*. These findings are in agreement with the small RNA-seq data, as the RNA bulks used for sequencing originate from panicles at morphological stages 1 to 3, in which *miR2118* and 21-nt phasiRNAs are accumulated at higher levels in *O. barthii* than in *O. glaberrima*.

Similarly to Komiya et al. (2014), we showed that the 21-nt phasiRNAs from the African species were generated from polyA-tailed long non-coding RNAs (lncRNAs) (Additional file 11). The accumulation patterns of these polyA-tailed lncRNAs were similar to those of the phasiRNAs with a later initiation of expression in *O. glaberrima* than in *O. barthii,* regarding the morphological stages (Fig. 3; Additional file 12). Furthermore, we investigated the accumulation levels of mRNAs corresponding to gene orthologs encoding other factors involved in the biogenesis of the *miR2118*-triggered 21-nt phased RNAs, such as the OsDCL4 DICER-like protein, and the gamete-specific *Argonaute* protein MEL1 (Song et al. 2012a, b; Komiya et al. 2014). The accumulation level of African rice *OsDCL4* ortholog mRNAs decreases slightly over the 4 stages, while African rice *MEL1* ortholog mRNA accumulation increases from stage 1 to stage 4 in a similar pattern to the small RNAs and lncRNAs (Fig. 3; Additional file 12). Taken together, these data suggest that the accumulation levels of phasiRNAs may depend on *miR2118,* phased loci-associated lncRNAs and African rice *MEL1* ortholog transcript accumulation levels rather than to those of the *OsDCL4* ortholog*.*

**Fig. 4:**
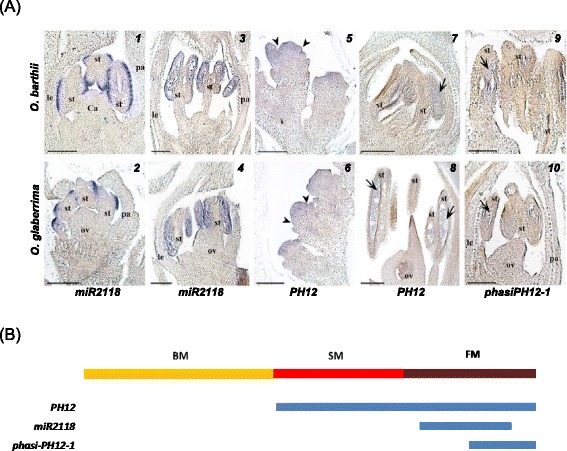
*In planta* expression analysis of small RNAs and related-regulators during panicle development. **a** *In situ* hybridization of mature *miR2118* (1–4), *PH12* mRNA precursor (5–8) and *phasiPH12-1* (9–10) in spikelet meristem (SMs), floret meristem (FMs) and differentiated floret in *O. barthii* (B88) and *O. glaberrima* (CG14). Mature *miR2118* transcripts were detected in the outer cell layer of stamens (*i.e.* hypodermis) (1–2) and later in pollen sac (3–4). Precursor *PH12* mRNAs were detected in the spikelet meristem (5–6) and in pollen sac of the anthers (arrow) (7–8). The *phasiPH12-1* small RNAs were detected in the pollen sac of the anthers in florets (9–10). This panel of photos illustrates stages for which the transcripts were detected. le, lemma; pa, palea; st, stamen; ca, carpel; ov, ovule. Arrowheads, lemma and palea primordium formation in SMs; arrow, anthers. Scale bars: 100 μm. **b** Schematic view of the expression profiling of *PH12* mRNA precursor, mature *miR2118* and *phasiPH12-1* regarding meristem state and developmental timing based on *in situ* hybridization data. Blue boxes illustrate transcript detection periods. BM: branch meristem; SM: spikelet meristem; FM: floret meristem

To determine the spatial expression patterns of *miR2118, phasiPH12-1* and *PH12* lncRNAs during panicle development, *in situ* hybridization analysis was performed at different developmental stages of young panicles from the two species (Fig. 4). Similar patterns were observed for the two species with variations in spatial patterning of these factors. This analysis revealed that the onset of the *miR2118*-triggered 21-nt phasiRNA pathway is sequential in terms of initiation of expression. *PH12* lncRNAs are detected first in spikelet meristems and are maintained in developing florets, but only in stamens and more specifically in the pollen sac. Subsequently, *miR2118* specifically accumulates in the hypodermis of developing stamens (prior to pollen sac differentiation) at the early floret differentiation stage, then in the epidermis and the pollen sac in differentiated stamens at later stages. Finally, the *phasiPH12-1* phasiRNAs are specifically located in pollen sac of differentiated stamens subsequent to *miR2118* detection (Fig. 4). Eventually, all three signals were observed at the same location at later stages (i.e. pollen sac).

Our spatial expression analysis of *miR2118*, *phasiPH12-1* and *PH12* lncRNA and their differential accumulation patterns with respect to the morphological stages of panicle development between the two species indicates that at the branching stage, the gamete-associated small RNAs are initiated later and expressed at lower levels in *O. glaberrima* than in *O. barthii*. This suggests that for the same morphological stage of panicle development, stamen differentiation, and therefore floret differentiation, might be delayed in *O. glaberrima* with respect to its wild relative, *O. barthii*.

### Variations in expression of a spikelet-related gene during panicle development

**Fig. 5:**
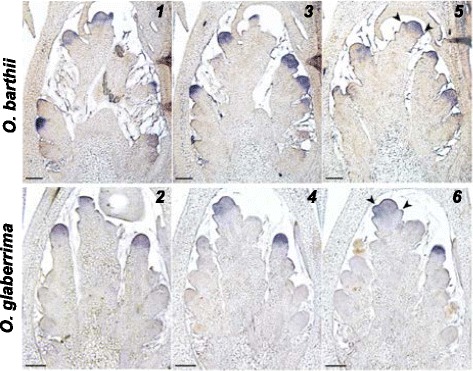
*In situ* expression analysis of *LHS1* gene in young panicles from African rice. *In situ* hybridization of *LHS1* transcripts in serial sections of spikelet meristems (SMs) of *O. barthii* (1–3) and *O. glaberrima* (4–6); arrowheads, lemma and palea primordium formation in SMs. Scale bars: 100 μm

To investigate this hypothesis further, we analysed the expression pattern of orthologs of the *O. sativa* E-function MADS-box *LEAFY HULL STERILE1* (*LHS1)/OsMADS1* gene that promotes the determination of meristems (i.e. spikelet and floret meristems) (Jeon et al. 2000; Cui et al. 2010; Khanday et al. 2013). Using *in situ* hybridization, we investigated the expression pattern of the *LHS1* ortholog in *O. barthii* and *O. glaberrima* panicles at the early branching stage. In the two species*, LHS1* mRNA was detected specifically in SMs (Fig. 5). However, while the *LHS1* mRNA signal was observed in some lateral meristems in *O. glaberrima*, it was detected in all lateral meristems in *O. barthii* (Fig. 5). This supports the hypothesis that for the same morphological stage, all terminal and lateral meristems had acquired the spikelet fate in *O. barthii*, whereas in *O. glaberrima* few had done so. These observations are also in agreement with the gamete-associated small RNA profiling data obtained.

## Discussion

### Conservation of *miR2118*-triggered 21-nt phasiRNAs in Oryza species

Using panicle-derived small RNA transcriptome sequencing in *O. glaberrima* and its wild ancestor, *O. barthii,* we showed that 29 % of the 21-nt small RNA population is drastically repressed (or non activated) in cultivated genotypes during the branching stage of panicle development. This large difference in expression pattern suggests a change in regulation of key regulatory molecules(s). The altered fraction of small RNAs corresponds mainly to 21-nt phasiRNAs generated from nearly a thousand non-coding RNA loci, associated with altered expression of the 22-nt microRNA *miR2118*. This is in agreement with observations in *O. sativa* that panicle-specific 21-nt phasiRNAs are produced from multiple non-coding RNA loci through an RDR6/DCL4-dependent pathway triggered by the microRNA *miR2118* (Johnson et al. 2009; Song et al. 2012a, b). The synthesis of secondary siRNAs (or phasiRNAs) in reproductive organs from non-coding loci has only been reported in rice, maize and *Brachypodium*, suggesting a recent origin of these secondary siRNAs from a common ancestor of grasses (Zheng et al. 2015; Johnson et al. 2009; Song et al. 2012a, b; Zhai et al. 2015, Vogel et al. 2010). However, the low conservation of these loci across the group suggests that they may have species-specific functions (Zheng et al. 2015;, Komiya et al. 2014). Interestingly, *miR2118*-triggered secondary siRNA synthesis is also conserved across distantly related species, such as *Medicago truncatula*, tobacco and tomato. However, in these eudicot species, the *miR2118* family members (including *miR482* family members) were recruited to initiate secondary siRNAs on mRNAs encoding NBS-LRR proteins involved in disease resistance (Zhai et al. 2011; Li et al. 2012; Shivaprasad *et al.*^2012^).

Our study revealed conserved features of panicle-associated 21-nt phasiRNAs and associated loci between *Oryza* species. However, this analysis was limited to the fraction of small RNAs corresponding to *O. barthii* and *O. glaberrima* phasiRNAs conserved in *O. sativa* (i.e. mapped to the *O. sativa* genome), and does not provide a complete overview of the complexity of phased loci in African rice species and their evolution during the African domestication process. The genome sequence of *O. glaberrima* accession CG14 was recently published (Wang et al. 2014), however, we obtained better mapping results from our small RNA sequence data using the reference genome *O. sativa* ssp *japonica* cv Nipponbare MSU v7.0 than the *O. glaberrima* CG14 released sequence: 72.1 % of small RNA reads from both African species using Nipponbare MSU v7.0 sequence in contrast to 61.6 % and 56.2 % from *O. barthii* and *O. glaberrima* respectively using the *O. glaberrima* reference genome AGl1.1. For this reason, we continued to perform our small RNA sequence data analysis using the *O. sativa* Nipponbare reference genome. Nevertheless, it might be expected that higher diversification occurred between Asian and African rice since they diverged about 1 million years ago, whereas *O. glaberrima* and *O. barthii* diverged about 3000 years ago. Consequently, our quantitative analysis in African rice will be only marginally biased by evolutionary differences at the sequence level between the two African rice species. However, the number of overlapping phased loci between African rice species and *O. sativa* is quite low. About 1136 phased loci were identified from 4-cm long panicles of *O. sativa* ssp *indica* var 93–11. However, only 416 out of the 892 detected phased loci in African species overlapped phased loci detected in *O. sativa* spp *indica* var 93–11 (Song et al. 2012a). This may be because they were identified in a different way, i.e. based on different stages of panicle development. It should also be noted that more small RNA sequences were characterized in our study. Even if sequences are conserved between *Oryza* species, it cannot be ruled out that species-specific expression patterns of these lncRNA loci may occur. Moreover, 40 % of the detected loci associated with differentially expressed 21-nt phasiRNAs between the two African species had no *miR2118* recognition site motif, suggesting a potentially distinct mechanism of production. Another possible explanation would be that *miR2118* recognition sites are present in the African rice genome at these loci but not in the *O. sativa* genome. In addition, in our comparative analysis, 48 % of the over-expressed 21-nt small RNAs in *O. barthii* mapping to the *O. sativa* genome were not associated with the detected phased loci, and also originated mainly from unannotated regions of the genome. It is possible that these remaining over-expressed 21-nt small RNAs belong to phased loci specific to African rice genomes that were not detectable in our analysis because of sequence divergence from the *O. sativa* reference, or that they are associated with non-coding loci unrelated to phased small RNA generating loci. Recently, Zhang et al. (2014b) showed that a large set of long non-coding RNAs was expressed in anthers and pistils of *O. sativa* rice. Interestingly, only 122 of the 1624 reported lncRNA loci were associated with small RNAs (Zhang et al. 2014b). In agreement with this, only a few of the reported lncRNA loci overlapped with the *miR2118*-associated phasiRNA-generating loci in *O. sativa* and African rice species (data not shown). This suggests that these lncRNAs are mostly not associated with the production of phasiRNAs or other small RNAs and may act through other mechanisms during plant development.

### The *miR2118*-triggered 21-nt phasiRNAs are markers of male-gametogenesis in African rice

The function of the 21-nt phasiRNAs during panicle development is still unclear. MicroRNA *miR2118* has been reported to be preferentially expressed in rice and maize stamens, suggesting a role in male gametogenesis (Song et al. 2012a). Recently, it was shown that the function of this class of phased small RNA was dependent on the germ line-specific Argonaute (AGO) protein MEL1 through a direct interaction between MEL1 and phasiRNAs (Komiya et al. 2014). Moreover, as mature *miR2118* was detected in the MEL1-binding small RNA fraction, it was argued that MEL1 might play a role in the first steps of the 21-nt phasiRNA biogenesis pathway as the AGO protein in the *miR2118*-driven RISC triggering to the first cleavage of lncRNA precursors (Komiya et al. 2014). This is in agreement with the expression pattern observed for *MEL1* and *miR2118. MEL1* mRNAs were observed to be first detectable by *in situ* hybridization in the hypodermis of developing stamen primordia in a spotty pattern, similarly to that which we observed for *miR2118* microRNA, later becoming restricted to microsporangia and pollen sac (Nonomura et al. 2007). In our study, while *PH12* precursor lncRNAs were detected in the spikelet meristem differentiation stage, *phasiPH12-1* was only detected in the stamens of differentiating florets and co-localized with *PH12* precursor lncRNAs, but only after *miR2118* was observed to accumulate. The latter was first limited to the hypodermis of differentiating stamens and extended to the pollen sac in later stages. Together, these data suggest that the 21-nt phasiRNA regulatory network is initiated early during panicle development from the stage of spikelet meristem establishment, before *miR2118* and *MEL1* expression, through the induction of lncRNA precursor expression. The co-expression of *miR2118* and *MEL1* in the hypodermis of the differentiating stamen would result in the initiation of the 21-nt phasiRNA biogenesis pathway, leading to the accumulation of the phasiRNAs. However, the *trans-*acting factors involved in the regulation of the lncRNA precursors are still unknown. Zhai et al. (2015) have recently shown that maize *miR2118*-triggered 21-nt phasiRNAs correspond to premeiotic siRNAs in anthers, with a similar spatial patterning observed in rice, indicating a conservation of the spatial regulation of this pathway in grasses.

Although direct evidence is still lacking for the involvement in male gametogenesis of *miR2118* and the associated phasiRNAs and lncRNA precursors, their involvement is supported by the phenotypes of *rdr6* and *dcl4* mutants affected in flower and stamen development (Liu et al. 2007; Song et al. 2012b). Moreover the *mel1* mutant phenotype indicates that the MEL1 AGO protein mediates the regulation of germ-line mother cell development and meiosis in both male and female organs, suggesting that the MEL1-phasiRNA complexes may be involved in these processes (Nonomura et al. 2007; Komiya et al. 2014). The remaining question is whether these phasiRNAs can be considered as *ta*-siRNA (secondary siRNAs targeting other mRNAs). The predominant accumulation of MEL1 protein in the cytoplasm would favour a *trans*-acting function of MEL1-phasiRNA complex on mRNAs but without cleavage activity of the putative targets (Komiya et al. 2014; Zhai et al. 2015).

### Differential rate of determinate fate acquisition in the panicle meristems of African rice

Our comparative analysis of small RNA expression with respect to the development of the African rice panicle suggests that the spikelet/floret fate acquisition rate differs between the two species: for a similar morphological complexity during the early branching stage, all meristems are converted into spikelets in *O. barthii* whereas only those of the apical part of the panicle branches are converted in *O. glaberrima,* as suggested by the expression pattern of the spikelet-associated MADS-box gene *LHS1/OsMADS1*. This difference was paralleled by the later initiation of expression and lower accumulation during the branching stage of the 21-nt phasiRNA pathway members (*miR2118*, *MEL1*, *lncRNAs* and *phasiRNAs*) in *O. glaberrima*, as well as the lncRNA precursors expressed in spikelet meristems. The differential rate of spikelet transition observed between the two species may be explained by a shorter duration of development in *O. barthii* than in *O. glaberrima* but also possibly by the synchronous transition of the apical and lateral meristems from the indeterminate to the determinate state in *O. barthii*.

Theoretical modelling of inflorescence architecture suggests that inflorescence branching complexity and its evolution depends on differences in the timing of floral (i.e. determinate) fate acquisition in apical and/or lateral meristems (Prusinkiewicz et al. 2007). This biological basis is supported by the analysis of various mutants affected in floral meristem identity in different species, notably in *Arabidopsis thaliana*, *Antirrhinum majus*, petunia and tomato (Koes 2008; Moyroud et al. 2010; Park et al. 2014). A comparative study of the diversity of inflorescence architecture in tomato based on meristem-specific transcriptome analysis provided support for this model (Park et al. 2012). In the case of the grass inflorescence and more specifically the rice panicle, it was also reported that variations in panicle architecture depended on the activity or expression levels of both meristem fate controlling genes and branch-promoting genes (Kyozuka et al. 2014; Zhang and Yuan 2014). In the context of our comparative analysis of African rice species, a differential rate of determinate fate acquisition in panicle meristems would result in a longer or higher rate of branching activity in *O. glaberrima* compared to *O. barthii*, leading to a higher branch complexity in the domesticated species. However, further functional studies are required to define the link between the major regulatory changes of spikelet-related gene expression and the panicle phenotypic variations associated with domestication.

## Conclusions

We provide evidence that the male-gametogenesis-specific 21-nt phasiRNA pathway triggered by *miR2118* is conserved in both wild relative and domesticated African rice species. Our study shows that the onset of the *miR2118*-triggered 21-nt phasiRNA pathway is sequential in terms of initiation of expression with variations in spatial patterning of these factors. This pathway is initiated when spikelet meristems are established, through the activation of lncRNA precursor expression. Our study provides evidence that the differential expression of the *miR2118*-triggered 21-nt phasiRNA pathway in *O. barthii* and *O. glaberrima* may be associated with different rates of determinate meristem fate acquisition during branching stage of panicle development. It will be of great interest to determine whether a similar scenario has occurred in the Asian rice species (*O. sativa* vs*. O. rufipogon*), so as to determine whether or not the phenotypic convergence of panicle development observed between the two domestication processes is paralleled by similar changes in the expression of molecular regulatory components associated with spikelet differentiation and panicle architecture complexity.

## Methods

### Plant materials and panicle sampling

For Illumina sequencing, 10 accessions of *O. glaberrima* and 10 accessions of *O. barthii* (see Additional file 1: Table S1) were grown in the greenhouse at IRD, Montpellier. Around 15 panicles from each accession were collected from 4 to 15 days after induction, to obtain homogeneous representation of early developmental stages corresponding to early rachis elongation to early floret differentiation.

For histological analysis and expression analysis, CG14 and B88 plants were grown in growth chamber at IRD, Montpellier (France) with a 14-10 h day/night cycle at 32 °C/28 °C and humidity at 60 %. Flowering was induced by short day conditions (10-14 h day/night cycle). Panicles were collected at 4 different morphological stages: stage 1, inflorescence meristem stage, after initiation of primary branch (i.e. rachis and primary branch meristems); stage 2, early branching stage (i.e. panicle with elongated primary and higher order branch development); stage 3, late branching stage (i.e. panicle with elongated primary and secondary branches); stage 4, young flowers with differentiated organs (Additional file 10).

### Illumina sequencing and data processing

Total RNAs (including small RNAs) were extracted using an RNeasy Plant Mini Kit with RLT and RWT buffers (Qiagen, France). DNase treatments were performed using the RNAeasy-free DNase set (Qiagen, France). Two bulks of total RNAs corresponding to a mix of total RNAs from the 10 accessions of the two species were used for sequencing. Purified small RNA sequencing was performed by Eurofins/MWG Operon (Germany) on an Illumina *Hi-seq 2000* using the TruSeq™ SBS v5 sequencing kit. The complete raw dataset is available in NCBI Gene Expression Omnibus repository [GEO: GSE48346]. The raw data were trimmed by removing adapter sequences and low quality sequences using *CutAdapt* (Martin 2011). All the trimmed reads ranging from 18 to 28 nucleotides were clustered and mapped to *O. sativa ssp japonica* cv Nipponbare genome (MSU release version 7; http://rice.plantbiology.msu.edu/) using *BLAST* (Altschul et al. 1990) (Additional file 4) in order to evaluate library quality. The 18–28 nucleotide reads were them annotated through a filtering process using successive hierarchical *BLAST* (Additional file 4) versus (in order) miRBase v17.0 (Kozomara and Griffiths-Jones 2011), Rfam v7, a home-made repeat database (successive curated concatenation of *RetrOryza*, RepBase, TREP and TIGRRepeats), CDS then gene features (i.e. introns and UTRs) from *Oryza sativa ssp japonica var* Nipponbare MSU v7.0 annotation, and finally the MSU v7.0 rice genome for the sequences not similar to miRNAs, non coding RNAs, Repeat-associated sequences and genes. BLAST filtering was done using *Oryza* genus data form the different databases (i.e. mainly *O. sativa*). Mapping of the sequences to the *O. sativa* MSU v7.0 reference genome was done for the last three steps of our annotation pipeline. This genome was used as a reference because (*i*) of its equal divergence to the two species we analyzed (avoiding favoring one species in mapping analyses rather than the other one), and (*ii*) because of the larger resources in terms of annotation, expression data and genomic information available for this species. The *BLAST* and post-filter parameters used were probability of 85 %, e-value of 10^−3^, on a size of 85 % of the reads (minimum size of 16). The same *BLAST* parameters were used throughout the analysis. Mapping from *O. glaberrima* and *O. barthii* small RNA sequences was then compared and filtered using a series of homemade *Perl* scripts (available on demand). Comprehensive analysis of the miRNA-associated small RNAs from African rice species was limited to the families present in *O. sativa*, and did not include diverse isoforms. For the same reason, potential African rice specific miRNAs were not investigated. The 21-mers were used in phasing analysis with the *ta-si Prediction* tool from the *UEA sRNA workbench* facilities (http://srna-workbench.cmp.uea.ac.uk/; Stocks et al. 2012). A post-analysis manual filtering was performed for sequences mapping to more than 10 loci in genome, as well as telomere-associated loci, leading to the deletion of 123 loci from the 1015 initial loci reported in *ta-si Prediction* tool outputs. Once the loci were identified, we used the *EMBOSS* software suite v6.5.7.0 (Rice et al. 2000) to extract −500/+500 bases around each locus, and treated them using *MEME* v4.8.1 (Bailey and Elkan 1994). Then*,* the loci sequences were used as a BLAST database to add other 21-nt sequences that were not identified originally as phased (in order to by-pass the genome divergence), in two pass: first we select only new sequences with 100 % of identity on 85 % of their length (minimum HSP length of 16), then we change the threshold to 85 % of identity on 85 % of their length (min HSP length of 16). Finally, statistical tests of all the processed data were performed using *g-test* and a fixed *p*-value of 10^−3^. Depending on the experiment, the degree of freedom was adjusted but was generally 1. All the calculations were performed using homemade *Perl* scripts and CPAN statistical modules.

### Quantitative RT-PCR analysis

Total RNAs (including small RNAs) were extracted as described before, from the 4 individual morphological stages of young panicles of *O. glaberrima* (CG14) and *O. barthii* (B88). First-stand cDNA was synthesised using SuperScript III cDNA First-strand synthesis system (Invitrogen). Quantitative stem-loop RT-PCR analyses on small RNAs were performed using 100 ng of total RNA according to Varkonyi-Gasic et al. (2007) with reverse transcription at 42 °C in conjunction with small RNA-specific stem-loop primers. Quantitative RT-PCR analyses on mRNAs were performed using 1 μg of total RNA in conjunction with polydT or random hexamer primers according to the manufacturer’s instructions. qRT-PCRs were performed using LightCycler 480 thermocycler (Roche, France) in conjunction with SYBR Green I master mix (Roche, France) in 8 μL reaction mix containing 2 μL of diluted RTs and 0.8 μL of forward and reverse primers at 10 μM. The qPCR amplification conditions include 3 stages: pre-incubation (95 °C in 10 min); amplification with 45 cycles (95 °C 15 s and 60 °C 30s); melting curve (95 °C 5 s and 70 °C 1 min). In stem-loop qRT-PCR, the levels of miRNA were normalized by mature *miR159* expression level. In classic qRT-PCR, mRNAs were normalized to the rice *Actin* gene (*LOC_Os03g50885.1*). Each set of experiments was repeated three times, and the relative quantification method with efficiency corrected calculation model (Souaze et al. 1996) was used to evaluate quantitative variations. The primers used are listed in Additional file 1: Table S5.

### *In situ* hybridizations

PCR amplifications were performed with gene-specific antisense primers tailed with a T7 RNA polymerase binding site (see Additional file 1: Table S5 for primer sequences). The resulting DNA fragments were used directly as templates for synthesizing antisense ribo-probes incorporating UTP–digoxigenin (Roche) as the label in conjunction with a T7 Maxi Script kit (Ambion). For *miR2118*, *PH12* precursor and *phasiPH12-1* detection, 0.02 μM of a 5′ digoxigenin–labeled LNA probe complementary to the target (see Additional file 1: Table S3 for primer sequences) was used. *In situ* hybridization experiments were carried out as described by Adam et al. (2011). Detection was performed using the Vector Blue Alkaline Phosphatase Substrate Kit III (Vector Laboratories). Slides were observed and photographed by Evolution MP5.0 colour Media Cybernetics camera in conjunction with a Leica DMRB microscope and images were processed using Photoshop CS6.

## Additional files

Additional file 1: Table S1. List of genotypes used in this study. **Table S2.** Summary statistics of small RNA libraries. **Table S3.** Summary of sequence number (clusters and reads) mapped to *O. sativa* Nipponbare reference genome (MSU7.0) and *O. glaberrima* CG14 reference genome for *O. barthii* and *O. glaberrima* small RNA sequences. **Table S4.** Summary of sequence number (clusters and reads) in each annotation class (i.e. sequence databases) for *O. barthii* and *O. glaberrima* small RNA sequences based on BLAST-filtering. **Table S5.** List of primers used in this study. The bases in brackets represent the ones that were modified LNAs. The sequences in italic correspond to stem-loop region for stem-loop RT-PCRs. The underline sequences correspond to T7 RNA polymerase binding site used for RNA probe synthesis. (PDF 191 kb) Additional file 2: Size distribution, mapping and annotation of *O. barthii* and *O. glaberrima* panicle-derived small RNAs on *O. sativa Nipponbare* genome. (a) Small RNA size distribution of clusters (distinct) and reads (abundance). The size of small RNAs was plotted *versus* frequency (percentage relative to total abundance). (b) Mapping rate (percentage relative to total number) of clusters (i.e. distinct sequences) and total reads on *O. sativa nipponbare* MSU7.0. The percentage values are indicated in the bars. (c) Small RNA annotation frequency (percentage relative to total abundance) of clusters (distinct) and individual reads (abundance). The percentage values are indicated in the bars. (d) Size distribution of the annotated small RNA clusters (i.e. distinct sequences) according the annotation classes. (e) Size distribution of the annotated small RNA sequences (i.e. abundance/reads) according the annotation classes. (PDF 333 kb) Additional file 3: Genomic distribution and abundance of *O. barthii* and *O. glaberrima* panicle-derived small RNAs on *O. sativa nipponbare* genome. (a) Distribution and abundance of *O. barthii* reads vs*. O. sativa nipponbare* genome MSU7.0. (b) Distribution and abundance of *O. glaberrima* reads vs. *O. sativa nipponbare* genome MSU7.0. (PDF 1091 kb) Additional file 4: Bio-informatic workflow for small RNA analysis. (PDF 158 kb) Additional file 5: Relative abundance of small RNAs between *O. barthii* and *O. glaberrima*. (a) Relative abundance of small RNAs between *O. barthii* (*Ob*) and *O. glaberrima* (*Og*) according to their size (from 18- to 28-nt small RNAs). LogPlots of normalized abundance of distinct small RNA sequences. (b) Relative abundance of the annotation classes of 21-nt small RNAs between *O. barthii* (*Ob*) and *O. glaberrima* (*Og*). LogPlot of normalized abundance of distinct small RNA sequences. Black dots represent global 21-nt small RNAs, and red dots the class of annotated 21-nt small RNAs. (c) Relative abundance of the annotation classes of 24-nt small RNAs between *O. barthii* (*Ob*) and *O. glaberrima* (*Og*). LogPlot of normalized abundance of distinct small RNA sequences. Black dots represent whole 24-nt small RNAs, red dots represent the class of annotated 24-nt small RNAs. (PDF 1571 kb) Additional file 6: 21-nt small RNAs associated to phased loci and mature miRNA families detected in young panicles of *O. glaberrima* and *O. barthii*. (a) *O. sativa* Nipponbare loci associated with 21-nt phased and unphased siRNAs from *O. barthii* and *O. glaberrima* detected using sRNAworkbench facilities. (b) Normalized count of reads (relative abundance for 2 millions of total reads) associated with mature miRNA families in relation to small RNA size. (PDF 383 kb) Additional file 7: Genomic distribution, abundance and complexity of *O. barthii* and *O. glaberrima* 21-nt phased small RNAs on *O. sativa nipponbare* genome (MSU 7.0). (a) Genomic distribution and abundance of *O. barthii* and *O. glaberrima* 21-nt phasiRNAs on *O. sativa nipponbare* genome (MSU 7.0). (b) Distribution of number of detected 21-nt phasiRNAs from African species per phased locus on *O. sativa nipponbare* genome (MSU 7.0). (PDF 547 kb) Additional file 8: Sequences and phasiRNA abundances of the 4 phased loci used for validation. The 4 phased loci reported here were used for stem-loop RT-PCR (phasiRNAs) and classical RT-PCR (lncRNAs) validation. The positions of the phased loci on MSU7.0 chromosomes are indicated. The alignment between *miR2118f* and the phased locus is indicated. The *miR2118* recognition site is in blue. The phasiRNAs detected in the plus strand are highlighted alternately in green and in red. The phasiRNAs detected in the minus strand are underlined alternately in green and in red. The phasiRNA boxed in yellow is the most abundant one and the one used for stem-loop RT-PCR validation and qRT-PCR analysis. Histograms illustrate the relative abundance of the detected phasiRNAs per locus in the two species (PDF 159 kb) Additional file 9: Features of *O. barthii* and *O. glaberrima* panicle-derived microRNAs and abundance of sequences related to microRNA precursor sequences. (a) Size distribution of clusters (distinct) and reads (abundance) related to *O. sativa* microRNAs in *O. barthii* and *O. glaberrima*. (b) Relative abundance of *O. barthii* and *O. glaberrima* reads related to *O. sativa* microRNA precursors. The precursors are classified according to the abundance distribution pattern as defined by Jeong et al. (2011) (i.e. canonical, variant, siRNA-like). (PDF 200 kb) Additional file 10: Histological description of selected developmental stages of African rice panicles. *O. barthii*: 1,3,5,7,9,11; *O. glaberrima*: 2,4,6,8,10,12; stage 1: unbranched stage with elongation of rachis meristem (arrowheads) and formation of primary branch meristems (*) (1,2); stage 2: early branching stage with rachis meristem (arrowheads) and elongating primary branches (3,4). At the end of this stage, secondary branches (white *) are initiated from PBs (*) (5,6); stage 3: late branching stage with elongated secondary branch and spikelet meristem (SM) and floret meristem (FM) differentiation (7,8); stage 4: floret organ differentiation/development (9,10); mature stage: 11 and 12. White arrowhead: vestige of aborted rachis meristem. Scale bar: 100 μm. (PDF 1544 kb) Additional file 11: RNA-seq data validation by northern-blotting and semi-quantitative RT-PCR for miRNAs, phasiRNAs and ncRNAs associated with phased loci in panicle-derived small RNA bulks from *O. barthii* (*Ob*) and *O. glaberrima* (*Og*). (a) Northern blot hybridization using *miR2118* as probe. U6 probe was used as control. (b) Stem-loop RT- PCR analysis of various miRNAs and phasiRNAs. *miR159a* probe was used as a loading control. (c) Classic RT-PCR analysis of ncRNAs associated with 21-nt phased small RNA loci using from polydT primer (dT) or random hexamer primers (RH) for the RTs. The *ACTIN* gene (*Os03g50885*) was used as a loading control. (d) Test of specificity of stem-loop RT-PCRs against phasiRNAs on *PH12* and *PH779* loci, in conjunction with phasiRNA specific forward primer (PH12-F and PH779-F primers; phasiRNA label) and long ncRNA forward primer (PH12-F2 and PH779-F2 primers; long ncRNA label). Controls: 1. RT reaction using *O. glaberrima* RNA bulk without stem-loop RT primer; 2. RT reaction using *O. barthii* RNA bulk without stem-loop RT primer; 3. RT-PCR without stem-loop RT matrix; Ø: RT-PCR without RT. See Additional file 1: Table S5 for primer and probe sequences. (PDF 1888 kb) Additional file 12: Expression patterns of phasiRNAs and lncRNA precursors associated with 21-nucleotide phased loci in *O. glaberrima* and *O. barthii*. qRT-PCR analysis of 21-nucletiode phasiRNAs expression levels (a) and lncRNAs (b) from 3 distinct phased loci (*PH557, PH612* and *PH779*) during panicle development in *O. barthii* (red) and *O. glaberrima* (blue). Expression values are relative to *O. glaberrima* stage 4 and the *ACTIN* gene or *miR159* miRNA used as reference is indicated. Panicle stages: Stage 1, Inflorescence meristem stage, after initiation of primary branch (i.e. rachis and primary branch meristems); stage 2, early branching stage (panicle with elongated primary and higher order branch development); stage 3, late branching stage (i.e. panicle with elongated primary and secondary branches with spikelets and beginning of floret differentiation); stage 4, young flowers with differentiated organs. See Additional file 1: Table S5 for primer sequence. (PDF 56 kb)

## References

[CR1] Adam H, Marguerettaz M, Qadri R, Adroher B, Richaud F, Collin M, Thuillet AC, Vigouroux Y, Laufs P, Tregear JW, Jouannic S (2011). Divergent expression patterns of *miR164* and *CUP-SHAPED COTYLEDON* genes in palms and other monocots: implication for the evolution of meristem function in angiosperms. Mol Biol Evol.

[CR2] Allen E, Xie Z, Gustafson AM, Carrington JC (2005). MicroRNA-directed phasing during trans-acting siRNA biogenesis in plants. Cell.

[CR3] Altschul SF, Gish W, Miller W, Myers EW, Lipman DJ (1990). Basic local alignment search tool. J Mol Biol.

[CR4] Arikit S, Zhai J, Meyers BC (2013). Biogenesis and function of rice small RNAs from non-coding RNA precursors. Curr Opin Plant Biol.

[CR5] Bailey TL, Elkan C, Altman R, Brutlag D, Karp P, Lathrop R, Searls D (1994). Fitting a mixture model by expectation maximization to discover motifs in biopolymers. Proceedings of the Second International Conference on Intelligent Systems for Molecular Biology.

[CR6] Caicedo AL, Williamson SH, Hernandez RD, Boyko A, Fledel-Alon A, York TL, Polato NR, Olsen KM, Nielsen R, McCouch SR, Bustamante CD, Purugganan MD (2007). Genome-wide patterns of nucleotide polymorphism in domesticated rice. PLoS Genet.

[CR7] Carroll S (2008). Evo-Devo and an expanding evolutionary synthesis: a genetic theory of morphological evolution. Cell.

[CR8] Cui R, Han J, Zhao S, Su K, Wu F, Du X, Xu Q, Chong K, Theissen G, Meng Z (2010). Functional conservation and diversification of class E floral homeotic genes in rice (*Oryza sativa*). Plant J.

[CR9] Doebley J, Lukens L (1998). Transcriptional regulators and the evolution of plant form. Plant Cell.

[CR10] Huang X, Kurata N, Wei X, Wang Z-X, Wang A, Zhao Q, Zhao Y, Liu K, Lu H, Li W (2012). A map of rice genome variation reveals the origin of cultivated rice. Nature.

[CR11] Jeon JS, Jang S, Lee S, Nam J, Kim C, Lee SH, Chung YY, Kim SR, Lee YH, Cho YG (2000). *leafy hull sterile1* is a homeotic mutation in a rice MADS box gene affecting rice flower development. Plant cell.

[CR12] Jeong D-H, Park S, Zhai J, Gurazada SGR, De Paoli E, Meyers BC, Green PJ (2011). Massive analysis of rice small RNAs: mechanistic implications of regulated microRNAs and variants for differential target RNA cleavage. Plant cell.

[CR13] Johnson C, Kasprzewska A, Tennessen K, Fernandes J, Nan G-L, Walbot V, Sundaresan V, Vance V, Bowman LH (2009). Clusters and superclusters of phased small RNAs in the developing inflorescence of rice. Genome res.

[CR14] Jones-Rhoades MW, Bartel DP, Bartel B (2006). MicroRNAS and their regulatory roles in plants. Ann Rev Plant Biol.

[CR15] Khanday I, Yadav SR, Vijayraghavan U (2013). Rice *LHS1/OsMADS1* controls floret meristem specification by coordinated regulation of transcription factors and hormone signaling pathways. Plant Physiol.

[CR16] Koes R (2008). Evolution and development of virtual inflorescences. Trends Plant Sci.

[CR17] Komiya R, Ohyanagi H, Niihama M, Watanabe T, Nakano M, Kurata N, Nonomura K-I (2014). Rice germline-specific Argonaute MEL1 protein binds to phasiRNAs generated from more than 700 lincRNAs. Plant J.

[CR18] Kozomara A, Griffiths-Jones S (2011). miRBase: integrating microRNA annotation and deep-sequencing data. Nucl Acids Res.

[CR19] Kyozuka J, Tokunaga H, Yoshida A (2014). Control of grass inflorescence form by the fine-tuning of meristem phase change. Curr Opin Plant Biol.

[CR20] Li Y, Li C, Xia J, Jin Y (2011). Domestication of transposable elements into MicroRNA genes in plants. PLoS ONE.

[CR21] Li Z-M, Zheng X-M, Ge S (2011). Genetic diversity and domestication history of African rice (*Oryza glaberrima*) as inferred from multiple gene sequences. Theor Appl Genet.

[CR22] Li F, Pignatta D, Bendix C, Brunkard JO, Cohn MM, Tung J, Sun H, Kumar P, Baker B (2012). MicroRNA regulation of plant innate immune receptors. Proc. Natl. Acad. Sci. U.S.A..

[CR23] Linares OF (2002). African rice (*Oryza glaberrima*): history and future potential. Proc. Natl. Acad. Sci. U.S.A..

[CR24] Liu B, Chen Z, Song X, Liu C, Cui X, Zhao X, Fang J, Xu W, Zhang H, Wang X (2007). Oryza sativa *dicer-like4* reveals a key role for small interfering RNA silencing in plant development. Plant Cell.

[CR25] Liu Y, Wang Y, Zhu QH, Fan L (2013). Identification of phasiRNAs in wild rice (*Oryza rufipogon*). Plant Signal Behav.

[CR26] Martin M (2011). *Cutadapt* removes adapter sequences from high-throughput sequencing reads. EMBnetwork J.

[CR27] Moyroud E, Kusters E, Monniaux M, Koes R, Parcy F (2010). LEAFY blossoms. Trends Plant Sci.

[CR28] Nabholz B, Sarah G, Sabot F, Ruiz M, Adam H, Nidelet S, Ghesquière A, Santoni S, David J, Glémin S (2014). Transcriptome population genomics reveals severe bottleneck and domestication cost in the African rice (*Oryza glaberrima*). Mol Ecol.

[CR29] Nogueira FTS, Madi S, Chitwood DH, Juarez MT, Timmermans MCP (2007). Two small regulatory RNAs establish opposing fates of a developmental axis. Genes Dev.

[CR30] Nonomura K-I, Morohoshi A, Nakano M, Eiguchi M, Miyao A, Hirochika H, Kurata N (2007). A germ cell specific gene of the *ARGONAUTE* family is essential for the progression of premeiotic mitosis and meiosis during sporogenesis in rice. Plant Cell.

[CR31] Orjuela J, Sabot F, Chéron S, Vigouroux Y, Adam H, Chrestin H, Sanni K, Lorieux M, Ghesquière A (2014). An extensive analysis of the African rice genetic diversity through a global genotyping. Theor Appl Genet.

[CR32] Park SJ, Jiang K, Schatz MC, Lippman ZB (2012). Rate of meristem maturation determines inflorescence architecture in tomato. Proc. Natl. Acad. Sci. U.S.A..

[CR33] Park SJ, Eshed Y, Lippman ZB (2014). Meristem maturation and inflorescence architecture - lessons from the Solanaceae. Curr Opin Plant Biol.

[CR34] Peng T, Lv Q, Zhang J, Li J, Du Y, Zhao Q (2011). Differential expression of the microRNAs in superior and inferior spikelets in rice (*Oryza sativa*). J Exp Bot.

[CR35] Prusinkiewicz P, Erasmus Y, Lane B, Harder LD, Coen E (2007). Evolution and development of inflorescence architectures. Science.

[CR36] Rice P, Longden I, Bleasby A (2000). EMBOSS: The European Molecular Biology Open Software Suite. Trends Genet.

[CR37] Second G (1982). Origin of the genic diversity of cultivated rice (*Oryza* spp.): study of the polymorphism scored at 40 isozyme loci. Jap J Genet.

[CR38] Shivaprasad PV, Chen H-M, Patel K, Bond DM, Santos BACM, Baulcombe DC (2012). A microRNA superfamily regulates nucleotide binding site-leucine-rich repeats and other mRNAs. Plant Cell.

[CR39] Song X, Li P, Zhai J, Zhou M, Ma L, Liu B, Jeong D-H, Nakano M, Cao S, Liu C (2012). Roles of DCL4 and DCL3b in rice phased small RNA biogenesis. Plant J.

[CR40] Song X, Wang D, Ma L, Chen Z, Li P, Cui X, Liu C, Cao S, Chu C, Tao Y (2012). Rice RNA-dependent RNA polymerase 6 acts in small RNA biogenesis and spikelet development. Plant J.

[CR41] Souaze F, Ntodou-Thome A, Tran CY, Rostene W, Forgez P (1996). Quantitative RT-PCR: limits and accuracy. Biotechniques.

[CR42] Stocks MB, Moxon S, Mapleson D, Woolfenden HC, Mohorianu I, Folkes L, Schwach F, Dalmay T, Moulton V (2012). The UEA sRNA workbench: a suite of tools for analyzing and visualizing next generation sequencing microRNA and small RNA datasets. Bioinformatics.

[CR43] Varkonyi-Gasic E, Wu R, Wood M, Walton EF, Hellens RP (2007). Protocol: a highly sensitive RT-PCR method for detection and quantification of microRNAs. Plant Meth.

[CR44] Vaughan DA, Lu BR, Tomooka N (2008). The evolving story of rice evolution. Plant Sci.

[CR45] Vogel JP, Garvin DF, Mockler TC, Schmutz J, Rokhsar D, Bevan MW, Barry K, Lucas S, Harmon-Smith M, Lail K (2010). Genome sequencing and analysis of the model grass Brachypodium distachyon. Nature.

[CR46] Wang Y, Shen D, Bo S, Chen H, Zheng J, Zhu Q-H, Cai D, Helliwell C, Fan L (2010). Sequence variation and selection of small RNAs in domesticated rice. BMC Evol Biol.

[CR47] Wang Y, Bai X, Yan C, Gui Y, Wei X, Zhu Q-H, Guo L, Fan L (2012). Genomic dissection of small RNAs in wild rice (*Oryza rufipogon*): lessons for rice domestication. New Phytol.

[CR48] Wang M, Yu Y, Haberer G, Marri PR, Fan C, Goicoechea JL, Zuccolo A, Song X, Kudrna D, Ammiraju JSS (2014). The genome sequence of African rice (*Oryza glaberrima*) and evidence for independent domestication. Nature Genet.

[CR49] Wei L, Yan L, Wang T (2011). Deep sequencing on genome-wide scale reveals the unique composition and expression patterns of microRNAs in developing pollen of *Oryza sativa*. Genome Biol.

[CR50] Xia R, Meyers BC, Liu Z, Beers EP, Ye S, Liu Z (2013). MicroRNA superfamilies descended from *miR390* and their roles in secondary small interfering RNA biogenesis in Eudicots. Plant Cell.

[CR51] Yan Y, Zhang Y, Yang K, Sun Z, Fu Y, Chen X (2011). Small RNAs from MITE-derived stem-loop precursors regulate abscisic acid signaling and abiotic stress responses in rice. Plant J.

[CR52] Zhai J, Jeong D-H, De Paoli E, Park S, Rosen BD, Li Y, González AJ, Yan Z, Kitto SL, Grusak MA (2011). MicroRNAs as master regulators of the plant NB-LRR defense gene family via the production of phased, trans-acting siRNAs. Genes Dev.

[CR53] Zhai J, Zhang H, Arikit S, Huang K, Nan G-L, Walbot V, Meyers BC (2015). Spatiotemporally dynamic, cell-type-dependent premeiotic and meiotic phasiRNAs in maize anthers. Proc. Natl. Acad. Sci. U.S.A..

[CR54] Zhang D, Yuan Z (2014). Molecular control of grass inflorescence development. Ann Rev Plant Biol.

[CR55] Zhang Q-J, Zhu T, Xia E-H, Shi C, Liu Y-L, Zhang Y, Liu Y, Jiang W-K, Zhao Y-J, Mao S-Y (2014). Rapid diversification of five Oryza AA genomes associated with rice adaptation. Proc. Natl. Acad. Sci. U.S.A..

[CR56] Zhang Y-C, Liao J-Y, Li Z-Y, Yu Y, Zhang J-P, Li Q-F, Qu L-H, Shu W-S, Chen Y-Q (2014). Genome-wide screening and functional analysis identify a large number of long noncoding RNAs involved in the sexual reproduction of rice. Genome Biol.

[CR57] Zheng Y, Wang Y, Wu J, Ding B, Fei Z (2015). A dynamic evolutionary and functional landscape of plant phased small interfering RNAs. BMC Biol..

