# Supplementary material for: miR2118-triggered phased siRNAs are differentially expressed during the panicle development of wild and domesticated African rice species
Source: Rice (N Y). 2016 Mar 12;9:10. doi: 10.1186/s12284-016-0082-9 (PMC4788661; doi:10.1186/s12284-016-0082-9)
Supplement: Additional file 1: Table S1. — List of genotypes used in this study. Table S2. Summary statistics of small RNA libraries. Table S3. Summary of sequence number (clusters and reads) mapped to O. sativa Nipponbare reference genome (MSU7.0) and O. glaberrima CG14 reference genome for O. barthii and O. glaberrima small RNA sequences. Table S4. Summary of sequence number (clusters and reads) in each annotation class (i.e. sequence databases) for O. barthii and O. glaberrima small RNA sequences based on BLAST-filtering. Table S5. List of primers used in this study. The bases in brackets represent the ones that were modified LNAs. The sequences in italic correspond to stem-loop region for stem-loop RT-PCRs. The underline sequences correspond to T7 RNA polymerase binding site used for RNA probe synthesis. (PDF 191 kb) [file 12284_2016_82_MOESM1_ESM.pdf]

Additional file 1 Table S1. Lists of genotypes used in this study

Species	Accession nb	Origin
O. barthii	B197	Lake Tchad
O. barthii	B117	Botswana
O. barthii	B88	Cameroun
O. barthii	B74	Lake Tchad
O. barthii	B70	Lake Tchad
O. barthii	B68	Lake Tchad
O. barthii	B64	Nigeria
O. barthii	B48	Mali
O. barthii	B5	Lake Tchad
O. barthii	B49	Lake Tchad
O. glaberrima	Tog 6208	Guinea
O. glaberrima	Tog6211	Nigeria
O. glaberrima	AC104589	Burkina Faso
O. glaberrima	MG12	Mali
O. glaberrima	CG14	Senegal
O. glaberrima	Tog 5681	Nigeria
O. glaberrima	Tog 7020	Sierra Leon
O. glaberrima	Tog 6221	Burkina Faso
O. glaberrima	Tog 5887	Liberia
O. glaberrima	Tog 5500	Nigeria

Additional file 1

Table S2. Summary statistics of small RNA libraries

	O. barthii	***O. glaberrima***
**Raw data**	71.4 x10^6 reads	73.3 x10^6 reads
**High quality clustered data**	33.9 x10^6 reads	33.1 x10^6 reads
	9.5 x10^5 clusters	9.0 x10^5 clusters
**Annotated data**	26.3 x10^6 reads	26.0 x10^6 reads
	6.4 x10^5 clusters	6.2 x10^5 clusters

Additional file 1 Table S3. Summary of sequence number (clusters and reads) mapped to *O. sativa* Nipponbare reference genome (MSU7.0) and *O. glaberrima* CG14 reference genome for *O. barthii* and *O. glaberrima* small RNA sequences

	O. barthii			*O. glaberrima*		
	Mapped	Unmapped	All	Mapped	Unmapped	All
Clusters_O sativa Ref	602783	342602	945385	581755	320141	901896
Clusters_O glaberrima Ref	765371	180014	945385	755833	146063	901896
Reads_O sativa Ref	24464558	9458157	33922715	23882801	9221817	33104618
Reads_O glaberrima Ref	20885463	13037252	33922715	18617611	14487007	33104618

Additional file 1

Table S4. Summary of sequence number (clusters and reads) in each annotation class (i.e. sequence databases) for *O. barthii* and *O. glaberrima* small RNA sequences based on BLAST-filtering.

	Clusters		Reads	
Databases	*O. barthii*	*O. glaberrima*	*O. barthii*	*O. glaberrima*
mirBase	39297	38225	3203043	3025155
Rfam	52053	51204	8203693	9948614
Repeats	198813	194216	7293624	7173969
CDS (MSU7.0)	46595	45024	1033340	877832
Intron-UTR (MSU7.0)	57354	55231	1265556	1004540
O. sativa (MSU7.0)	248673	236065	5263198	3873068

Additional file 1

Table S5. List of primers used in this study. The bases in brackets represent the ones that were modified LNAs. The sequences in italic correspond to stem-loop region for stem-loop RT-PCRs. The underline sequences correspond to T7 RNA polymerase binding site used for RNA probe synthesis.

	**Name**	**Sequence**
**Northern-blotting**	miR2118f	TAGGAATGGGAGGCATCAGGAA
	U6	GCAGGGGCCATGCTAATCTTCTCTGTATCGT
**Stem-loop qRT-PCR**	miR2118-RT	*GTCGTATCCAGTGCAGGGTCCGAGGTATTCGCACTGGATACGAC*TAGGAA
	miR2118f-F	CGGCGGTTCCTAATGCCTCCCA
	miR159b-RT	*GTCGTATCCAGTGCAGGGTCCGAGGTATTCGCACTGGATACGAC*CAGAGC
	miR159-F	CGGCGGTTTGGATTGAAGGGA
	Univ-RT	GTGCAGGGTCCGAGGT
	PH12-RT	*GTCGTATCCAGTGCAGGGTCCGAGGTATTCGCACTGGATACGAC*CCCTTT
	PH12-F	CGGCGGCGAGCTGTTAACCAG
	PH557-RT	*GTCGTATCCAGTGCAGGGTCCGAGGTATTCGCACTGGATACGAC*ATGCCG
	PH557-F	CGGCGGCGTTCTGATGATTTG
	PH612-RT	*GTCGTATCCAGTGCAGGGTCCGAGGTATTCGCACTGGATACGAC*CCGTTC
	PH612-F	CGGCGGCCAGCAGGACTTGGG
	PH779-RT	*GTCGTATCCAGTGCAGGGTCCGAGGTATTCGCACTGGATACGAC*TCATCT
	PH779-F	CGGCGGTCATGAATCAGCAGT
**Classic qRT-PCR**	PH12-F2	TCTCGATCGTTGATGCCATA

PH12-R1	GGAGTCGTTGGTTCCTTCAA
PH557-F2	TCTGATGATTTGCGGCATAG
PH557-R1	GGGGTTTTATGGTTGCAGAA
PH612-F2	GAACCAGCCATCAGAAAGCT
PH612-R1	AAGTGTCCAGCAGGACTTGG
PH779-F2	TTGTCCACAATAGGGCATCA
PH779-R1	ACCTCAAATCCAGCGTTCAT
ACT-F	CATTCCAGCAGATGTGGATTG
ACT-R	TCTTGGCTTAGCATTCTTGG
OSH1_F	CAGTTCGTGATGATGGAC
OSH1_R	CTAAAACCGACCCCTGCATTA
APO1-F	GTTCTACTGCATGAGCTCGTC
APO1-R	TGCACCTTGCTCCATACGTTC
APO2-F	AGGTGCAATCCATGGCTAAG
APO2-R	GCATCTTGGGCTTGTTGATG
LHS1-F	GTGACCATTCCCTGCAGATT
LHS1-R	GTCTGCTGCTTCATTGCTCA
SPL14-F	CTGCCTGAATTTGACCAAGG
SPL14-R	AAGCTTCTGAACCTGCGATG
LAX1-F	ATTACCGGTTGGTCATGGTC
LAX1-R	AAGCGATCGAGCAAACAAGT
OsDCL4-F	TCAGAAGAAGGCTGCACAAG
OsDCL4-R	CGAACGTCCTCTTCTTTTGG

	OsMEL1-F	TCCCAAGATCAAGGAGAACG
	OsMEL1-R	ACAAGCAACCAGCTCCAAAC
***In situ* hybridizations**	LNA-miR2118f	TAGG(A)ATGGG(A)GGC(A)TC(A)GGAA
	LNA-PH12	CCC(T)TTC(T)GGT(T)AAC(A)GCTCG
	LHS1-HIS-F	GAAGAGCAAGGAGCAACAGC
	LHS1-HIS-R	AATCTGCAGGGAATGGTCAC
	LHS1-HIS-T7R	GCGAAATTAATACGACTCACTATAGGGCGAAAATCTGCAGGGAATGGTCAC
	PH12_T7R	GCGAAATTAATACGACTCACTATAGGGCGAAGGAGTCGTTGGTTCCTTCAA
