# Supplementary material for: miR2118-triggered phased siRNAs are differentially expressed during the panicle development of wild and domesticated African rice species
Source: Rice (N Y). 2016 Mar 12;9:10. doi: 10.1186/s12284-016-0082-9 (PMC4788661; doi:10.1186/s12284-016-0082-9)
Supplement: Additional file 2: — Size distribution, mapping and annotation of O. barthii and O. glaberrima panicle-derived small RNAs on O. sativa Nipponbare genome. (a) Small RNA size distribution of clusters (distinct) and reads (abundance). The size of small RNAs was plotted versus frequency (percentage relative to total abundance). (b) Mapping rate (percentage relative to total number) of clusters (i.e. distinct sequences) and total reads on O. sativa nipponbare MSU7.0. The percentage values are indicated in the bars. (c) Small RNA annotation frequency (percentage relative to total abundance) of clusters (distinct) and individual reads (abundance). The percentage values are indicated in the bars. (d) Size distribution of the annotated small RNA clusters (i.e. distinct sequences) according the annotation classes. (e) Size distribution of the annotated small RNA sequences (i.e. abundance/reads) according the annotation classes. (PDF 333 kb) [file 12284_2016_82_MOESM2_ESM.pdf]

Additional file 3. Size distribution, mapping and annotation of *O. barthii* and *O. glaberrima* panicle-derived small RNAs on *O. sativa Nipponbare* genome.

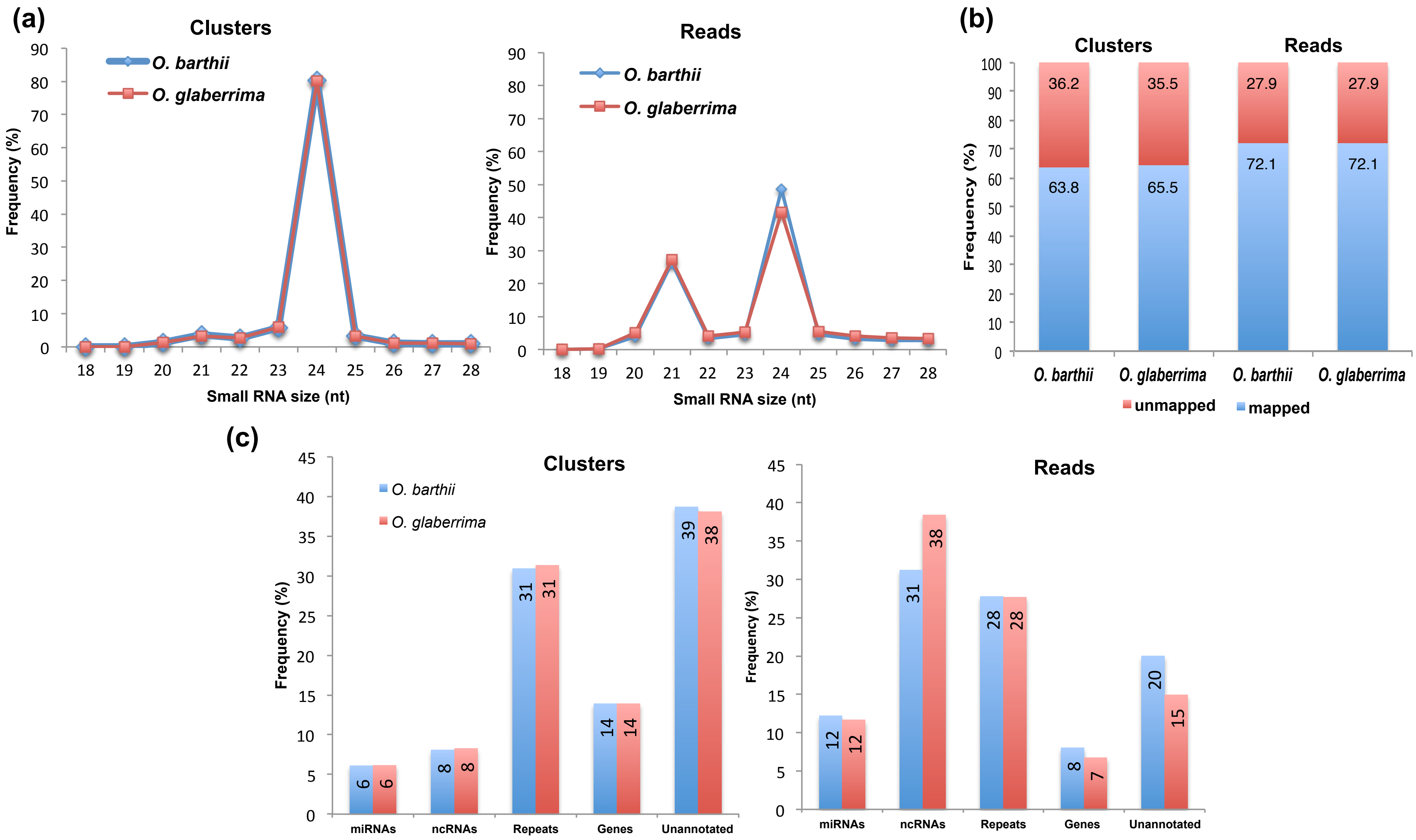

(d)

Annotated Cluster distribution

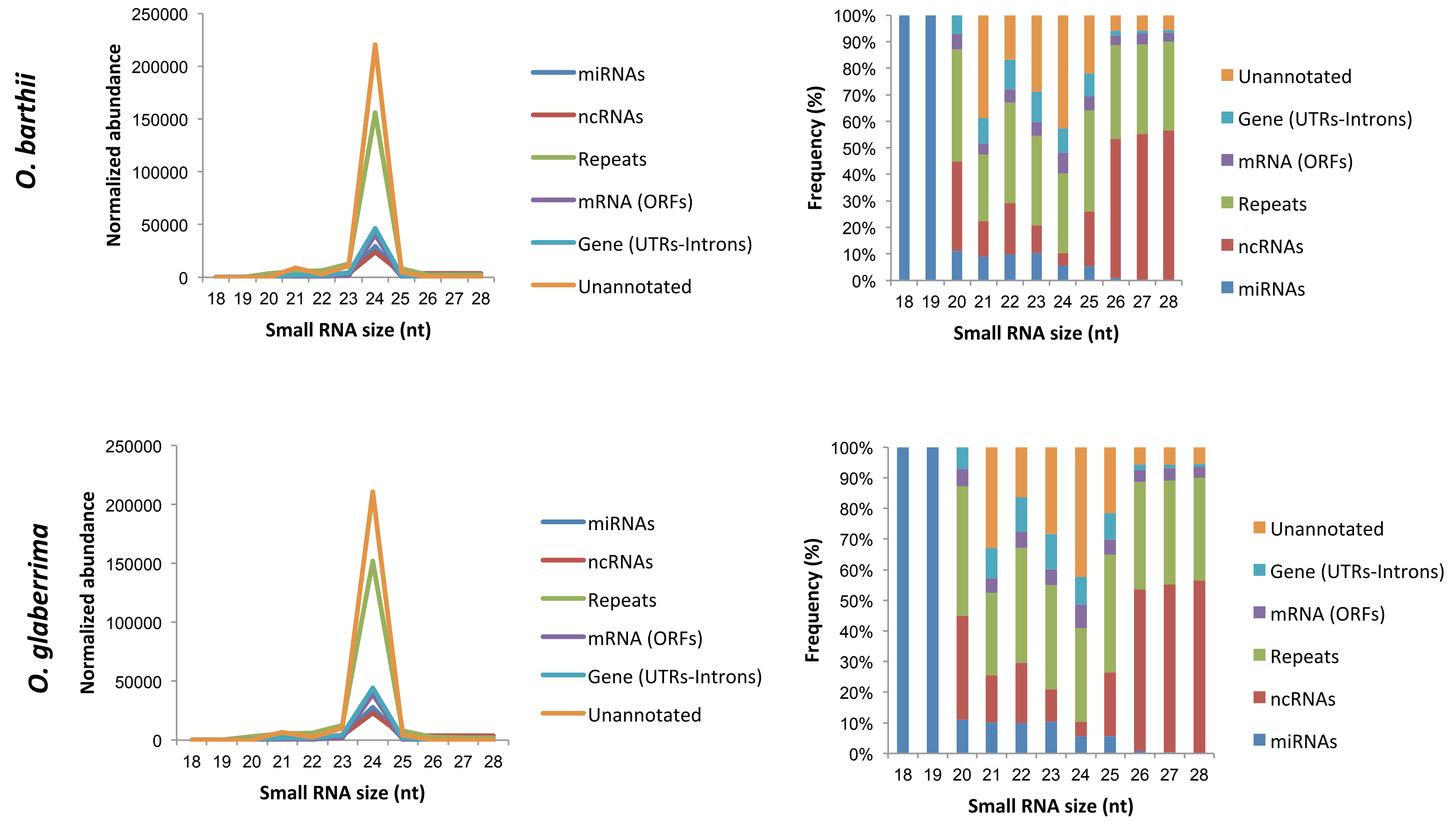

(e)

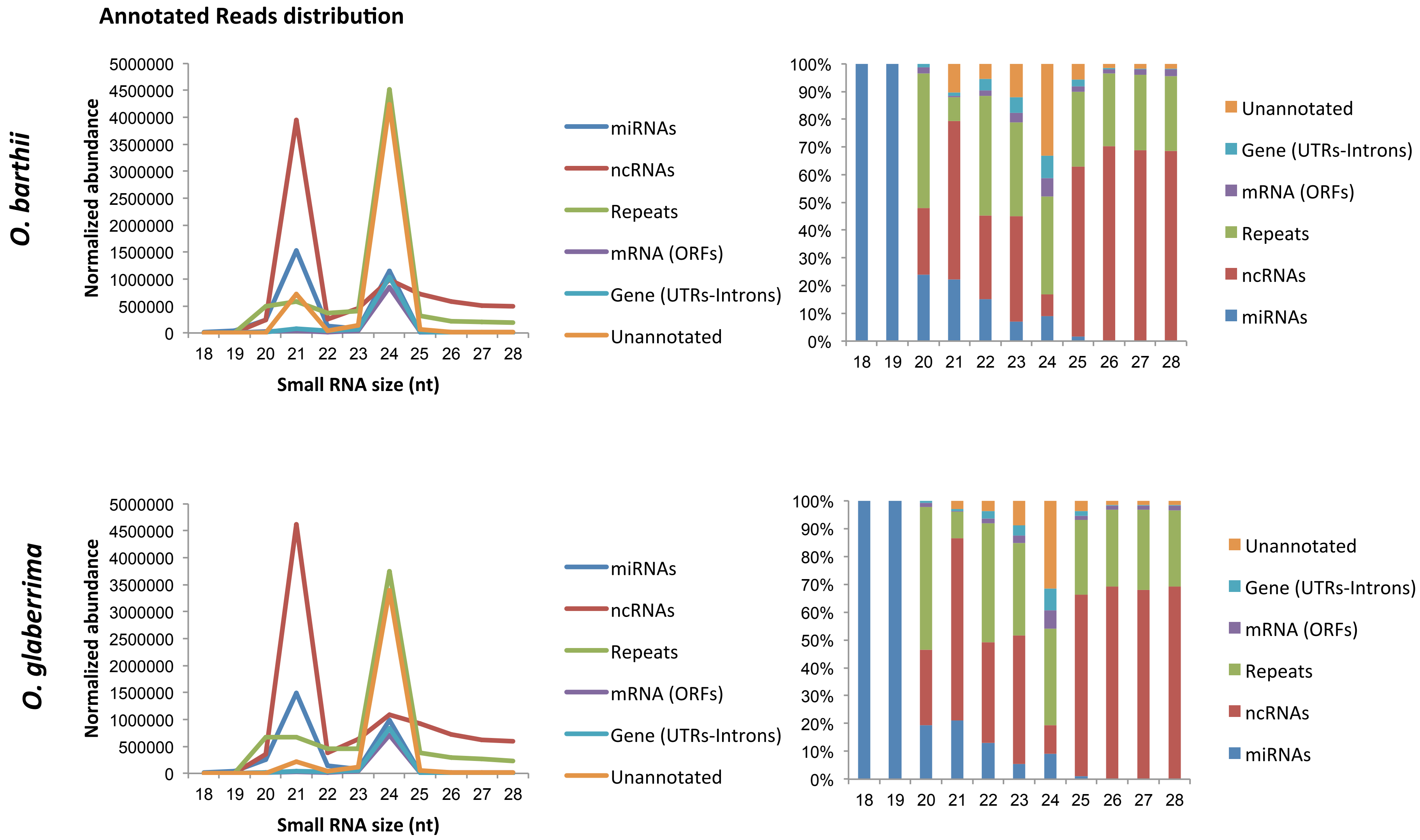

Annotated Reads distribution
O. barthii
O. glaberrima
Normalized abundance
Small RNA size (nt)
miRNAs
ncRNAs
Repeats
mRNA (ORFs)
Gene (UTRs-Introns)
Unannotated
