# Supplementary material for: miR2118-triggered phased siRNAs are differentially expressed during the panicle development of wild and domesticated African rice species
Source: Rice (N Y). 2016 Mar 12;9:10. doi: 10.1186/s12284-016-0082-9 (PMC4788661; doi:10.1186/s12284-016-0082-9)
Supplement: Additional file 3: — Genomic distribution and abundance of O. barthii and O. glaberrima panicle-derived small RNAs on O. sativa nipponbare genome. (a) Distribution and abundance of O. barthii reads vs. O. sativa nipponbare genome MSU7.0. (b) Distribution and abundance of O. glaberrima reads vs. O. sativa nipponbare genome MSU7.0. (PDF 1091 kb) [file 12284_2016_82_MOESM3_ESM.pdf]

(a)

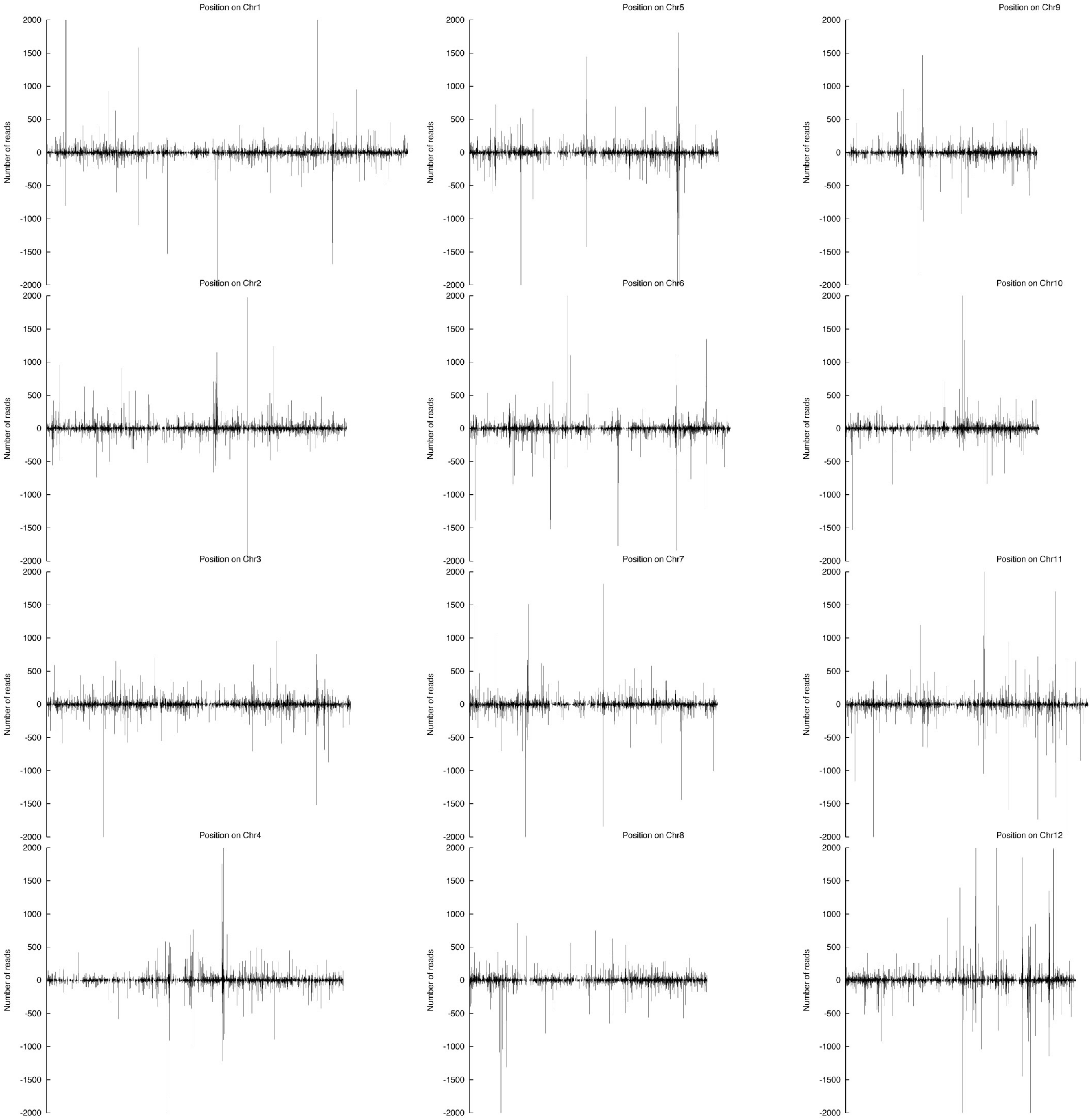

Additional file 3. Genomic distribution and abundance of *O. barthii* and *O. glaberrima* panicle-derived small RNAs on *O. sativa nipponbare* genome.

(b)

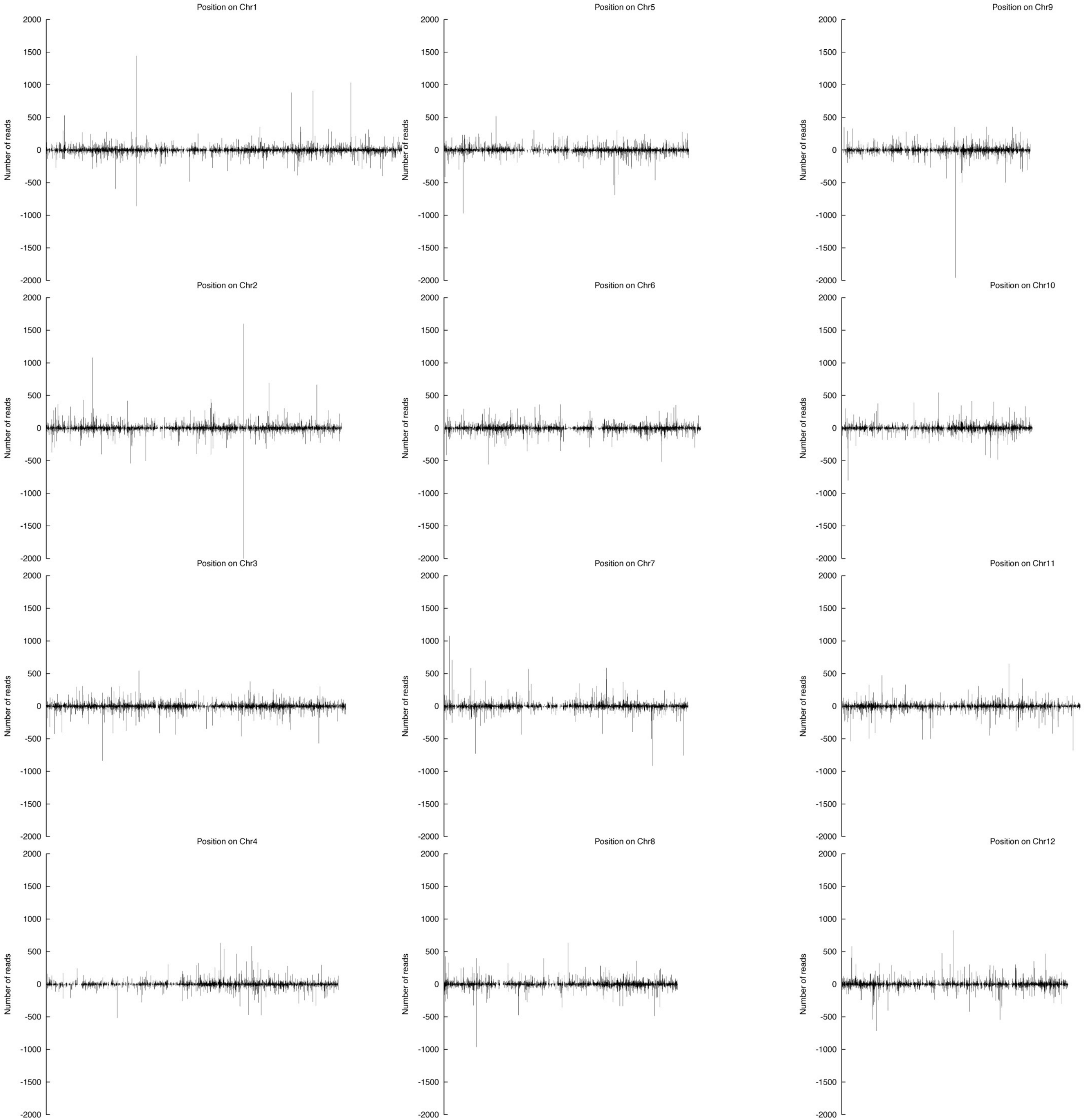
Position on Chr1
Position on Chr2
Position on Chr3
Position on Chr4
Position on Chr5
Position on Chr6
Position on Chr7
Position on Chr8
Position on Chr9
Position on Chr10
Position on Chr11
Position on Chr12
Number of reads
2000
1500
1000
500
0
-500
-1000
-1500
-2000
