# Supplementary figures and images for: miR2118-triggered phased siRNAs are differentially expressed during the panicle development of wild and domesticated African rice species
Source: Rice (N Y). 2016 Mar 12;9:10. doi: 10.1186/s12284-016-0082-9 (PMC4788661; doi:10.1186/s12284-016-0082-9)

Additional file 4. Bio-informatic workflow for small RNA analysis.

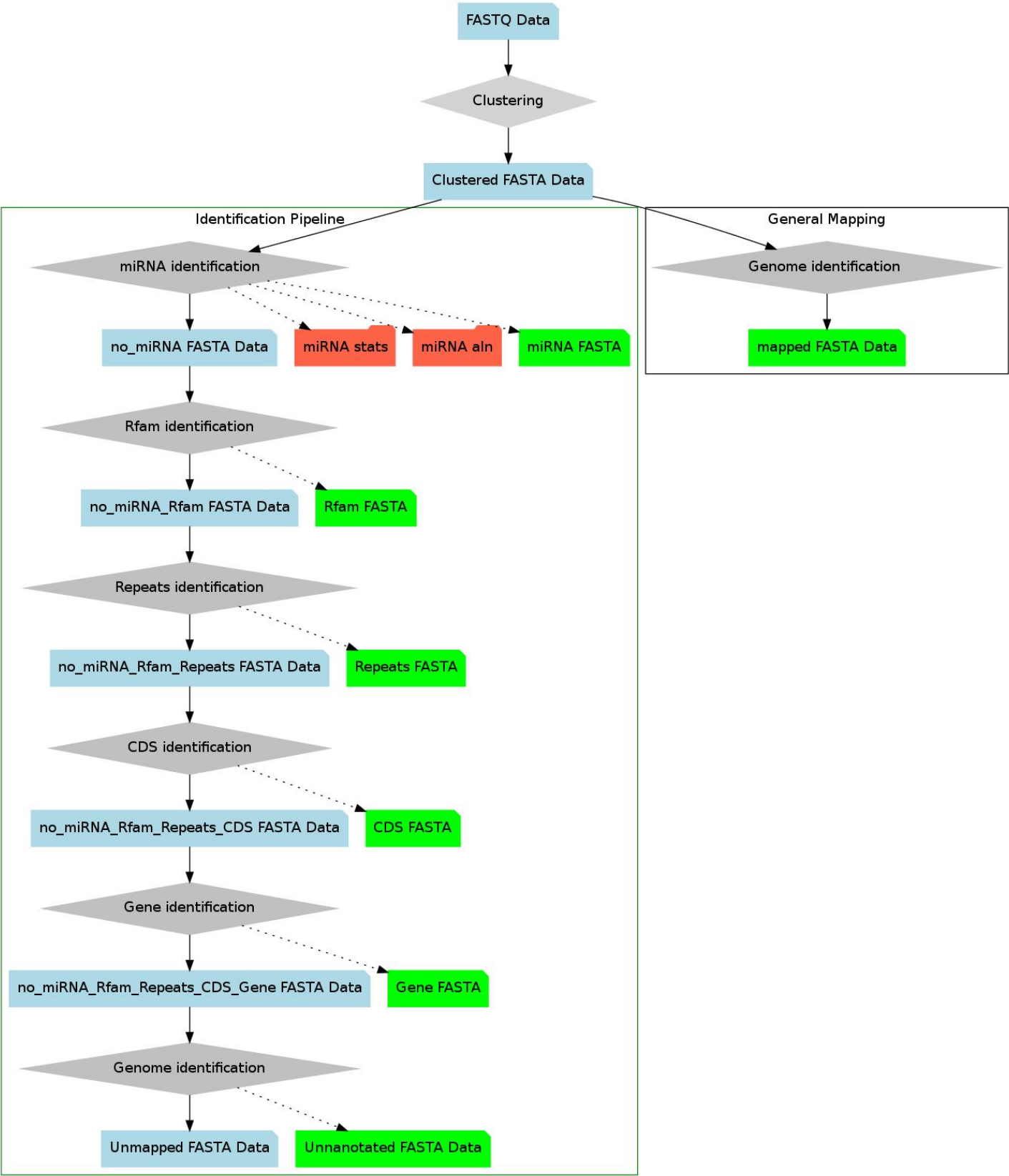

Supplement: Additional file 4: — Bio-informatic workflow for small RNA analysis. (PDF 158 kb) [file 12284_2016_82_MOESM4_ESM.pdf]

**Additional file 5. Relative abundance of small RNAs  
between *O. barthii* and *O. glaberrima*.**

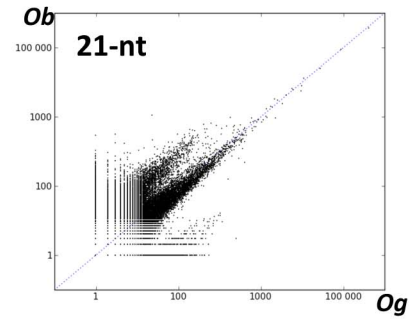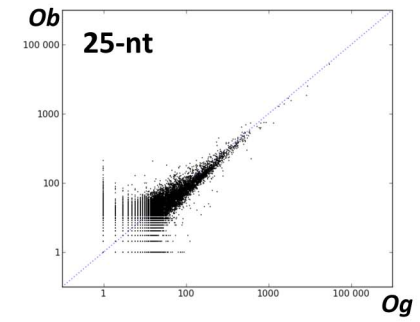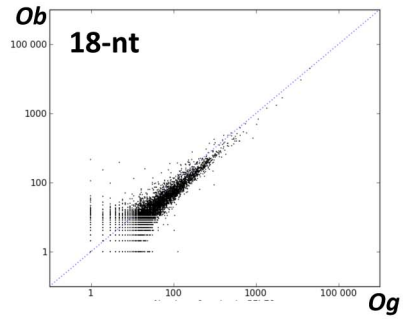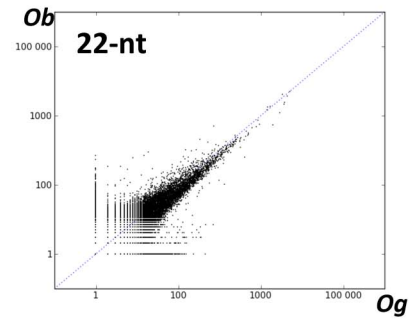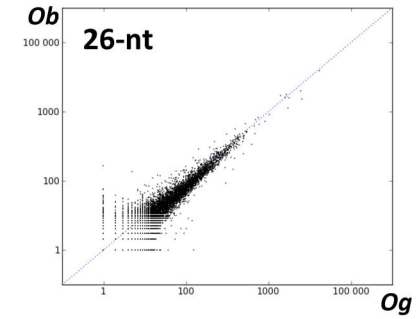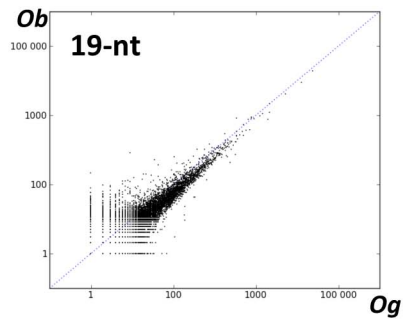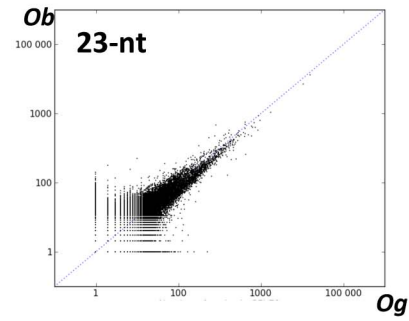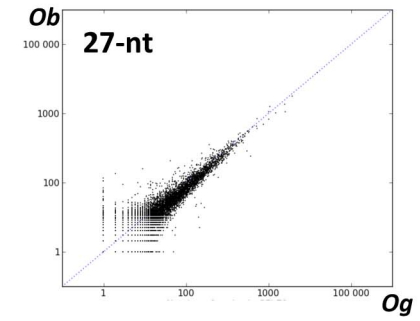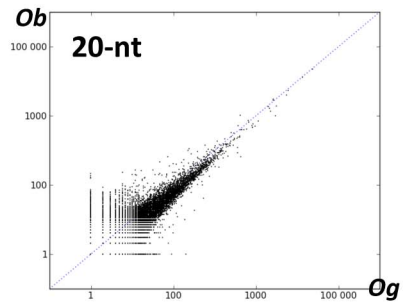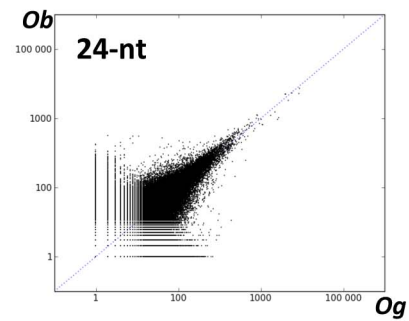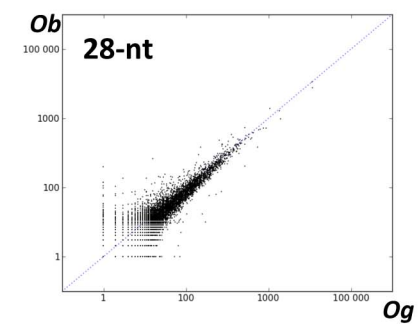

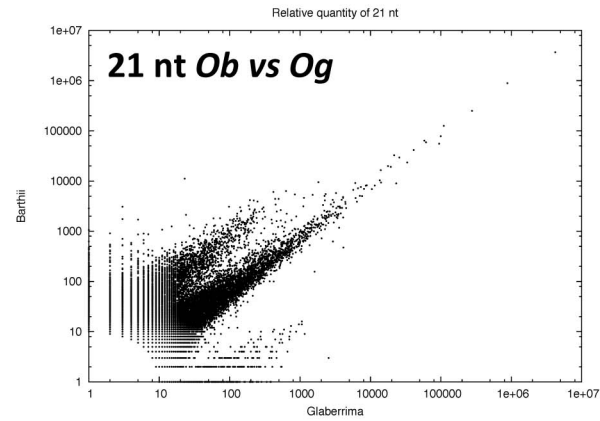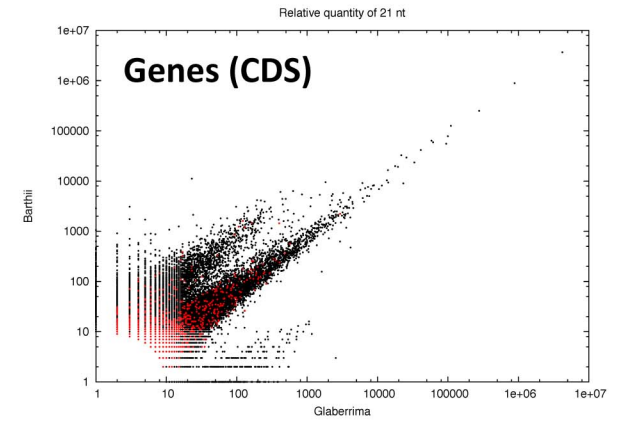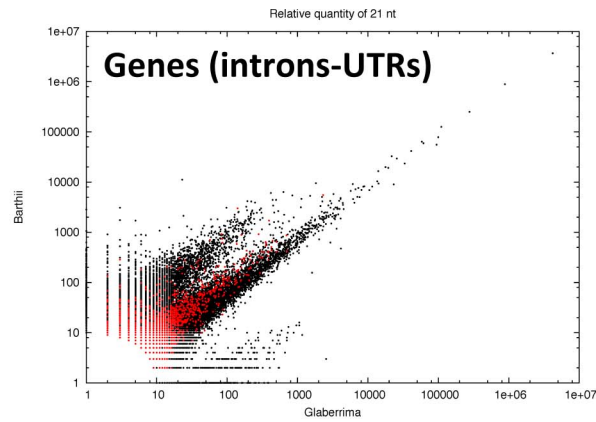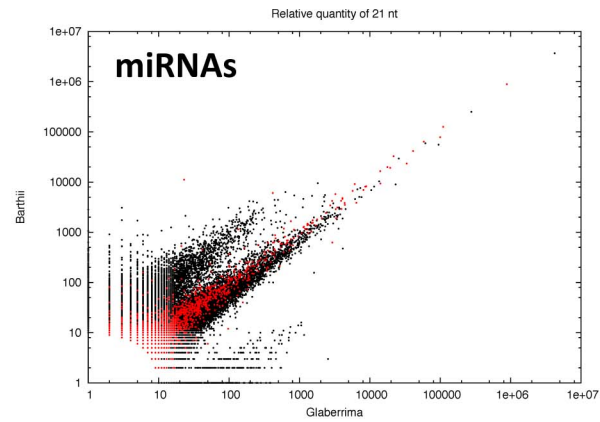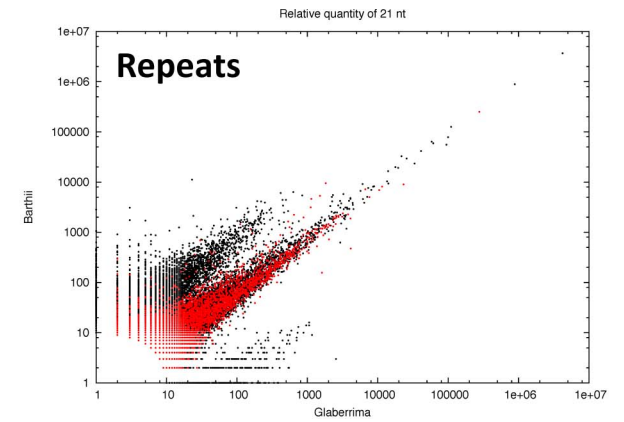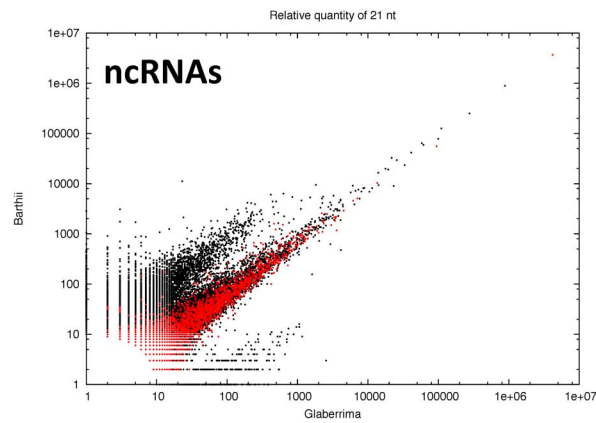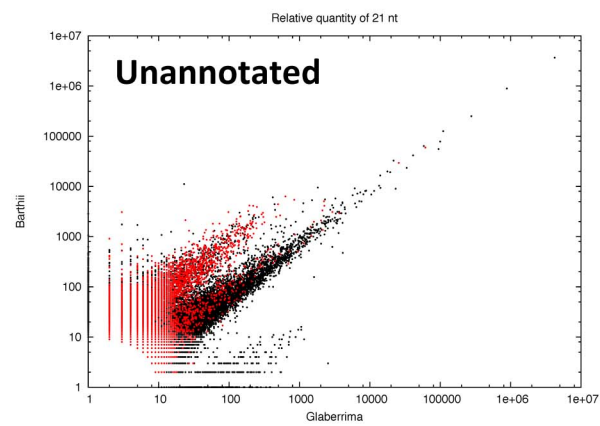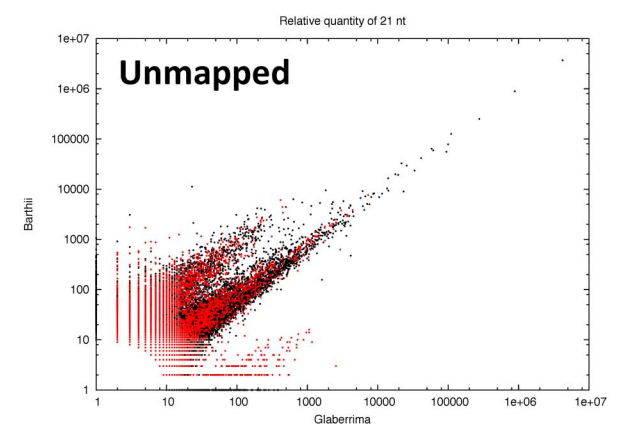

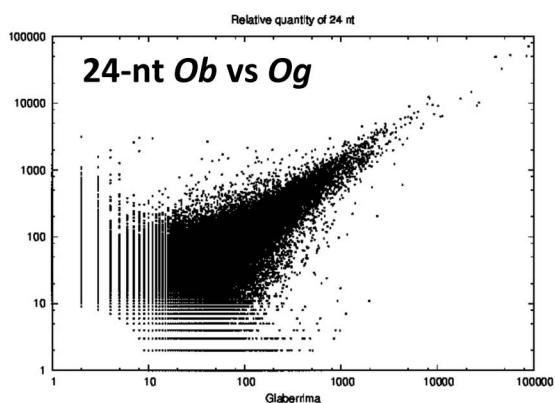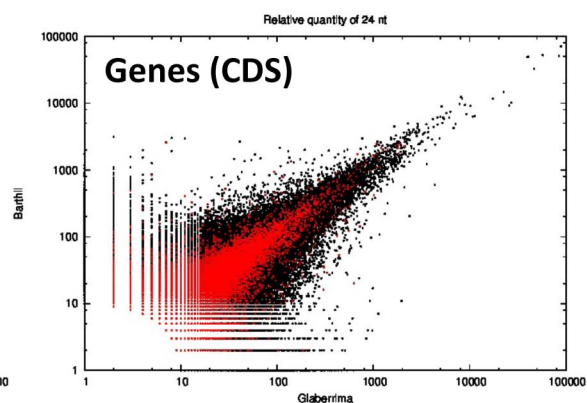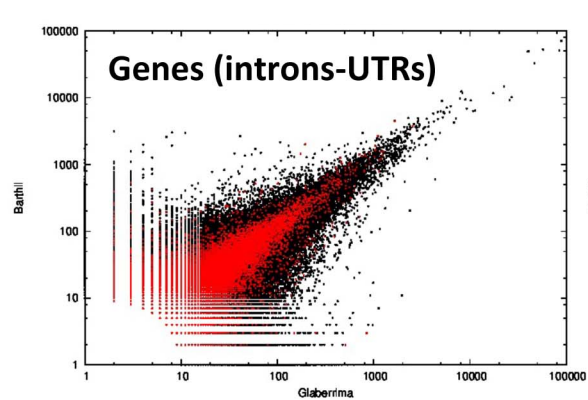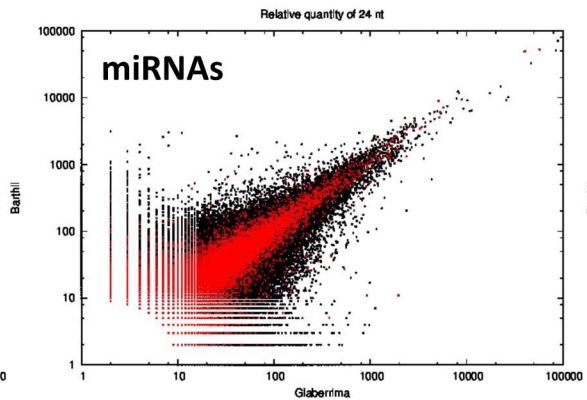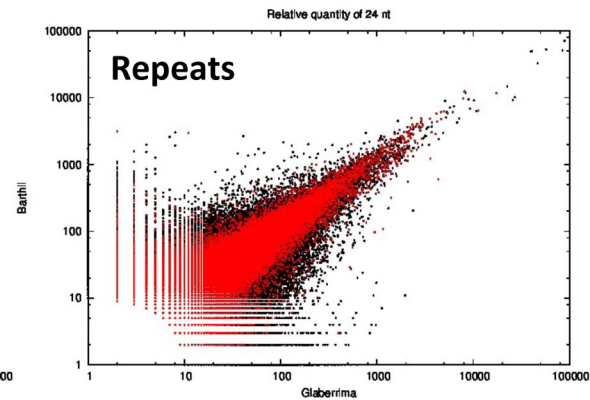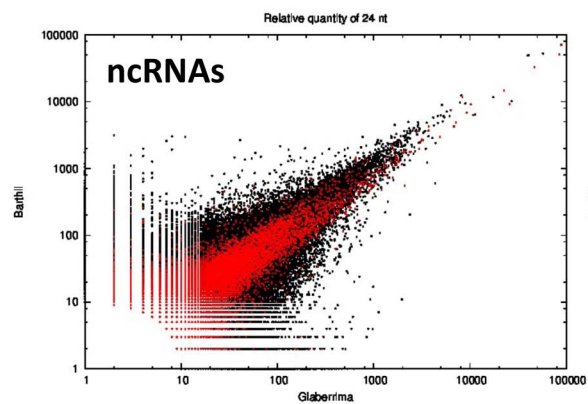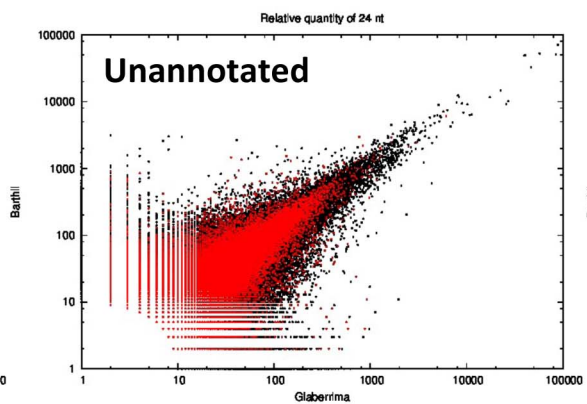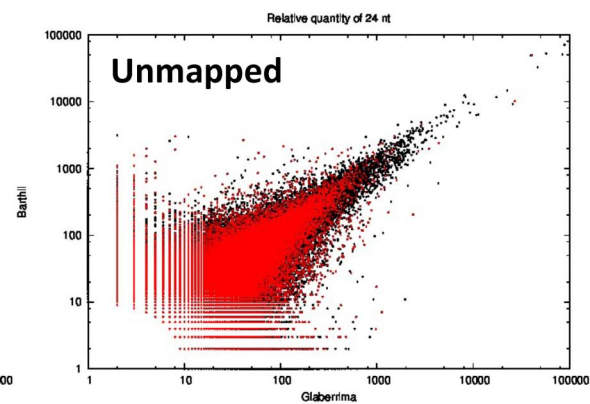

Supplement: Additional file 5: — Relative abundance of small RNAs between O. barthii and O. glaberrima. (a) Relative abundance of small RNAs between O. barthii (Ob) and O. glaberrima (Og) according to their size (from 18- to 28-nt small RNAs). LogPlots of normalized abundance of distinct small RNA sequences. (b) Relative abundance of the annotation classes of 21-nt small RNAs between O. barthii (Ob) and O. glaberrima (Og). LogPlot of normalized abundance of distinct small RNA sequences. Black dots represent global 21-nt small RNAs, and red dots the class of annotated 21-nt small RNAs. (c) Relative abundance of the annotation classes of 24-nt small RNAs between O. barthii (Ob) and O. glaberrima (Og). LogPlot of normalized abundance of distinct small RNA sequences. Black dots represent whole 24-nt small RNAs, red dots represent the class of annotated 24-nt small RNAs. (PDF 1571 kb) [file 12284_2016_82_MOESM5_ESM.pdf]
