# Supplementary material for: miR2118-triggered phased siRNAs are differentially expressed during the panicle development of wild and domesticated African rice species
Source: Rice (N Y). 2016 Mar 12;9:10. doi: 10.1186/s12284-016-0082-9 (PMC4788661; doi:10.1186/s12284-016-0082-9)
Supplement: Additional file 6: — 21-nt small RNAs associated to phased loci and mature miRNA families detected in young panicles of O. glaberrima and O. barthii. (a) O. sativa Nipponbare loci associated with 21-nt phased and unphased siRNAs from O. barthii and O. glaberrima detected using sRNAworkbench facilities. (b) Normalized count of reads (relative abundance for 2 millions of total reads) associated with mature miRNA families in relation to small RNA size. (PDF 383 kb) [file 12284_2016_82_MOESM6_ESM.pdf]

Additional file 6: 21-nt small RNAs associated to phased loci and mature miRNA families detected in young panicles of *O. glaberrima* and *O. barthii*.

(a) *O. sativa* Nipponbare loci associated with 21-nt phased and unphased siRNAs from *O. barthii* and *O. glaberrima* detected using sRNAworkbench facilities. Alt_name: corresponding locus name in Song et al (2012a); Chr: chromosome number; Positions (sRNAworkbench): position of the detected locus on chromosome (*O. sativa* reference genome MSU 7.0); Dist_w/previous (bp): distance from previous locus in bp; MEME motif position (bp): position of the MEME motif from the 5' end of the detected locus; Size_w/MEME (bp): length of the detected locus from the MEME motif (default=251 bp without MEME motif); Phased clusters nb (Ob_Og): number of associated phasiRNAs in *O. barthii* and *O. glaberrima* respectively; Unphased_clusters nb (Ob_Og): number of associated non-phased small RNAs in *O. barthii* and *O. glaberrima* respectively; Common clusters nb: number of common phasiRNA sequences between *O. barthii* and *O. glaberrima*; Diff exp cluster nb: number of phasiRNA sequences differentially expressed between *O. barthii* and *O. glaberrima*; Total_reads nb (Ob_Og): total number of small RNA reads in *O. barthii* and *O. glaberrima* respectively; Total_phased_reads (Ob_Og): total number of phasiRNA reads in *O. barthii* and *O. glaberrima* respectively; Total_unphased_reads (Ob_Og): total number of non phased small RNA reads in O. barthii and O. glaberrima respectively; Shared cluster nb: name of phased loci sharing common phasiRNA sequences (numbers indicated in brackets); Annotation_Pipeline: annotation out-put of phasiRNA sequences for the corresponding phased locus from our customized annotation pipe-line (NA: no annotation); Annotated cluster nb: number of phasiRNA sequences annotated for the corresponding phased locus.

Name	Alt_name	Chr	Positions (sRNAworkbench)	Dist_w/previous (bp)	MEME motif position (bp)	Size_w/MEME (bp)	Phased clusters nb (Ob_Og)	Unphased_clusters nb (Ob_Og)	Common clusters nb	Diff exp cluster nb	Total_reads nb (Ob_Og)	Total_phased_reads (Ob_Og)	Total_unphased_reads (Ob_Og)	Annotation_Pipeline	Annotated cluster nb
PH-10	-	Chr01	2238876-2239127	..	-53	304	4_2	1_1	3	4	104_17	64_13	40_4	NA	0
PH-11	-	Chr01	2239699-2239950	572	-52	303	7_7	No	7	5	468_74	468_74	0_0	NA	0
PH-12	-	Chr01	2244238-2244489	4288	-95	346	11_9	No	9	10	4089_788	4089_788	0_0	NA	0
PH-13	phasi_NO7	Chr01	2313038-2313289	68549	ND	251	5_0	No	0	5	179_0	179_0	0_0	NA	0
PH-14	phasi_NO18	Chr01	2334980-2335231	21691	-158	409	4_2	No	2	4	2292_130	2292_130	0_0	NA	0
PH-15	phasi_NO19	Chr01	2342235-2342486	7004	ND	251	3_1	No	1	3	56_3	56_3	0_0	NA	0
PH-16	phasi_NO20	Chr01	2345887-2346138	3401	-32	283	9_4	No	4	9	264_14	264_14	0_0	NA	0
PH-17	phasi_NO21	Chr01	2347234-2347485	1096	-95	346	4_1	No	1	4	92_2	92_2	0_0	NA	0
PH-18	phasi_NO22	Chr01	2348046-2348297	561	-116	367	4_3	No	3	3	176_6	176_6	0_0	NA	0
PH-19	-	Chr01	2349178-2349429	881	-53	304	7_5	2_2	7	4	173_32	146_20	27_12	osa-MIR2118	1
PH-30	-	Chr01	4429716-4429967	2080287	ND	251	2_2	1_1	3	0	29_33	21_27	8_6	Rep_LOC_Os12g31040,Rep_LOC_	3
PH-31	-	Chr01	6081577-6081828	1651610	ND	251	1_1	No	1	0	22_6	22_6	0_0	Rep_LOC_Os12g33500	1
PH-32	-	Chr01	8214275-8214526	2132447	ND	251	2_2	1_1	3	0	25_43	17_37	8_6	Rep_LOC_Os12g31040,Rep_LOC_	3
PH-1	-	Chr01	10960748-10960999	2746222	ND	251	14_14	77_77	91	4	6522_6520	4364_4191	2158_2329	NA	0
PH-2	-	Chr01	12990707-12990958	2029708	ND	251	4_3	2_2	5	4	811_46	0_0	811_46	Rep_RN_42-23	6
PH-3	-	Chr01	12996023-12996274	5065	ND	251	4_3	2_2	5	4	811_46	0_0	811_46	Rep_RN_42-23	6
PH-4	-	Chr01	13253800-13254051	257526	ND	251	2_2	1_1	3	0	27_33	19_27	8_6	Rep_ORSgTEMT00500708,Rep_LO	3
PH-5	-	Chr01	19132002-19132253	5877951	ND	251	2_2	1_1	3	0	25_43	17_37	8_6	Rep_LOC_Os12g31040,Rep_LOC_	3
PH-6	phasi_NO31	Chr01	19145059-19145310	12806	ND	251	4_4	4_4	8	7	848_182	366_73	482_109	Rep_LOC_Os01g34710	8
PH-7	-	Chr01	20090869-20091120	945559	ND	251	3_0	No	0	3	68_0	68_0	0_0	NA	0
PH-8	phasi_NO40	Chr01	20452859-20453110	361739	ND	251	5_0	1_0	0	6	174_0	161_0	13_0	NA	0
PH-9	phasi_NO42	Chr01	20457869-20458120	4759	ND	251	3_2	No	2	3	2178_26	2178_26	0_0	NA	0
PH-20	phasi_NO50	Chr01	34219202-34219453	13761082	-32	283	5_4	No	4	4	458_40	458_40	0_0	NA	0
PH-21	-	Chr01	34222239-34222490	2786	-32	283	8_5	No	5	7	1933_117	1933_117	0_0	NA	0
PH-22	-	Chr01	34227269-34227520	4779	-11	262	10_10	1_1	11	6	1291_275	1208_251	83_24	NA	0
PH-23	phasi_NO54	Chr01	34229064-34229315	1544	-32	283	9_9	No	9	5	600_181	600_181	0_0	NA	0
PH-24	-	Chr01	34254108-34254359	24793	241	492	9_7	2_0	7	9	397_27	309_18	88_9	LOC_Os08g45220	1
PH-902	-	Chr01	34254423-34254674	64	-74	325	3_3	1_0	3	3	76_9	63_9	13_0	LOC_Os08g45220	1
PH-25	phasi_NO56	Chr01	34256928-34257179	2254	-95	346	6_5	3_3	8	7	1002_93	402_37	600_56	NA	0
PH-26	-	Chr01	34278634-34278885	21455	-53	304	4_1	1_0	1	5	231_3	212_3	19_0	LOC_Os01g59300	5
PH-27	phasi_NO58	Chr01	34285506-34285757	6621	-113	364	3_3	No	3	2	108_8	108_8	0_0	NA	0
PH-28	-	Chr01	34289649-34289900	3892	-53	304	6_4	3_3	7	5	470_50	433_38	37_12	Rep_LOC_Os11g14160	1
PH-29	-	Chr01	34291116-34291367	1216	-95	346	6_4	No	4	6	182_27	182_27	0_0	NA	0
PH-369	-	Chr02	109-360	..	ND	251	20_20	11_11	31	1	1488_1778	534_643	954_1135	Rep_ORSiTRTM00000003,RF00177	31
PH-370	-	Chr02	1486835-1487086	1486475	-53	304	8_4	No	4	5	225_38	225_38	0_0	NA	0
PH-371	-	Chr02	1487616-1487867	530	-32	283	5_4	No	4	3	151_20	151_20	0_0	NA	0
PH-372	-	Chr02	1494817-1495068	6950	-74	325	5_2	8_8	10	5	610_140	500_45	110_95	osa-MIR818	8
PH-373	-	Chr02	1512313-1512564	17245	ND	251	9_6	2_2	8	6	432_112	405_93	27_19	Rep_LOC_Os12g17240	1
PH-374	-	Chr02	1518227-1518478	5663	-292	543	4_4	1_1	5	3	635_67	242_27	393_40	NA	0
PH-375	phasi_NO69	Chr02	1520285-1520536	1807	-284	535	3_3	6_2	5	7	1120_130	769_91	351_39	LOC_Os02g03650	9
PH-376	phasi_NO70	Chr02	1522811-1523062	2275	-74	325	5_3	No	3	4	1700_136	1700_136	0_0	LOC_Os02g03650,LOC_Os02g036	5
PH-377	-	Chr02	1527441-1527692	4379	-118	369	7_4	1_1	5	7	1323_235	1200_217	123_18	NA	0
PH-378	-	Chr02	1528471-1528722	779	-11	262	9_6	No	6	7	187_32	187_32	0_0	NA	0
PH-379	phasi_NO73	Chr02	1530474-1530725	1752	-74	325	5_4	No	4	2	93_23	93_23	0_0	NA	0
PH-380	phasi_NO74	Chr02	1531583-1531834	858	-158	409	8_7	1_1	8	5	424_76	373_67	51_9	LOC_Os02g03670.6	3
PH-422	-	Chr02	6168859-6169110	4637025	ND	251	3_3	1_1	4	0	27_26	19_17	8_9	Rep_LOC_Os09g04270,Rep_LOC_	4
PH-367	-	Chr02	10631283-10631534	4462173	ND	251	4_4	No	4	3	111_26	111_26	0_0	NA	0
PH-368	phasi_NO85	Chr02	10654616-10654867	23082	ND	251	2_2	1_0	2	3	611_42	593_42	18_0	NA	0
PH-381	-	Chr02	15795713-15795964	5140846	ND	251	5_3	No	3	4	99_9	99_9	0_0	NA	0
PH-382	-	Chr02	16566165-16566416	770201	-32	283	5_3	No	3	4	317_34	317_34	0_0	NA	0
PH-383	-	Chr02	16567042-16567293	626	-32	283	4_3	1_0	3	5	203_16	190_16	13_0	NA	0
PH-384	phasi_NO94	Chr02	16578384-16578635	11091	-53	304	6_5	1_0	5	5	453_25	229_25	224_0	NA	0
PH-385	-	Chr02	16595554-16595805	16919	-28	279	3_1	No	1	3	56_2	56_2	0_0	NA	0
PH-386	-	Chr02	16596678-16596929	873	-95	346	3_3	2_1	4	5	142_15	76_13	66_2	LOC_Os02g28030.1	3
PH-387	-	Chr02	20200669-20200920	3603740	-343	594	8_4	6_4	8	10	814_100	654_83	160_17	NA	0
PH-388	phasi_NO156	Chr02	20252721-20252972	51801	-53	304	8_7	3_3	10	9	1400_122	1249_111	151_11	NA	0
PH-389	-	Chr02	20253983-20254234	1011	-156	407	3_2	1_1	3	3	88_9	74_7	14_2	NA	0
PH-390	phasi_NO157	Chr02	20257132-20257383	2898	-53	304	4_4	1_0	4	4	191_30	179_30	12_0	NA	0
PH-391	phasi_NO158	Chr02	20259469-20259720	2086	-11	262	13_9	1_1	10	11	1500_168	1445_160	55_8	NA	0
PH-392	phasi_NO159	Chr02	20262133-20262384	2413	-95	346	6_4	1_1	5	6	942_58	376_22	566_36	NA	0
PH-393	-	Chr02	20266964-20267215	4580	-95	346	4_4	No	4	3	124_25	124_25	0_0	NA	0
PH-394	-	Chr02	20270910-20271161	3695	-94	345	3_3	2_1	4	4	643_56	119_13	524_43	NA	0
PH-395	-	Chr02	20281812-20282063	10651	-32	283	3_3	No	3	2	91_23	91_23	0_0	NA	0
PH-396	phasi_NO161	Chr02	20285047-20285298	2984	-61	312	5_3	No	3	4	283_36	283_36	0_0	NA	0
PH-397	-	Chr02	20310107-20310358	24809	-32	283	5_3	No	3	5	352_38	352_38	0_0	NA	0
PH-398	phasi_NO98	Chr02	20334183-20334434	23825	-53	304	4_3	2_2	5	6	375_26	168_13	207_13	NA	0
PH-399	phasi_NO103	Chr02	20351504-20351755	17070	-74	325	6_4	2_2	6	7	361_34	272_26	89_8	NA	0
PH-400	phasi_NO105	Chr02	20358293-20358544	6538	-74	325	3_2	1_1	3	4	334_29	238_15	96_14	NA	0
PH-401	phasi_NO107	Chr02	20363540-20363791	4996	-53	304	6_6	No	6	4	235_26	235_26	0_0	NA	0
PH-402	phasi_NO110	Chr02	20393863-20394114	30072	-178	429	4_0	1_1	1	4	54_2	45_0	9_2	NA	0
PH-403	phasi_NO111	Chr02	20396760-20397011	2646	-32	283	10_9	1_0	9	6	1436_292	1388_292	48_0	NA	0
PH-404	phasi_NO112	Chr02	20397962-20398213	951	-11	262	5_4	No	4	3	177_27	177_27	0_0	NA	0
PH-405	-	Chr02	20399546-20399797	1333	ND	251	3_0	No	0	3	123_0	123_0	0_0	NA	0
PH-406	phasi_NO113	Chr02	20404001-20404252	4204	-200	451	3_2	4_0	2	7	253_18	163_18	90_0	NA	0
PH-407	phasi_NO114	Chr02	20481103-20481354	76851	-74	325	7_4	1_0	4	8	383_27	371_27	12_0	NA	0
PH-408	phasi_NO119	Chr02	22829596-22829847	2348242	-158	409	4_4	2_2	6	5	586_115	554_107	32_8	NA	0
PH-409	phasi_NO121	Chr02	22834051-22834302	4204	-32	283	6_6	No	6	5	463_114	463_114	0_0	NA	0
PH-410	phasi_NO122	Chr02	22840171-22840422	5869	-221	472	12_8	2_1	9	9	480_92	456_88	24_4	osa-MIR5514	1
PH-411	-	Chr02	22845839-22846090	5417	-158	409	3_2	1_1	3	3	91_15	74_13	17_2	LOC_Os08g01950	1
PH-412	phasi_NO123	Chr02	22852127-22852378	6037	-74	325	5_4	1_0	4	5	177_11	157_11	20_0	NA	0
PH-413	-	Chr02	22854883-22855134	2505	-116	367	3_0	No	0	3	37_0	37_0	0_0	NA	0
PH-414	-	Chr02	22858372-22858623	3238	-116	367	3_3	1_1	4	3	247_42	234_40	13_2	NA	0
PH-415	phasi_NO127	Chr02	22860955-22861206	2332	-95	346	9_7	2_1	8	10	854_147	808_142	46_5	NA	0
PH-416	phasi_NO128	Chr02	22864239-22864490	3033	-53	304	7_7	2_0	7	7	312_51	264_51	48_0	NA	0
PH-417	-	Chr02	24140132-24140383	1275642	ND	251	3_3	No	3	3	146_17	146_17	0_0	LOC_Os02g39920.1	3
PH-418	-	Chr02	28085686-28085937	3945303	257	508	4_4	4_4	8	0	120_99	66_64	54_35	Rep_LOC_Os12g12080,Rep_LOC_	8
PH-419	-	Chr02	28344295-28344546	258358	ND	251	3_3	7_7	10	0	163_150	29_33	134_117	osa-MIR812,osa-MIR1862,RF0106	10
PH-420	-	Chr02	35372426-35372677	7027880	ND	251	4_4	8_8	12	1	248_195	49_49	199_146	Rep_ORSgTEMT00901543,Rep_LO	12
PH-421	-	Chr02	35935961-35936212	563284	ND	251	16_16	5_5	21	0	1353_1592	152_147	1201_1445	Rep_ORSiTRTM00000003,RF00177	21
PH-424	-	Chr03	11276236-11276487	..	ND	251	13_13	47_47	60	6	11411_8134	9050_5892	2361_2242	LOC_Os05g48870.7,LOC_Os04g42	3
PH-425	-	Chr03	13894440-13894691	2617953	ND	251	2_2	No	2	0	27_18	27_18	0_0	Rep_LOC_Os06g27720	2
PH-426	phasi_NO133,pha	Chr03	18586037-18586288	4691346	ND	251	8_7	1_1	8	5	2827_733	2776_731	51_2	LOC_Os03g32499	9
PH-427	phasi_NO136	Chr03	18586973-18587224	685	ND	251	3_3	No	3	3	997_182	997_182	0_0	LOC_Os03g32499	3
PH-428	-	Chr03	18588362-18588613	1138	ND	251	4_3	1_1	4	4	274_26	249_21	25_5	LOC_Os03g32499	5
PH-429	phasi_NO482	Chr03	18589000-18589251	387	ND	251	8_6	1_1	7	9	399_46	187_23	212_23	LOC_Os03g32499	9
PH-430	phasi_NO305	Chr03	18591469-18591720	2218	ND	251	6_5	1_1	6	5	1173_114	1051_108	122_6	LOC_Os03g32499	7
PH-431	-	Chr03	21475038-21475289	2883318	ND	251	2_2	3_3	5	0	79_71	43_40	36_31	Rep_LOC_Os12g12080,Rep_ORSg	5
PH-432	-	Chr03	23105525-23105776	1630236	ND	251	4_4	3_3	7	0	51_50	29_21	22_29	Rep_LOC_Os09g04270,Rep_LOC_	7
PH-433	-	Chr03	26774384-26774635	3668608	-74	325	3_3	No	3	2	72_16	72_16	0_0	NA	0
PH-434	phasi_NO164	Chr03	26838775-26839026	64140	-32	283	5_5	1_1	6	2	710_199	682_189	28_10	osa-MIR5486	1
PH-435	phasi_NO165	Chr03	26841605-26841856	2579	-95	346	5_5	1_1	6	4	770_76	749_74	21_2	NA	0
PH-436	phasi_NO167	Chr03	26855187-26855438	13331	-74	325	3_3	No	3	2	77_10	77_10	0_0	NA	0
PH-437	-	Chr03	26859707-26859958	4269	-158	409	4_4	3_3	7	3	185_38	119_28	66_10	LOC_Os03g47490,LOC_Os03g474	7
PH-438	-	Chr03	27556341-27556592	696383	-196	447	4_0	1_1	1	5	262_33	69_0	193_33	LOC_Os03g48400	5
PH-439	phasi_NO170	Chr03	27557972-27558223	1380	445	696	5_4	1_1	5	2	115_20	106_16	9_4	NA	0
PH-440	phasi_NO171	Chr03	27559969-27560220	1746	-32	283	4_3	2_1	4	3	118_59	68_46	50_13	NA	0
PH-441	-	Chr03	27561001-27561252	781	-173	424	6_4	3_2	6	8	218_26	165_18	53_8	NA	0

Name	Alt_name	Chr	Positions (sRNAworkbench)	Dist_w/previous (bp)	MEME motif position (bp)	Size_w/MEME (bp)	Phased clusters nb (Ob_Og)	Unphased_clusters nb (Ob_Og)	Common clusters nb	Diff exp cluster nb	Total_reads nb (Ob_Og)	Total_phased_reads (Ob_Og)	Total_unphased_reads (Ob_Og)	Annotation_Pipeline	Annotated cluster nb
PH-442	phasi_NO172	Chr03	27563978-27564229	2726	-171	422	4_4	No	4	2	141_27	141_27	0_0	NA	0
PH-443	-	Chr03	27567071-27567322	2842	-176	427	6_5	3_1	6	7	1715_319	1476_317	239_2	NA	0
PH-444	-	Chr03	27574502-27574753	7180	-174	425	4_2	2_2	4	5	306_24	94_6	212_18	NA	0
PH-445	-	Chr03	27575685-27575936	932	-219	470	5_5	3_2	7	5	570_140	494_122	76_18	NA	0
PH-446	-	Chr03	27624773-27625024	48837	-137	388	4_3	4_2	5	6	197_15	106_10	91_5	NA	0
PH-447	phasi_NO173	Chr03	27633954-27634205	8930	-11	262	5_5	4_2	7	6	271_55	174_48	97_7	NA	0
PH-448	phasi_NO174	Chr03	27639924-27640175	5719	-116	367	7_7	1_1	8	5	297_86	207_74	90_12	NA	0
PH-449	phasi_NO175	Chr03	27641030-27641281	855	-74	325	10_9	2_2	11	6	502_98	483_90	19_8	NA	0
PH-450	-	Chr03	27642003-27642254	722	-74	325	3_2	1_0	2	3	367_55	331_55	36_0	NA	0
PH-451	-	Chr03	32275047-32275298	4632793	102	353	6_6	No	6	6	399_100	399_100	0_0	NA	0
PH-452	phasi_NO177	Chr03	32276367-32276618	1069	ND	251	7_6	No	6	6	1197_160	1197_160	0_0	NA	0
PH-453	phasi_NO180	Chr03	32278158-32278409	1540	ND	251	3_2	No	2	3	172_32	172_32	0_0	NA	0
PH-454	phasi_NO181	Chr03	32278976-32279227	567	ND	251	5_5	No	5	4	858_107	858_107	0_0	NA	0
PH-455	phasi_NO184	Chr03	32296076-32296327	16849	ND	251	3_2	No	2	2	91_9	91_9	0_0	NA	0
PH-456	phasi_NO186	Chr03	32298335-32298586	2008	ND	251	7_6	No	6	5	262_28	262_28	0_0	NA	0
PH-457	-	Chr03	32302320-32302571	3734	ND	251	4_3	No	3	4	229_13	229_13	0_0	NA	0
PH-458	-	Chr03	32305233-32305484	2662	ND	251	4_2	No	2	3	84_4	84_4	0_0	NA	0
PH-459	phasi_NO188	Chr03	32305957-32306208	473	ND	251	3_3	1_1	4	3	1772_192	1738_188	34_4	NA	0
PH-460	phasi_NO189	Chr03	32459130-32459381	152922	ND	251	4_2	No	2	4	173_15	173_15	0_0	NA	0
PH-461	-	Chr03	32477716-32477967	18335	ND	251	2_2	No	2	1	380_15	380_15	0_0	NA	0
PH-462	phasi_NO190	Chr03	32487508-32487759	9541	ND	251	4_2	No	2	4	322_15	322_15	0_0	NA	0
PH-463	-	Chr03	32496066-32496317	8307	ND	251	4_3	No	3	2	108_15	108_15	0_0	NA	0
PH-464	-	Chr03	32497374-32497625	1057	ND	251	5_5	No	5	5	357_32	357_32	0_0	NA	0
PH-465	phasi_NO192	Chr03	32498828-32499079	1203	ND	251	5_4	2_2	6	5	215_29	115_14	100_15	NA	0
PH-466	-	Chr03	32500229-32500480	1150	ND	251	5_3	1_0	3	6	447_60	436_60	11_0	NA	0
PH-467	phasi_NO193	Chr03	32503694-32503945	3214	ND	251	3_2	No	2	2	205_19	205_19	0_0	NA	0
PH-468	phasi_NO197	Chr03	32512725-32512976	8780	ND	251	5_2	No	2	5	250_10	250_10	0_0	NA	0
PH-469	-	Chr04	1004-1255	..	ND	251	20_20	11_11	31	1	1488_1778	1048_1219	440_559	Rep_ORSiTRTM00000003,RF00177	31
PH-590	-	Chr04	9094594-9094845	9093339	ND	251	6_6	13_13	19	0	199_272	46_64	153_208	RF00177;SSU_rRNA_bacteria;AC1	19
PH-470	-	Chr04	10644528-10644779	1549683	ND	251	3_3	No	3	0	20_16	20_16	0_0	Rep_LOC_Os09g04270,Rep_LOC_(	3
PH-472	phasi_NO201	Chr04	12693477-12693728	2048698	ND	251	6_4	No	4	6	143_12	143_12	0_0	NA	0
PH-474	-	Chr04	12975020-12975271	281292	ND	251	4_4	1_0	4	3	395_37	375_37	20_0	NA	0
PH-476	-	Chr04	13123948-13124199	148677	ND	251	3_0	No	0	3	53_0	53_0	0_0	NA	0
PH-478	phasi_NO205	Chr04	13164302-13164553	40103	ND	251	4_4	2_0	4	5	213_16	181_16	32_0	NA	0
PH-480	phasi_NO208	Chr04	13253758-13254009	89205	ND	251	3_2	No	2	3	127_12	127_12	0_0	Rep_LOC_Os07g41530	1
PH-481	-	Chr04	13281747-13281998	27738	ND	251	4_4	1_0	4	3	915_85	900_85	15_0	NA	0
PH-483	-	Chr04	13308746-13308997	26748	ND	251	5_3	No	3	4	95_15	95_15	0_0	NA	0
PH-485	phasi_NO213	Chr04	13341343-13341594	32346	ND	251	2_1	No	1	1	26_2	26_2	0_0	NA	0
PH-487	phasi_NO223	Chr04	14287138-14287389	945544	ND	251	5_1	No	1	5	225_8	225_8	0_0	NA	0
PH-488	phasi_NO222	Chr04	14289556-14289807	2167	ND	251	3_2	No	2	2	87_6	87_6	0_0	NA	0
PH-489	-	Chr04	14295665-14295916	5858	ND	251	2_1	No	1	2	153_4	0_0	153_4	NA	0
PH-491	-	Chr04	14565527-14565778	269611	ND	251	6_2	No	2	6	169_5	15_0	154_5	NA	0
PH-492	phasi_NO47	Chr04	14601266-14601517	35488	ND	251	5_3	No	3	5	200_17	200_17	0_0	NA	0
PH-493	phasi_NO48	Chr04	14602066-14602317	549	ND	251	4_3	No	3	4	502_73	456_69	46_4	NA	0
PH-494	-	Chr04	14621098-14621349	18781	-39	290	6_4	No	4	6	293_16	293_16	0_0	NA	0
PH-496	phasi_NO27	Chr04	14638124-14638375	16775	ND	251	3_1	No	1	2	51_7	51_7	0_0	NA	0
PH-497	-	Chr04	14639114-14639365	739	ND	251	6_2	1_0	2	7	146_9	13_0	133_9	LOC_Os04g26160	2
PH-498	phasi_NO26	Chr04	14642633-14642884	3268	ND	251	6_1	No	1	6	386_10	29_0	357_10	NA	0
PH-499	phasi_NO25	Chr04	14669054-14669305	26170	ND	251	8_4	No	4	7	554_18	554_18	0_0	NA	0
PH-500	-	Chr04	14685225-14685476	15920	-32	283	3_2	No	2	3	75_9	75_9	0_0	NA	0
PH-502	-	Chr04	14881392-14881643	195916	ND	251	3_1	No	1	3	270_3	270_3	0_0	NA	0
PH-503	-	Chr04	14916634-14916885	34991	-32	283	4_1	No	1	4	368_8	368_8	0_0	NA	0
PH-504	-	Chr04	14935593-14935844	18708	-32	283	5_4	1_0	4	4	333_43	313_43	20_0	NA	0
PH-505	-	Chr04	14995852-14996103	60008	-32	283	5_2	2_0	2	7	2718_148	2693_148	25_0	LOC_Os04g25740.1	1
PH-506	phasi_NO223	Chr04	15004358-15004609	8255	-200	451	6_2	No	2	6	248_10	23_2	225_8	NA	0
PH-508	-	Chr04	15116571-15116822	111962	ND	251	3_2	No	2	3	75_9	0_0	75_9	NA	0
PH-509	-	Chr04	15130243-15130494	13421	-53	304	7_6	2_2	8	4	206_49	116_22	90_27	Rep_LOC_Os12g18010,Rep_LOC_(	2
PH-510	phasi_NO89	Chr04	15172781-15173032	42287	-53	304	4_2	No	2	4	159_7	159_7	0_0	NA	0
PH-511	phasi_NO26	Chr04	15180657-15180908	7625	-32	283	7_2	No	2	7	435_12	420_12	15_0	NA	0
PH-512	-	Chr04	15184241-15184492	3333	-32	283	7_4	No	4	6	145_13	145_13	0_0	LOC_Os04g26160	2
PH-513	-	Chr04	15205729-15205980	21237	ND	251	4_2	No	2	4	203_10	203_10	0_0	NA	0
PH-514	phasi_NO47	Chr04	15222712-15222963	16732	ND	251	5_3	No	3	5	200_17	154_17	46_0	NA	0
PH-515	-	Chr04	15223514-15223765	551	ND	251	5_4	No	4	5	523_76	523_76	0_0	NA	0
PH-517	-	Chr04	15265397-15265648	41632	-32	283	5_2	No	2	5	154_5	154_5	0_0	NA	0
PH-518	-	Chr04	15272342-15272593	6694	-11	262	3_3	No	3	1	38_6	38_6	0_0	LOC_Os04g26200,LOC_Os04g262(	3
PH-519	-	Chr04	15274322-15274573	1729	-32	283	4_3	No	3	2	137_10	137_10	0_0	NA	0
PH-521	-	Chr04	17115995-17116246	1841422	-32	283	5_4	No	4	3	435_112	435_112	0_0	NA	0
PH-522	phasi_NO240	Chr04	17200102-17200353	83856	-179	430	6_6	7_7	13	8	1575_251	1170_188	405_63	NA	0
PH-523	phasi_NO241	Chr04	17211236-17211487	10883	-116	367	8_8	3_3	11	8	2865_604	1992_412	873_192	NA	0
PH-524	phasi_NO242	Chr04	17216646-17216897	5159	-53	304	5_5	No	5	4	615_79	615_79	0_0	NA	0
PH-525	phasi_NO243	Chr04	17226492-17226743	9595	-32	283	7_6	2_2	8	8	1552_199	1499_189	53_10	NA	0
PH-526	-	Chr04	17230069-17230320	3326	-116	367	4_4	1_1	5	3	203_42	135_25	68_17	NA	0
PH-527	phasi_NO244	Chr04	17241895-17242146	11575	-200	451	3_3	No	3	1	56_14	56_14	0_0	NA	0
PH-528	-	Chr04	17245566-17245817	3420	-32	283	4_3	1_0	3	4	186_39	173_39	13_0	NA	0
PH-529	phasi_NO246	Chr04	17250458-17250709	4641	-200	451	4_4	2_1	5	4	149_40	87_23	62_17	NA	0
PH-530	-	Chr04	17385333-17385584	134624	-95	346	3_3	1_1	4	2	236_40	225_36	11_4	NA	0
PH-531	phasi_NO251	Chr04	17391441-17391692	5857	-53	304	6_5	No	5	6	341_34	341_34	0_0	NA	0
PH-532	-	Chr04	17417138-17417389	25446	-72	323	3_2	2_2	4	3	493_52	380_42	113_10	NA	0
PH-533	phasi_NO253	Chr04	17419074-17419325	1685	-32	283	4_4	No	4	3	156_48	156_48	0_0	NA	0
PH-534	phasi_NO254	Chr04	17422424-17422675	3099	-74	325	5_4	No	4	4	129_15	129_15	0_0	NA	0
PH-535	phasi_NO255	Chr04	17423400-17423651	725	-32	283	5_4	No	4	4	333_49	333_49	0_0	NA	0
PH-536	phasi_NO259	Chr04	17464876-17465127	41225	-116	367	6_4	2_1	5	6	169_19	144_17	25_2	NA	0
PH-537	-	Chr04	17470966-17471217	5839	-137	388	3_3	1_1	4	2	151_38	137_34	14_4	NA	0
PH-538	phasi_NO261	Chr04	17471614-17471865	397	ND	251	5_5	No	5	3	116_18	116_18	0_0	NA	0
PH-539	-	Chr04	17473593-17473844	1728	-32	283	3_3	1_1	4	3	180_40	151_32	29_8	NA	0
PH-540	phasi_NO263	Chr04	17474246-17474497	402	-74	325	3_3	No	3	2	57_12	57_12	0_0	NA	0
PH-541	phasi_NO264	Chr04	17478236-17478487	3739	-32	283	5_3	No	3	5	574_81	574_81	0_0	NA	0
PH-542	-	Chr04	17578128-17578379	99641	-32	283	3_2	1_0	2	4	309_7	297_7	12_0	NA	0
PH-543	phasi_NO270	Chr04	17638061-17638312	59682	-53	304	3_0	No	0	3	159_0	159_0	0_0	Rep_LOC_Os04g29630	3
PH-544	phasi_NO272	Chr04	17656542-17656793	18230	-32	283	4_3	No	3	4	209_22	209_22	0_0	NA	0
PH-545	-	Chr04	17658633-17658884	1840	-32	283	6_4	1_0	4	6	1335_227	1321_227	14_0	NA	0
PH-546	-	Chr04	17661264-17661515	2380	-95	346	5_3	1_1	4	6	258_27	195_21	63_6	NA	0
PH-547	phasi_NO274	Chr04	17663211-17663462	1696	-116	367	3_3	No	3	2	100_13	100_13	0_0	NA	0
PH-548	-	Chr04	18158846-18159097	495384	-238	489	3_2	3_2	4	4	158_20	41_6	117_14	NA	0
PH-549	phasi_NO282	Chr04	18609710-18609961	450613	-95	346	3_3	1_1	4	1	61_11	48_9	13_2	LOC_Os04g31120.4,LOC_Os04g31	4
PH-550	phasi_NO305	Chr04	20982874-20983125	2372913	-32	283	7_6	1_1	7	6	1260_120	1138_114	122_6	LOC_Os03g32499	7
PH-551	-	Chr04	20985421-20985672	2296	-53	304	9_7	2_2	9	10	429_52	343_44	86_8	LOC_Os03g32499	11
PH-552	phasi_NO136	Chr04	20987767-20988018	2095	-32	283	4_3	No	3	4	1012_182	1012_182	0_0	LOC_Os03g32499	4
PH-553	phasi_NO483	Chr04	20988640-20988891	622	-74	325	8_7	1_1	8	5	1450_430	1399_428	51_2	LOC_Os03g32499	8
PH-554	-	Chr04	20990336-20990587	1445	-32	283	6_5	1_0	5	6	2222_219	2190_219	32_0	NA	0
PH-555	phasi_NO479	Chr04	20993484-20993735	2897	-53	304	7_4	No	4	7	244_15	116_12	128_3	LOC_Os06g20480.1,LOC_Os06g20	7
PH-556	-	Chr04	21017631-21017882	23896	-32	283	7_5	4_4	9	10	1829_209	1722_191	107_18	NA	0
PH-557	-	Chr04	21018778-21019029	896	257	508	7_7	6_3	10	11	2482_283	2172_272	310_11	NA	0
PH-558	phasi_NO339	Chr04	21024305-21024556	5276	-53	304	5_4	2_2	6	6	428_33	373_29	55_4	NA	0
PH-559	-	Chr04	21027524-21027775	2968	-32	283	4_4	No	4	4	124_22	124_22	0_0	NA	0
PH-560	-	Chr04	21032411-21032662	4636	-32	283	10_9	5_0	9	14	1149_229	1022_229	127_0	NA	0
PH-561	-	Chr04	21047701-21047952	15039	-32	283	9_7	No	7	8	2036_199	2036_199	0_0	NA	0
PH-562	-	Chr04	21048604-21048855	652	-116	367	5_2	3_1	3	8	296_13	211_11	85_2	NA	0
PH-563	-	Chr04	21051273-21051524	2418	-32	283	8_4	1_0	4	9	276_19	261_19	15_0	NA	0
PH-564	-	Chr04	21056277-21056528	4753	-32	283	4_3	No	3	4	388_49	388_49	0_0	NA	0
PH-565	phasi_NO308	Chr04	21063551-21063802	7023	367	618	3_3	1_1	4	3	147_19	137_17	10_2	NA	0
PH-566	-	Chr04	21067168-21067419	3366	-158	409	4_2	1_1	3	5	177_21	139_10	38_11	NA	0
PH-567	-	Chr04	21075332-21075583	7913	-32	283	3_2	No	2	3	341_29	341_29	0_0	NA	0
PH-568	-	Chr04	21077168-21077419	1585	-158	409	3_2	5_2	4	6	244_25	113_15	131_10	LOC_Os04g34780,LOC_Os04g347(	6

Name	Alt_name	Chr	Positions (sRNAworkbench)	Dist_w/previous (bp)	MEME motif position (bp)	Size_w/MEME (bp)	Phased clusters nb (Ob_Og)	Unphased_clusters nb (Ob_Og)	Common clusters nb	Diff exp cluster nb	Total_reads nb (Ob_Og)	Total_phased_reads (Ob_Og)	Total_unphased_reads (Ob_Og)	Annotation_Pipeline	Annotated cluster nb
PH-569	phasi_NO314	Chr04	21087516-21087767	10097	-32	283	7_5	No	5	4	200_25	200_25	0_0	NA	0
PH-570	phasi_NO315	Chr04	21090361-21090612	2594	-116	367	9_8	2_2	10	10	759_99	250_35	509_64	NA	0
PH-571	-	Chr04	21092610-21092861	1998	-74	325	3_3	1_1	4	4	428_48	390_37	38_11	NA	0
PH-572	-	Chr04	21095370-21095621	2509	-32	283	6_2	1_1	3	7	167_13	130_9	37_4	NA	0
PH-573	-	Chr04	21099824-21100075	4203	-200	451	8_4	5_4	8	10	1448_116	1176_97	272_19	NA	0
PH-574	-	Chr04	21105467-21105718	5392	-95	346	8_6	1_1	7	8	370_38	111_17	259_21	NA	0
PH-966	-	Chr04	21105509-21105760	209	-137	388	4_3	1_1	4	5	264_21	200_16	64_5	NA	0
PH-575	-	Chr04	21121522-21121773	15762	-74	325	6_5	2_2	7	6	679_101	560_87	119_14	NA	0
PH-576	-	Chr04	21137865-21138116	16092	-74	325	6_6	1_0	6	5	463_56	444_56	19_0	NA	0
PH-577	phasi_NO318	Chr04	21141581-21141832	3465	-53	304	6_0	2_0	0	8	386_0	158_0	228_0	NA	0
PH-578	-	Chr04	21143188-21143439	1356	-32	283	5_4	2_2	6	7	351_37	252_31	99_6	NA	0
PH-579	-	Chr04	21144830-21145081	1391	-32	283	5_3	1_1	4	5	242_23	233_21	9_2	NA	0
PH-580	-	Chr04	21149610-21149861	4529	-11	262	6_4	1_1	5	7	311_32	285_28	26_4	NA	0
PH-581	-	Chr04	21161408-21161659	11547	-53	304	5_4	1_0	4	5	436_35	418_35	18_0	NA	0
PH-582	phasi_NO319	Chr04	21165734-21165985	4075	-158	409	6_6	1_1	7	5	1428_189	1299_173	129_16	NA	0
PH-583	phasi_NO320	Chr04	21167759-21168010	1774	-116	367	8_7	3_2	9	10	6707_686	350_49	6357_637	NA	0
PH-584	-	Chr04	21181136-21181387	13126	-32	283	4_3	No	3	3	106_17	106_17	0_0	NA	0
PH-585	phasi_NO323	Chr04	21191161-21191412	9774	-53	304	4_1	1_1	2	5	101_10	63_5	38_5	NA	0
PH-586	phasi_NO326	Chr04	21200702-21200953	9290	-74	325	5_2	1_0	2	5	103_6	89_6	14_0	NA	0
PH-587	-	Chr04	21223558-21223809	22605	-158	409	3_1	1_0	1	4	117_3	96_3	21_0	NA	0
PH-588	phasi_NO329	Chr04	21225840-21226091	2031	-254	505	5_4	2_2	6	7	515_59	441_51	74_8	NA	0
PH-589	-	Chr04	24905399-24905650	3679308	ND	251	15_15	29_28	43	12	4422_2837	3532_2267	890_570	LOC_Os04g42050	44
PH-601	-	Chr05	16-267	..	ND	251	20_20	10_10	30	1	1465_1768	572_710	893_1058	Rep_ORSiTRTM00000003,RF00177	30
PH-603	-	Chr05	2077235-2077486	2076968	ND	251	2_2	1_1	3	0	27_33	19_27	8_6	Rep_ORSgTEMT00500708,Rep_LO	3
PH-641	-	Chr05	3086673-3086924	1009187	-190	441	8_7	3_3	10	6	373_87	289_75	84_12	NA	0
PH-642	-	Chr05	3092726-3092977	5802	-53	304	3_3	2_2	5	4	125_21	92_13	33_8	LOC_Os05g06160	5
PH-643	phasi_NO349	Chr05	3101560-3101811	8583	-95	346	6_5	9_2	7	13	909_92	614_77	295_15	NA	0
PH-644	phasi_NO350	Chr05	3102631-3102882	820	-95	346	3_2	No	2	3	341_52	341_52	0_0	NA	0
PH-645	phasi_NO351	Chr05	3104481-3104732	1599	-263	514	3_3	1_0	3	2	89_137	57_137	32_0	NA	0
PH-646	-	Chr05	3113786-3114037	9054	-32	283	5_5	No	5	3	179_25	179_25	0_0	NA	0
PH-647	-	Chr05	3117499-3117750	3462	-32	283	3_2	No	2	3	77_5	77_5	0_0	NA	0
PH-648	phasi_NO352	Chr05	3123438-3123689	5688	-74	325	6_5	3_3	8	5	262_61	226_44	36_17	NA	0
PH-649	phasi_NO353	Chr05	3124528-3124779	839	-116	367	5_4	No	4	3	539_97	539_97	0_0	NA	0
PH-650	phasi_NO355	Chr05	3131693-3131944	6914	-242	493	3_2	3_2	4	6	477_73	97_22	380_51	NA	0
PH-651	-	Chr05	3133811-3134062	1867	-116	367	5_4	2_2	6	5	329_69	152_23	177_46	NA	0
PH-652	-	Chr05	3136524-3136775	2462	-53	304	4_2	No	2	2	52_6	52_6	0_0	NA	0
PH-653	phasi_NO356	Chr05	3140558-3140809	3783	-95	346	12_10	No	10	12	803_126	803_126	0_0	NA	0
PH-654	-	Chr05	3146517-3146768	5708	-32	283	6_6	No	6	2	120_43	120_43	0_0	NA	0
PH-655	-	Chr05	3149519-3149770	2751	-32	283	4_3	No	3	2	86_14	86_14	0_0	NA	0
PH-656	phasi_NO360	Chr05	3159932-3160183	10162	-137	388	5_5	2_1	6	5	891_129	859_124	32_5	NA	0
PH-657	-	Chr05	3169026-3169277	8843	-305	556	5_5	4_3	8	6	351_86	272_72	79_14	NA	0
PH-659	phasi_NO362	Chr05	6099519-6099770	2930242	-32	283	7_4	No	4	6	717_106	717_106	0_0	NA	0
PH-660	-	Chr05	6104771-6105022	5001	-32	283	4_3	No	3	4	325_68	325_68	0_0	NA	0
PH-661	-	Chr05	6131590-6131841	26568	-32	283	5_5	No	5	4	173_22	173_22	0_0	NA	0
PH-662	phasi_NO364	Chr05	6133332-6133583	1491	-200	451	3_1	2_0	1	4	2912_2	48_2	2864_0	NA	0
PH-985	phasi_NO365	Chr05	6140612-6140863	7029	-157	408	1_4	5_3	3	4	169_118	3_84	166_34	NA	0
PH-663	phasi_NO366	Chr05	6143841-6144092	2978	-32	283	6_4	1_1	5	5	219_41	152_24	67_17	NA	0
PH-665	-	Chr05	8139379-8139630	1995287	ND	251	4_2	No	2	4	291_13	291_13	0_0	NA	0
PH-666	phasi_NO371	Chr05	8177448-8177699	37818	ND	251	3_2	No	2	3	67_6	67_6	0_0	LOC_Os05g14480	3
PH-667	phasi_NO374	Chr05	8246992-8247243	69293	-164	415	3_3	3_3	6	4	296_55	254_48	42_7	LOC_Os05g14540	6
PH-591	phasi_NO378	Chr05	13959829-13960080	5712586	ND	251	5_5	1_1	6	6	660_83	619_81	41_2	NA	0
PH-592	phasi_NO380	Chr05	13962524-13962775	2444	ND	251	6_4	No	4	6	975_20	975_20	0_0	osa-MIR5516	1
PH-593	phasi_NO379	Chr05	13976622-13976873	13847	ND	251	8_7	No	7	6	1486_300	1486_300	0_0	NA	0
PH-594	phasi_NO380	Chr05	13979283-13979534	2410	ND	251	4_2	No	2	4	400_18	400_18	0_0	osa-MIR5516	1
PH-595	phasi_NO381	Chr05	13984468-13984719	4934	ND	251	9_7	No	7	9	2080_279	2080_279	0_0	NA	0
PH-596	phasi_NO382	Chr05	13986772-13987023	2053	ND	251	6_5	No	5	5	522_76	522_76	0_0	NA	0
PH-597	-	Chr05	14006551-14006802	19528	ND	251	3_1	1_1	2	4	269_13	70_3	199_10	NA	0
PH-598	-	Chr05	14006866-14007117	64	ND	251	4_3	1_0	3	5	889_38	878_38	11_0	NA	0
PH-599	-	Chr05	14017728-14017979	10611	ND	251	4_1	No	1	4	153_11	153_11	0_0	NA	0
PH-600	-	Chr05	14746920-14747171	728941	ND	251	5_4	2_2	6	5	842_50	804_43	38_7	Rep_RN_42-23	7
PH-602	phasi_NO385	Chr05	19141869-19142120	4394698	-74	325	7_6	No	6	6	776_84	776_84	0_0	NA	0
PH-604	-	Chr05	24789997-24790248	5647877	-179	430	4_4	1_1	5	4	307_32	278_27	29_5	NA	0
PH-605	-	Chr05	24797748-24797999	7500	-93	344	4_2	2_0	2	5	139_5	104_5	35_0	NA	0
PH-606	-	Chr05	24802174-24802425	4175	-53	304	5_1	3_3	4	4	109_20	85_4	24_16	Rep_LOC_Os12g10770	3
PH-607	phasi_NO389	Chr05	24806141-24806392	3716	-53	304	9_8	No	8	8	1041_156	1041_156	0_0	NA	0
PH-608	phasi_NO391	Chr05	24825085-24825336	18693	-95	346	4_2	3_1	3	7	229_10	119_5	110_5	NA	0
PH-609	-	Chr05	24833655-24833906	8319	-32	283	5_2	1_1	3	6	601_22	503_18	98_4	LOC_Os05g42390.1	2
PH-610	phasi_NO398	Chr05	24867350-24867601	33444	-32	283	5_3	No	3	2	72_11	72_11	0_0	NA	0
PH-611	phasi_NO399	Chr05	24870411-24870662	2810	-53	304	5_3	2_0	3	7	554_26	528_26	26_0	NA	0
PH-612	-	Chr05	24872805-24873056	2143	-32	283	10_8	1_1	9	11	3007_232	2987_229	20_3	NA	0
PH-613	phasi_NO400	Chr05	24877466-24877717	4410	-116	367	10_8	2_0	8	12	938_63	893_63	45_0	LOC_Os05g42428.1,LOC_Os05g42	10
PH-614	phasi_NO402	Chr05	24893510-24893761	15793	-74	325	12_10	2_2	12	9	1454_68	530_63	924_5	NA	0
PH-615	phasi_NO403	Chr05	24896162-24896413	2401	-74	325	7_1	1_0	1	8	882_2	865_2	17_0	NA	0
PH-616	-	Chr05	24906104-24906355	9691	-53	304	5_2	1_1	3	6	297_22	246_20	51_2	NA	0
PH-617	phasi_NO404	Chr05	24907229-24907480	874	-11	262	4_2	No	2	3	66_7	66_7	0_0	NA	0
PH-618	phasi_NO404	Chr05	24907596-24907847	116	-378	629	8_6	No	6	6	452_64	386_57	66_7	NA	0
PH-619	phasi_NO405	Chr05	24908243-24908494	396	-137	388	8_6	1_0	6	5	245_17	228_17	17_0	NA	0
PH-620	phasi_NO406	Chr05	24912484-24912735	3990	-116	367	4_3	5_1	4	9	4293_290	313_41	3980_249	NA	0
PH-621	phasi_NO408	Chr05	24953422-24953673	40687	-199	450	4_3	5_2	5	7	2288_88	2042_71	246_17	NA	0
PH-622	phasi_NO409	Chr05	24962418-24962669	8745	-53	304	12_9	1_0	9	13	4160_347	4145_347	15_0	NA	0
PH-623	phasi_NO410	Chr05	24965495-24965746	2826	-32	283	12_7	1_0	7	11	425_24	399_24	26_0	NA	0
PH-624	-	Chr05	24968712-24968963	2966	-137	388	3_1	1_0	1	4	1321_60	1300_60	21_0	NA	0
PH-625	-	Chr05	25062333-25062584	93370	-136	387	4_4	No	4	1	50_10	50_10	0_0	NA	0
PH-626	-	Chr05	25066722-25066973	4138	-32	283	5_4	No	4	4	755_71	755_71	0_0	NA	0
PH-627	-	Chr05	25068858-25069109	1885	-32	283	4_1	No	1	4	240_4	226_4	14_0	NA	0
PH-628	phasi_NO414	Chr05	25071557-25071808	2448	-137	388	3_1	No	1	3	119_7	119_7	0_0	NA	0
PH-629	-	Chr05	25077982-25078233	6174	-200	451	4_3	1_1	4	4	183_19	169_17	14_2	LOC_Os05g43190.1	4
PH-630	phasi_NO416	Chr05	25089698-25089949	11465	-32	283	13_8	1_1	9	14	4337_259	4301_257	36_2	NA	0
PH-631	phasi_NO417	Chr05	25092131-25092382	2182	-32	283	8_4	No	4	6	370_19	370_19	0_0	NA	0
PH-632	phasi_NO418	Chr05	25097083-25097334	4701	-32	283	3_2	No	2	3	171_8	171_8	0_0	NA	0
PH-633	phasi_NO420	Chr05	25102842-25103093	5508	-53	304	3_1	1_0	1	4	158_6	142_6	16_0	NA	0
PH-634	phasi_NO421	Chr05	25105697-25105948	2604	-53	304	10_6	No	6	9	297_27	297_27	0_0	NA	0
PH-635	phasi_NO423	Chr05	25129485-25129736	23537	-32	283	7_6	No	6	6	302_33	302_33	0_0	NA	0
PH-636	phasi_NO424	Chr05	25130875-25131126	1139	-137	388	5_4	2_1	5	6	277_26	227_20	50_6	NA	0
PH-637	phasi_NO426	Chr05	25140326-25140577	9200	-116	367	4_2	1_0	2	4	114_4	85_4	29_0	NA	0
PH-638	phasi_NO427	Chr05	25143605-25143856	3028	-11	262	9_7	No	7	7	423_31	423_31	0_0	NA	0
PH-639	phasi_NO428	Chr05	25145684-25145935	1828	-95	346	8_7	1_1	8	8	816_92	778_89	38_3	NA	0
PH-640	-	Chr05	25873154-25873405	727219	ND	251	2_2	1_1	3	0	27_33	19_27	8_6	Rep_ORSgTEMT00500708,Rep_LO	3
PH-755	-	Chr06	672634-672885	..	ND	251	8_8	No	8	8	1904_278	1904_278	0_0	NA	0
PH-756	-	Chr06	673573-673824	688	-36	287	6_4	1_1	5	6	257_34	232_28	25_6	osa-MIR2118	1
PH-685	-	Chr06	1352020-1352271	678196	ND	251	2_2	1_1	3	0	27_33	19_27	8_6	Rep_ORSgTEMT00500708,Rep_LO	3
PH-697	phasi_NO429	Chr06	1794755-1795006	442484	-74	325	3_3	No	3	0	65_30	65_30	0_0	NA	0
PH-698	phasi_NO430	Chr06	1795830-1796081	824	-74	325	3_3	No	3	0	48_24	48_24	0_0	NA	0
PH-699	-	Chr06	1797816-1798067	1735	297	548	5_5	21_21	25	4	818_665	237_60	581_605	LOC_Os12g07990,osa-MIR812,osa	24
PH-700	-	Chr06	1802025-1802276	3958	ND	251	5_4	No	4	2	74_19	74_19	0_0	NA	0
PH-740	-	Chr06	3712482-3712733	1910206	ND	251	3_3	No	3	0	23_29	23_29	0_0	Rep_LOC_Os12g43165,Rep_LOC_	3
PH-741	-	Chr06	4734626-4734877	1021893	ND	251	4_4	No	4	3	147_17	147_17	0_0	NA	0
PH-742	phasi_NO435	Chr06	4740417-4740668	5540	ND	251	7_5	No	5	6	477_38	477_38	0_0	NA	0
PH-743	phasi_NO436	Chr06	4741135-4741386	467	ND	251	4_3	No	3	1	52_13	39_13	13_0	NA	0
PH-744	-	Chr06	4753604-4753855	12218	-32	283	5_3	No	3	3	131_19	114_14	17_5	NA	0
PH-745	-	Chr06	4758778-4759029	4923	ND	251	8_5	No	5	4	406_67	406_67	0_0	NA	0
PH-746	-	Chr06	4764782-4765033	5753	ND	251	6_4	No	4	5	292_37	292_37	0_0	NA	0
PH-747	phasi_NO438	Chr06	4766510-4766761	1477	ND	251	7_5	3_2	7	8	617_100	519_88	98_12	LOC_Os03g57290.1	1
PH-748	-	Chr06	4769412-4769663	2651	ND	251	6_6	2_1	7	4	530_127	196_68	334_59	NA	0

Name	Alt_name	Chr	Positions (sRNAworkbench)	Dist_w/previous (bp)	MEME motif position (bp)	Size_w/MEME (bp)	Phased clusters nb (Ob_Og)	Unphased_clusters nb (Ob_Og)	Common clusters nb	Diff exp cluster nb	Total_reads nb (Ob_Og)	Total_phased_reads (Ob_Og)	Total_unphased_reads (Ob_Og)	Annotation_Pipeline	Annotated cluster nb
PH-749	phasi_NO439	Chr06	4771772-4772023	2109	ND	251	4_2	No	2	4	273_8	273_8	0_0	NA	0
PH-750	-	Chr06	4775355-4775606	3332	ND	251	6_5	No	5	4	457_54	457_54	0_0	NA	0
PH-751	phasi_NO440	Chr06	4777608-4777859	2002	ND	251	8_7	No	7	3	156_44	156_44	0_0	NA	0
PH-752	-	Chr06	4787002-4787253	9143	ND	251	3_2	No	2	2	192_25	192_25	0_0	NA	0
PH-753	phasi_NO441	Chr06	4789198-4789449	1945	ND	251	5_1	No	1	5	286_5	286_5	0_0	NA	0
PH-754	phasi_NO442	Chr06	4790714-4790965	1265	ND	251	2_0	No	0	2	27_0	27_0	0_0	NA	0
PH-757	-	Chr06	7791709-7791960	3000744	ND	251	3_2	2_2	4	1	50_41	31_13	19_28	RF01058;MIR806;AAAA02032891.	5
PH-758	phasi_NO446	Chr06	9453840-9454091	1661880	ND	251	3_0	No	0	3	99_0	99_0	0_0	NA	0
PH-759	phasi_NO461	Chr06	9577636-9577887	123545	ND	251	3_3	No	3	3	417_63	417_63	0_0	NA	0
PH-760	phasi_NO463	Chr06	9583382-9583633	5495	ND	251	9_8	No	8	8	419_78	419_78	0_0	NA	0
PH-761	phasi_NO467	Chr06	9660026-9660277	76393	ND	251	6_5	No	5	3	1643_196	1643_196	0_0	NA	0
PH-762	phasi_NO470	Chr06	9667706-9667957	7429	ND	251	3_3	No	3	2	157_9	157_9	0_0	NA	0
PH-763	phasi_NO471	Chr06	9669236-9669487	1279	ND	251	5_2	No	2	5	153_10	153_10	0_0	LOC_Os08g29510	1
PH-764	phasi_NO472	Chr06	9676418-9676669	6931	ND	251	3_3	No	3	2	127_16	127_16	0_0	Rep_LOC_Os02g33370	1
PH-765	phasi_NO473	Chr06	9677357-9677608	688	ND	251	4_0	No	0	4	84_0	84_0	0_0	NA	0
PH-766	phasi_NO477	Chr06	9679446-9679697	1838	ND	251	5_3	No	3	4	120_10	120_10	0_0	NA	0
PH-767	-	Chr06	9686478-9686729	6781	ND	251	6_6	No	6	5	205_39	205_39	0_0	NA	0
PH-768	-	Chr06	9690805-9691056	4076	ND	251	3_2	No	2	2	112_16	112_16	0_0	NA	0
PH-769	-	Chr06	9696515-9696766	5459	ND	251	3_3	1_1	4	3	301_50	288_45	13_5	NA	0
PH-668	phasi_NO138	Chr06	11761286-11761537	2064520	ND	251	5_3	No	3	5	196_13	196_13	0_0	LOC_Os06g20480.1,LOC_Os06g20	5
PH-669	-	Chr06	11763875-11764126	2338	ND	251	5_4	1_0	4	5	2198_215	2166_215	32_0	NA	0
PH-670	phasi_NO133, pha	Chr06	11765631-11765882	1505	ND	251	8_7	1_1	8	5	2827_733	2776_731	51_2	LOC_Os03g32499	9
PH-671	phasi_NO136	Chr06	11766567-11766818	685	ND	251	3_3	No	3	3	997_182	997_182	0_0	LOC_Os03g32499	3
PH-672	-	Chr06	11767958-11768209	1140	ND	251	3_2	1_1	3	3	258_24	0_0	258_24	LOC_Os03g32499	4
PH-673	-	Chr06	11768604-11768855	395	ND	251	8_6	1_1	7	9	399_46	272_30	127_16	LOC_Os03g32499	9
PH-674	phasi_NO305	Chr06	11771084-11771335	2229	ND	251	7_6	1_1	7	6	1260_120	1138_114	122_6	LOC_Os03g32499	7
PH-675	phasi_NO138	Chr06	11811127-11811378	39792	ND	251	6_4	No	4	6	230_15	144_15	86_0	LOC_Os06g20480.1,LOC_Os06g20	6
PH-676	-	Chr06	11813726-11813977	2348	ND	251	6_5	1_0	5	6	2222_219	2204_219	18_0	NA	0
PH-677	phasi_NO133	Chr06	11815482-11815733	1505	ND	251	8_7	1_1	8	5	2827_733	2786_731	41_2	LOC_Os03g32499	9
PH-678	-	Chr06	11816418-11816669	685	ND	251	3_3	No	3	3	997_182	997_182	0_0	LOC_Os03g32499	3
PH-679	-	Chr06	11817809-11818060	1140	ND	251	4_3	1_1	4	4	274_26	262_21	12_5	LOC_Os03g32499	5
PH-680	phasi_NO482	Chr06	11818475-11818726	415	ND	251	8_6	1_1	7	9	399_46	187_23	212_23	LOC_Os03g32499	9
PH-681	phasi_NO305	Chr06	11820955-11821206	2229	ND	251	7_6	1_1	7	6	1260_120	1162_114	98_6	LOC_Os03g32499	7
PH-682	phasi_NO489	Chr06	12043130-12043381	221924	ND	251	5_4	No	4	5	777_58	777_58	0_0	NA	0
PH-683	-	Chr06	12050090-12050341	6709	ND	251	6_5	No	5	5	1431_155	1431_155	0_0	NA	0
PH-684	phasi_NO496	Chr06	13096361-13096612	1046020	ND	251	3_3	No	3	2	49_12	49_12	0_0	NA	0
PH-686	phasi_NO491	Chr06	16916375-16916626	3819763	-32	283	5_2	No	2	5	128_6	128_6	0_0	LOC_Os06g29490.2	5
PH-687	phasi_NO494	Chr06	16973406-16973657	56780	-74	325	6_2	3_2	4	7	530_30	311_16	219_14	NA	0
PH-688	phasi_NO496	Chr06	17666326-17666577	692669	ND	251	4_2	1_1	3	4	116_11	105_8	11_3	NA	0
PH-689	-	Chr06	17672875-17673126	6298	-53	304	3_2	No	2	2	78_12	78_12	0_0	osa-MIR819	1
PH-690	phasi_NO497	Chr06	17694938-17695189	21812	-32	283	6_5	No	5	4	237_28	237_28	0_0	NA	0
PH-691	phasi_NO498	Chr06	17697557-17697808	2368	347	598	9_8	3_2	10	10	1317_174	1174_155	143_19	NA	0
PH-692	phasi_NO501	Chr06	17754638-17754889	56830	-53	304	3_2	No	2	3	71_9	71_9	0_0	LOC_Os01g34080	1
PH-693	phasi_NO502	Chr06	17769213-17769464	14324	-137	388	6_0	4_3	3	7	367_17	335_0	32_17	NA	0
PH-694	phasi_NO503	Chr06	17771673-17771924	2209	-32	283	6_5	1_1	6	6	1434_195	1424_152	10_43	LOC_Os06g30680,LOC_Os06g306	7
PH-695	phasi_NO504	Chr06	17784816-17785067	12892	-32	283	3_2	No	2	3	861_145	861_145	0_0	NA	0
PH-696	phasi_NO505	Chr06	17788501-17788752	3434	-74	325	6_0	3_1	1	9	339_22	190_0	149_22	NA	0
PH-701	-	Chr06	23171349-23171600	5382597	305	556	4_4	No	4	4	307_18	307_18	0_0	NA	0
PH-702	-	Chr06	23178446-23178697	6846	ND	251	3_2	No	2	3	203_23	203_23	0_0	NA	0
PH-703	phasi_NO518	Chr06	23179718-23179969	1021	ND	251	4_3	1_1	4	3	270_34	58_9	212_25	NA	0
PH-704	phasi_NO520	Chr06	24600681-24600932	1420712	-95	346	10_8	No	8	9	1540_171	1540_171	0_0	NA	0
PH-705	phasi_NO521	Chr06	24602308-24602559	1376	-137	388	5_3	2_1	4	7	211_39	177_37	34_2	NA	0
PH-706	phasi_NO523	Chr06	24608875-24609126	6316	-200	451	3_3	5_4	7	7	1368_212	1158_191	210_21	NA	0
PH-707	phasi_NO524	Chr06	24610793-24611044	1667	ND	251	4_3	2_1	4	5	108_23	89_20	19_3	NA	0
PH-708	phasi_NO526	Chr06	24617212-24617463	6168	-116	367	6_3	1_1	4	5	131_13	125_8	6_5	NA	0
PH-709	-	Chr06	24618443-24618694	980	-179	430	3_1	No	1	2	62_4	62_4	0_0	NA	0
PH-710	-	Chr06	24630211-24630462	11517	-53	304	7_5	2_1	6	8	278_30	167_18	111_12	NA	0
PH-711	-	Chr06	24639274-24639525	8812	-116	367	3_2	2_2	4	3	172_53	58_10	114_43	NA	0
PH-712	phasi_NO532	Chr06	24650491-24650742	10966	-53	304	10_10	2_1	11	11	1181_241	789_189	392_52	NA	0
PH-713	phasi_NO533	Chr06	24657180-24657431	6438	-53	304	8_7	No	7	5	547_60	547_60	0_0	NA	0
PH-714	phasi_NO535	Chr06	24661231-24661482	3800	-95	346	6_3	1_1	4	6	363_38	302_27	61_11	NA	0
PH-990	phasi_NO536	Chr06	24663303-24663554	1821	-115	366	3_3	6_3	6	5	251_48	36_16	215_32	NA	0
PH-715	phasi_NO537	Chr06	24674952-24675203	11398	-11	262	5_5	1_1	6	3	470_72	259_41	211_31	NA	0
PH-716	phasi_NO538	Chr06	24676851-24677102	1648	-115	366	4_0	1_0	0	5	188_0	167_0	21_0	NA	0
PH-717	phasi_NO540	Chr06	24682211-24682462	5109	-32	283	4_4	1_1	5	3	129_16	65_14	64_2	NA	0
PH-718	-	Chr06	24762719-24762970	80257	-32	283	3_3	No	3	3	134_12	134_12	0_0	NA	0
PH-719	phasi_NO551	Chr06	24772065-24772316	9095	-53	304	8_6	5_2	8	11	804_58	431_42	373_16	NA	0
PH-720	phasi_NO550	Chr06	24774379-24774630	2063	-53	304	7_7	2_1	8	6	683_87	643_85	40_2	NA	0
PH-721	-	Chr06	24784675-24784926	10045	-74	325	7_6	1_1	7	7	945_142	910_138	35_4	NA	0
PH-722	-	Chr06	27833738-27833989	3048812	ND	251	4_4	6_6	10	0	148_127	67_48	81_79	osa-MIR806,RF01058;MIR806;AAA	10
PH-723	phasi_NO557	Chr06	28293560-28293811	459571	ND	251	6_5	1_0	5	6	722_97	707_97	15_0	NA	0
PH-724	phasi_NO558	Chr06	28295251-28295502	1440	ND	251	6_5	2_2	7	5	471_64	437_59	34_5	NA	0
PH-725	phasi_NO562	Chr06	28299407-28299658	3905	ND	251	3_2	1_1	3	3	182_25	171_22	11_3	NA	0
PH-726	-	Chr06	28301409-28301660	1751	ND	251	4_4	No	4	2	1244_135	1244_135	0_0	NA	0
PH-727	phasi_NO563	Chr06	28302316-28302567	656	ND	251	3_3	No	3	2	630_66	630_66	0_0	NA	0
PH-728	phasi_NO565	Chr06	28307755-28308006	5188	ND	251	3_3	No	3	3	702_78	702_78	0_0	NA	0
PH-729	-	Chr06	28309062-28309313	1056	ND	251	10_8	No	8	8	741_153	741_153	0_0	NA	0
PH-730	phasi_NO566	Chr06	28313250-28313501	3937	64	315	5_5	3_3	8	6	935_117	348_57	587_60	NA	0
PH-731	-	Chr06	28315197-28315448	1696	ND	251	2_1	No	1	1	28_2	28_2	0_0	NA	0
PH-732	phasi_NO568	Chr06	28316261-28316512	813	ND	251	2_1	No	1	1	26_2	26_2	0_0	NA	0
PH-733	phasi_NO570	Chr06	28324263-28324514	7751	ND	251	4_1	No	1	4	171_27	171_27	0_0	NA	0
PH-734	phasi_NO570	Chr06	28324515-28324766	1	ND	251	4_2	No	2	4	949_106	949_106	0_0	NA	0
PH-735	-	Chr06	28325906-28326157	1140	ND	251	3_1	No	1	2	44_2	44_2	0_0	NA	0
PH-736	phasi_NO572	Chr06	28329626-28329877	3469	ND	251	4_2	No	2	3	109_12	109_12	0_0	NA	0
PH-737	phasi_NO575	Chr06	28334546-28334797	4669	ND	251	4_1	1_0	1	4	103_3	87_3	16_0	LOC_Os06g46650.1	3
PH-738	-	Chr06	28338158-28338409	3361	ND	251	6_5	2_0	5	7	1598_166	1542_166	56_0	NA	0
PH-739	phasi_NO578	Chr06	28340676-28340927	2267	ND	251	6_6	1_1	7	6	307_78	243_63	64_15	NA	0
PH-770	-	Chr07	1175885-1176136	..	ND	251	3_2	No	2	2	52_9	52_9	0_0	Rep_LOC_Os06g29260,Rep_LOC_(	3
PH-785	phasi_NO582	Chr07	5715758-5716009	4539622	-74	325	4_4	2_2	6	4	650_83	144_25	506_58	NA	0
PH-786	-	Chr07	5719821-5720072	3812	-74	325	5_5	2_2	7	4	429_58	294_43	135_15	NA	0
PH-787	-	Chr07	5723115-5723366	3043	-32	283	3_3	No	3	2	430_100	430_100	0_0	NA	0
PH-788	-	Chr07	5728432-5728683	5066	-74	325	4_4	1_1	5	3	219_28	154_23	65_5	NA	0
PH-789	phasi_NO584	Chr07	5730345-5730596	1662	-32	283	3_1	No	1	3	236_18	236_18	0_0	NA	0
PH-790	-	Chr07	5732248-5732499	1652	-32	283	8_4	No	4	6	122_14	122_14	0_0	NA	0
PH-791	-	Chr07	5733596-5733847	1097	-32	283	5_3	1_0	3	4	243_30	232_30	11_0	NA	0
PH-792	phasi_NO594	Chr07	6361878-6362129	628031	-179	430	3_2	1_1	3	4	999_114	293_48	706_66	NA	0
PH-793	-	Chr07	6680393-6680644	318264	-116	367	5_5	1_1	6	5	3003_185	2990_183	13_2	NA	0
PH-794	-	Chr07	6693472-6693723	12828	-32	283	3_2	No	2	2	102_8	102_8	0_0	NA	0
PH-795	phasi_NO598	Chr07	6720154-6720405	26431	-32	283	9_6	No	6	7	276_32	276_32	0_0	LOC_Os07g12030.1	8
PH-796	-	Chr07	6721394-6721645	989	-32	283	7_6	No	6	6	355_53	355_53	0_0	NA	0
PH-797	-	Chr07	6868397-6868648	146752	ND	251	5_5	No	5	4	916_94	916_94	0_0	NA	0
PH-798	phasi_NO601	Chr07	6869453-6869704	805	ND	251	5_3	No	3	5	318_24	318_24	0_0	NA	0
PH-799	phasi_NO603	Chr07	6873529-6873780	3825	ND	251	4_3	No	3	4	124_10	124_10	0_0	NA	0
PH-800	phasi_NO606	Chr07	6900911-6901162	27131	ND	251	6_5	2_0	5	7	389_33	340_33	49_0	NA	0
PH-801	phasi_NO607	Chr07	6907632-6907883	6470	ND	251	3_2	No	2	2	700_49	700_49	0_0	NA	0
PH-802	-	Chr07	6912252-6912503	4369	ND	251	3_0	No	0	3	360_0	360_0	0_0	NA	0
PH-803	phasi_NO609	Chr07	6920579-6920830	8076	2	253	7_5	No	5	5	329_29	329_29	0_0	NA	0
PH-804	phasi_NO610	Chr07	6922208-6922459	1378	ND	251	4_2	No	2	4	666_43	666_43	0_0	NA	0
PH-805	phasi_NO611	Chr07	6926871-6927122	4412	ND	251	2_2	No	2	2	107_15	107_15	0_0	NA	0
PH-806	-	Chr07	6934868-6935119	7746	ND	251	6_3	No	3	5	120_14	120_14	0_0	LOC_Os07g12304.1	6
PH-807	-	Chr07	6938028-6938279	2909	ND	251	3_3	No	3	3	1001_97	1001_97	0_0	LOC_Os07g12310	3
PH-808	-	Chr07	6938794-6939045	515	ND	251	10_7	3_3	10	12	1228_125	829_66	399_59	LOC_Os07g12310.1	10
PH-809	-	Chr07	6941454-6941705	2409	ND	251	3_2	No	2	2	130_12	130_12	0_0	NA	0
PH-810	-	Chr07	6943102-6943353	1397	ND	251	8_7	No	7	4	699_73	699_73	0_0	NA	0

Name	Alt_name	Chr	Positions (sRNAworkbench)	Dist_w/previous (bp)	MEME motif position (bp)	Size_w/MEME (bp)	Phased clusters nb (Ob_Og)	Unphased_clusters nb (Ob_Og)	Common clusters nb	Diff exp cluster nb	Total_reads nb (Ob_Og)	Total_phased_reads (Ob_Og)	Total_unphased_reads (Ob_Og)	Annotation_Pipeline	Annotated cluster nb
PH-811	-	Chr07	6975919-6976170	32566	ND	251	3_2	1_0	2	3	100_6	80_6	20_0	NA	0
PH-812	phasi_NO612	Chr07	6983096-6983347	6926	-53	304	5_3	No	3	4	638_41	638_41	0_0	NA	0
PH-813	-	Chr07	7032102-7032353	48755	202	453	4_2	13_13	15	4	317_217	81_8	236_209	LOC_Os02g08410.2,Rep_LOC_Os0	13
PH-814	phasi_NO617	Chr07	7037715-7037966	5362	-157	408	5_4	4_2	6	7	1911_208	295_44	1616_164	NA	0
PH-815	-	Chr07	7061406-7061657	23440	-137	388	3_0	No	0	3	37_0	37_0	0_0	NA	0
PH-816	phasi_NO619	Chr07	7069679-7069930	8022	-116	367	4_2	1_0	2	5	353_23	337_23	16_0	NA	0
PH-817	phasi_NO620	Chr07	7071798-7072049	1868	-53	304	6_3	2_2	5	7	155_15	94_8	61_7	NA	0
PH-818	phasi_NO621	Chr07	7073658-7073909	1609	-95	346	6_4	No	4	5	260_31	260_31	0_0	NA	0
PH-819	-	Chr07	8560589-8560840	1486680	-32	283	5_4	2_1	5	7	1099_85	458_41	641_44	NA	0
PH-820	phasi_NO624	Chr07	8601105-8601356	40265	-179	430	4_4	2_1	5	5	259_64	210_58	49_6	LOC_Os07g15000.1	4
PH-821	-	Chr07	8654114-8654365	52758	-95	346	3_2	1_1	3	4	132_10	85_6	47_4	NA	0
PH-822	-	Chr07	8663368-8663619	9003	-32	283	6_4	No	4	5	107_11	107_11	0_0	NA	0
PH-1006	-	Chr07	8677743-8677994	14124	ND	251	3_3	2_0	3	4	186_17	162_17	24_0	NA	0
PH-823	phasi_NO629	Chr07	8715913-8716164	37919	-116	367	6_5	2_2	7	7	522_71	217_29	305_42	NA	0
PH-824	-	Chr07	9032237-9032488	316073	-53	304	5_2	No	2	5	118_9	118_9	0_0	LOC_Os07g15590	5
PH-825	-	Chr07	9068833-9069084	36345	-53	304	4_4	No	4	2	126_14	126_14	0_0	NA	0
PH-826	phasi_NO633	Chr07	9444382-9444633	375298	ND	251	4_4	No	4	3	405_39	405_39	0_0	NA	0
PH-771	-	Chr07	13678059-13678310	4233426	ND	251	2_2	No	2	2	108_11	108_11	0_0	NA	0
PH-772	-	Chr07	13716857-13717108	38547	-116	367	3_3	No	3	3	139_11	139_11	0_0	NA	0
PH-773	phasi_NO641	Chr07	13735046-13735297	17938	-95	346	3_3	No	3	2	86_21	86_21	0_0	NA	0
PH-774	phasi_NO644	Chr07	14867734-14867985	1132437	ND	251	2_2	1_1	3	0	29_33	21_27	8_6	Rep_LOC_Os12g31040,Rep_LOC_	3
PH-775	phasi_NO151	Chr07	15007694-15007945	139709	-32	283	6_6	4_0	6	10	493_106	423_106	70_0	NA	0
PH-776	phasi_NO647	Chr07	15967651-15967902	959706	ND	251	8_6	3_3	9	8	2459_321	2417_313	42_8	RF01058;MIR806;AC113249.2/41	1
PH-777	-	Chr07	16039343-16039594	71441	-32	283	4_3	No	3	3	79_10	79_10	0_0	NA	0
PH-778	phasi_NO649	Chr07	16042379-16042630	2785	-74	325	5_4	1_0	4	5	158_29	138_29	20_0	NA	0
PH-779	-	Chr07	16046390-16046641	3760	-95	346	8_7	1_1	8	4	2117_200	2102_197	15_3	NA	0
PH-780	phasi_NO652	Chr07	16115091-16115342	68450	-96	347	4_3	2_2	5	5	491_78	312_48	179_30	NA	0
PH-781	-	Chr07	17296961-17297212	1181619	ND	251	3_1	No	1	3	335_35	335_35	0_0	NA	0
PH-782	-	Chr07	17336349-17336600	39137	ND	251	3_3	No	3	1	73_14	73_14	0_0	NA	0
PH-783	phasi_NO655	Chr07	17340876-17341127	4276	ND	251	5_4	1_0	4	5	151_18	128_18	23_0	NA	0
PH-784	-	Chr07	29697104-29697355	12355977	ND	251	12_12	3_3	15	0	1290_1512	125_121	1165_1391	RF00177;SSU_rRNA_bacteria;CU9	15
PH-827	-	Chr08	1002-1253	..	ND	251	20_20	10_10	30	1	1465_1768	972_1139	493_629	Rep_ORSiTRTM00000003,RF00177	30
PH-828	-	Chr08	1314-1565	61	ND	251	12_12	5_5	17	0	1335_1561	450_548	885_1013	RF00177;SSU_rRNA_bacteria;CU9	17
PH-852	phasi_NO657	Chr08	2440008-2440259	2438443	ND	251	3_3	1_0	3	4	348_38	334_38	14_0	NA	0
PH-853	-	Chr08	2441706-2441957	1447	-74	325	5_4	No	4	2	230_30	230_30	0_0	NA	0
PH-854	phasi_NO659	Chr08	2456738-2456989	14781	ND	251	4_4	No	4	4	216_27	216_27	0_0	NA	0
PH-855	-	Chr08	2460296-2460547	3307	-74	325	5_4	3_2	6	6	274_50	226_44	48_6	NA	0
PH-856	-	Chr08	2461218-2461469	671	-74	325	3_2	1_0	2	3	70_8	51_8	19_0	NA	0
PH-857	-	Chr08	2462464-2462715	995	ND	251	6_4	No	4	5	582_98	582_98	0_0	NA	0
PH-858	-	Chr08	2672302-2672553	209587	ND	251	4_2	1_1	3	5	155_15	127_12	28_3	NA	0
PH-859	phasi_NO670	Chr08	2673785-2674036	1232	ND	251	5_3	1_0	3	6	450_74	440_74	10_0	NA	0
PH-860	phasi_NO677	Chr08	2693471-2693722	19435	ND	251	5_3	No	3	3	103_9	103_9	0_0	NA	0
PH-861	phasi_NO678	Chr08	5732758-5733009	3039036	ND	251	3_2	No	2	3	182_21	182_21	0_0	NA	0
PH-862	phasi_NO679	Chr08	5743703-5743954	10694	ND	251	7_3	No	3	7	1486_116	1486_116	0_0	NA	0
PH-863	-	Chr08	7640428-7640679	1896474	ND	251	3_3	3_3	6	0	42_46	20_17	22_29	Rep_LOC_Os12g39700,Rep_LOC_	6
PH-829	-	Chr08	16727595-16727846	9086916	-221	472	4_1	2_1	2	6	130_6	81_4	49_2	NA	0
PH-830	phasi_NO687	Chr08	16735923-16736174	8077	ND	251	7_6	No	6	4	795_136	795_136	0_0	NA	0
PH-831	-	Chr08	17067056-17067307	330882	-116	367	3_1	No	1	3	62_4	62_4	0_0	NA	0
PH-832	phasi_NO692	Chr08	17084824-17085075	17517	-205	456	3_2	4_3	5	6	160_18	58_7	102_11	Rep_LOC_Os08g28030	2
PH-833	-	Chr08	17109669-17109920	24594	ND	251	6_5	No	5	4	979_129	979_129	0_0	NA	0
PH-834	phasi_NO702	Chr08	17161312-17161563	51392	-32	283	6_2	No	2	6	496_9	496_9	0_0	NA	0
PH-835	phasi_NO706	Chr08	17178006-17178257	16443	-74	325	3_3	No	3	3	1348_10	1348_10	0_0	NA	0
PH-836	-	Chr08	18585603-18585854	1407346	ND	251	6_4	1_1	5	5	166_25	156_18	10_7	NA	0
PH-837	phasi_NO710	Chr08	18587993-18588244	2139	ND	251	5_4	1_0	4	4	156_24	145_24	11_0	NA	0
PH-838	phasi_NO711	Chr08	18606747-18606998	18503	-203	454	4_2	4_3	5	6	457_79	129_29	328_50	NA	0
PH-839	phasi_NO712	Chr08	18613910-18614161	6912	-51	302	5_4	1_1	5	4	788_160	545_119	243_41	NA	0
PH-840	-	Chr08	18615536-18615787	1375	-74	325	3_3	2_2	5	3	204_34	83_21	121_13	NA	0
PH-841	-	Chr08	18715329-18715580	99542	419	670	3_1	No	1	3	90_4	90_4	0_0	NA	0
PH-842	phasi_NO715	Chr08	18719611-18719862	4031	-116	367	3_2	1_1	3	4	670_97	644_95	26_2	NA	0
PH-843	phasi_NO717	Chr08	18780530-18780781	60668	ND	251	4_3	1_0	3	2	59_15	49_15	10_0	NA	0
PH-844	phasi_NO719	Chr08	18819941-18820192	39160	ND	251	3_3	No	3	1	91_14	91_14	0_0	NA	0
PH-845	-	Chr08	18827731-18827982	7539	ND	251	10_7	2_0	7	11	1145_151	1122_151	23_0	LOC_Os08g30560	12
PH-846	phasi_NO723	Chr08	18834839-18835090	6857	ND	251	6_4	1_1	5	4	160_22	140_17	20_5	LOC_Os08g30580	5
PH-847	-	Chr08	18848176-18848427	13086	ND	251	4_4	No	4	4	383_36	383_36	0_0	NA	0
PH-848	-	Chr08	18934362-18934613	85935	ND	251	8_5	No	5	6	282_24	282_24	0_0	Rep_ORSgTEMT02400075	1
PH-849	-	Chr08	18944781-18945032	10168	ND	251	7_6	No	6	4	217_49	217_49	0_0	NA	0
PH-850	phasi_NO725	Chr08	18949275-18949526	4243	ND	251	4_3	No	3	3	167_44	167_44	0_0	NA	0
PH-851	phasi_NO726	Chr08	18951994-18952245	2468	ND	251	6_6	No	6	3	473_116	473_116	0_0	NA	0
PH-866	-	Chr09	6627774-6628025	..	ND	251	3_1	1_1	2	2	42_6	34_3	8_3	NA	0
PH-867	-	Chr09	6630076-6630327	2051	ND	251	4_3	No	3	4	95_7	95_7	0_0	NA	0
PH-868	phasi_NO731	Chr09	6634215-6634466	3888	ND	251	10_7	No	7	8	296_34	261_34	35_0	NA	0
PH-869	phasi_NO727	Chr09	6656392-6656643	21926	ND	251	7_4	No	4	6	249_60	249_60	0_0	Rep_LOC_Os09g11870	4
PH-870	-	Chr09	6657697-6657948	1054	ND	251	4_4	1_1	5	2	733_198	691_194	42_4	NA	0
PH-871	-	Chr09	6661605-6661856	3657	ND	251	3_3	No	3	2	101_26	101_26	0_0	NA	0
PH-872	phasi_NO732	Chr09	6678654-6678905	16798	ND	251	2_2	No	2	2	41_8	41_8	0_0	LOC_Os09g11890.1	2
PH-873	phasi_NO733	Chr09	6712656-6712907	33751	431	682	7_3	3_0	3	10	289_23	250_23	39_0	NA	0
PH-874	phasi_NO735	Chr09	6718272-6718523	5365	ND	251	4_0	No	0	4	73_0	73_0	0_0	NA	0
PH-875	-	Chr09	6740133-6740384	21610	ND	251	4_2	No	2	4	316_22	316_22	0_0	NA	0
PH-876	-	Chr09	6798936-6799187	58552	ND	251	6_5	No	5	3	438_78	438_78	0_0	NA	0
PH-877	-	Chr09	6824222-6824473	25035	ND	251	5_4	No	4	5	161_26	161_26	0_0	NA	0
PH-878	phasi_NO741	Chr09	6928683-6928934	104210	ND	251	3_2	1_1	3	3	1521_118	1511_116	10_2	Rep_LOC_Os09g12220	4
PH-879	-	Chr09	8602257-8602508	1673323	ND	251	2_2	3_3	5	0	79_71	43_40	36_31	Rep_LOC_Os12g12080,Rep_ORSg	5
PH-880	-	Chr09	8761026-8761277	158518	-32	283	4_4	No	4	1	55_19	55_19	0_0	NA	0
PH-881	-	Chr09	8764656-8764907	3379	-74	325	5_5	No	5	4	262_42	262_42	0_0	NA	0
PH-882	-	Chr09	8777110-8777361	12203	-74	325	4_4	4_1	5	5	208_29	124_20	84_9	NA	0
PH-883	-	Chr09	8784966-8785217	7605	-72	323	3_2	1_0	2	3	127_9	113_9	14_0	NA	0
PH-884	phasi_NO747	Chr09	8887894-8888145	102677	ND	251	4_3	1_1	4	4	112_13	42_3	70_10	NA	0
PH-885	-	Chr09	8896985-8897236	8840	ND	251	3_2	No	2	2	70_21	70_21	0_0	NA	0
PH-886	-	Chr09	8910694-8910945	13458	-74	325	3_3	No	3	3	707_145	707_145	0_0	NA	0
PH-887	-	Chr09	8913024-8913275	2079	ND	251	6_4	1_0	4	7	441_56	427_56	14_0	NA	0
PH-888	-	Chr09	8961821-8962072	48546	-32	283	3_2	No	2	3	97_12	97_12	0_0	NA	0
PH-889	-	Chr09	8977609-8977860	15537	402	653	9_7	No	7	7	401_63	144_25	257_38	NA	0
PH-890	phasi_NO753	Chr09	8978064-8978315	204	-53	304	5_4	No	4	4	257_38	257_38	0_0	NA	0
PH-891	-	Chr09	9003950-9004201	25635	-11	262	5_5	No	5	2	84_14	84_14	0_0	NA	0
PH-892	-	Chr09	9007448-9007699	3247	-32	283	4_4	No	4	3	188_32	188_32	0_0	NA	0
PH-893	-	Chr09	9144018-9144269	136319	-95	346	3_3	No	3	2	209_13	209_13	0_0	NA	0
PH-894	-	Chr09	9174506-9174757	30237	-263	514	3_3	No	3	2	97_23	97_23	0_0	NA	0
PH-895	phasi_NO765	Chr09	9194068-9194319	19311	-53	304	7_5	No	5	5	1747_281	1747_281	0_0	NA	0
PH-1015	phasi_NO765	Chr09	9194437-9194688	118	-422	673	10_8	1_1	9	8	2034_355	234_71	1800_284	NA	0
PH-896	-	Chr09	9197745-9197996	3057	-32	283	3_2	No	2	3	117_12	117_12	0_0	NA	0
PH-897	phasi_NO766	Chr09	9277545-9277796	79549	-32	283	9_8	No	8	5	587_117	587_117	0_0	NA	0
PH-898	-	Chr09	9284592-9284843	6796	ND	251	3_2	2_2	4	5	1395_96	334_23	1061_73	NA	0
PH-899	phasi_NO767	Chr09	9291311-9291562	6468	-11	262	5_4	1_1	5	4	123_20	108_18	15_2	NA	0
PH-900	-	Chr09	9324447-9324698	32885	-225	476	6_4	1_0	4	7	262_34	236_34	26_0	NA	0
PH-864	-	Chr09	16672452-16672703	7347754	ND	251	3_3	1_1	4	0	78_75	16_24	62_51	Rep_LOC_Os12g12080,Rep_ORSg	4
PH-865	-	Chr09	20974306-20974557	4301603	ND	251	2_2	1_1	3	0	27_33	19_27	8_6	Rep_ORSgTEMT00500708,Rep_LO	3
PH-33	-	Chr10	1003-1254	..	ND	251	20_20	10_10	30	1	1465_1768	558_680	907_1088	Rep_ORSiTRTM00000003,RF00177	30
PH-54	-	Chr10	3676172-3676423	3674918	-53	304	5_5	2_1	6	5	644_60	602_58	42_2	NA	0
PH-55	-	Chr10	3679423-3679674	3000	-32	283	3_2	No	2	1	47_9	47_9	0_0	NA	0
PH-56	-	Chr10	8661379-8661630	4981705	ND	251	2_2	1_1	3	0	29_33	21_27	8_6	Rep_LOC_Os12g31040,Rep_LOC_	3
PH-34	-	Chr10	13965385-13965636	5303755	-116	367	4_4	2_1	5	5	432_28	389_24	43_4	LOC_Os10g26740,LOC_Os10g267	4
PH-35	-	Chr10	13966455-13966706	819	-74	325	4_4	1_0	4	4	2337_156	2320_156	17_0	LOC_Os10g26740.1	1
PH-36	-	Chr10	14038841-14039092	72135	ND	251	4_3	2_1	4	5	474_24	418_21	56_3	LOC_Os10g26800.1,LOC_Os10g26	6
PH-37	-	Chr10	14071287-14071538	32195	ND	251	4_3	1_0	3	3	127_23	105_23	22_0	NA	0

Name	Alt_name	Chr	Positions (sRNAworkbench)	Dist_w/previous (bp)	MEME motif position (bp)	Size_w/MEME (bp)	Phased clusters nb (Ob_Og)	Unphased_clusters nb (Ob_Og)	Common clusters nb	Diff exp cluster nb	Total_reads nb (Ob_Og)	Total_phased_reads (Ob_Og)	Total_unphased_reads (Ob_Og)	Annotation_Pipeline	Annotated cluster nb
PH-38	-	Chr10	14074622-14074873	3084	ND	251	5_3	3_2	5	7	287_22	97_14	190_8	NA	0
PH-39	phasi_NO82	Chr10	14107994-14108245	33121	ND	251	5_4	1_0	4	5	282_41	265_41	17_0	NA	0
PH-40	-	Chr10	14121873-14122124	13628	ND	251	4_1	1_0	1	5	162_2	139_2	23_0	NA	0
PH-41	-	Chr10	14125305-14125556	3181	ND	251	3_2	No	2	2	98_4	98_4	0_0	Rep_LOC_Os10g26870	3
PH-42	-	Chr10	14139026-14139277	13470	ND	251	2_1	No	1	2	70_5	70_5	0_0	NA	0
PH-43	-	Chr10	14141814-14142065	2537	ND	251	6_5	2_1	6	5	199_34	139_30	60_4	LOC_Os10g26890.1,LOC_Os10g26	8
PH-44	phasi_NO334	Chr10	14147770-14148021	5705	ND	251	4_2	No	2	3	172_7	172_7	0_0	NA	0
PH-45	-	Chr10	14191490-14191741	43469	ND	251	3_3	No	3	2	50_8	50_8	0_0	NA	0
PH-46	-	Chr10	14193352-14193603	1611	ND	251	6_4	No	4	4	110_15	110_15	0_0	LOC_Os01g54080	1
PH-47	-	Chr10	14208193-14208444	14590	ND	251	7_6	2_1	7	8	1752_123	1671_120	81_3	NA	0
PH-48	phasi_NO796	Chr10	14219014-14219265	10570	ND	251	2_2	No	2	2	58_9	58_9	0_0	NA	0
PH-49	-	Chr10	14220262-14220513	997	ND	251	3_2	No	2	2	131_22	131_22	0_0	NA	0
PH-50	-	Chr10	14227481-14227732	6968	ND	251	5_3	No	3	5	315_29	315_29	0_0	LOC_Os09g02010	1
PH-51	-	Chr10	16607196-16607447	2379464	ND	251	4_4	9_9	13	0	320_302	82_76	238_226	osa-MIR819,osa-MIR806,RF01058	13
PH-52	-	Chr10	18732260-18732511	2124813	ND	251	3_3	3_3	6	0	92_84	16_24	76_60	Rep_LOC_Os09g19670,Rep_LOC_	6
PH-53	-	Chr10	20743570-20743821	2011059	208	459	2_2	3_3	5	0	110_86	20_18	90_68	Rep_ORSgTEMT00901206,Rep_LO	5
PH-73		Chr11	1657240-1657491	..	ND	251	2_2	1_1	3	0	25_43	17_37	8_6	Rep_LOC_Os12g31040,Rep_LOC_	3
PH-148	phasi_NO799	Chr11	3322065-3322316	1664574	ND	251	10_9	2_1	10	12	4923_575	4872_573	51_2	NA	0
PH-149	phasi_NO800	Chr11	7813071-7813322	4490755	-74	325	4_4	1_1	5	5	508_111	431_93	77_18	NA	0
PH-150	phasi_NO804	Chr11	9201226-9201477	1387904	ND	251	3_3	No	3	2	81_7	81_7	0_0	NA	0
PH-151	-	Chr11	9238574-9238825	37097	ND	251	5_4	No	4	5	495_33	495_33	0_0	NA	0
PH-152	phasi_NO807	Chr11	9292531-9292782	53706	ND	251	3_3	No	3	3	614_74	614_74	0_0	NA	0
PH-153	-	Chr11	9295651-9295902	2869	ND	251	5_4	No	4	5	144_14	144_14	0_0	NA	0
PH-154	phasi_NO810	Chr11	9332243-9332494	36341	ND	251	5_4	No	4	5	221_11	221_11	0_0	NA	0
PH-155	phasi_NO811	Chr11	9445014-9445265	112520	ND	251	4_1	2_1	2	5	148_56	102_3	46_53	osa-MIR819	2
PH-156	-	Chr11	9447125-9447376	1860	-53	304	3_2	No	2	2	168_10	168_10	0_0	NA	0
PH-157	phasi_NO812	Chr11	9449872-9450123	2496	-221	472	6_2	5_2	4	11	504_19	229_7	275_12	NA	0
PH-158	-	Chr11	9451147-9451398	1024	-169	420	3_0	1_1	1	4	289_3	259_0	30_3	LOC_Os07g44860.1	1
PH-159	-	Chr11	9459724-9459975	8326	-53	304	6_5	1_0	5	6	445_27	434_27	11_0	NA	0
PH-160	-	Chr11	9570358-9570609	110383	ND	251	4_2	No	2	3	83_6	83_6	0_0	NA	0
PH-161	-	Chr11	9578718-9578969	8109	-97	348	3_2	No	2	3	98_11	98_11	0_0	osa-MIR5527	1
PH-162	-	Chr11	9589947-9590198	10978	ND	251	2_1	No	1	2	50_7	34_7	16_0	osa-MIR5527	1
PH-163	phasi_NO818	Chr11	9835933-9836184	245735	ND	251	4_2	1_1	3	5	553_52	202_20	351_32	NA	0
PH-164	-	Chr11	9845876-9846127	9692	ND	251	6_6	No	6	3	188_17	188_17	0_0	NA	0
PH-165	-	Chr11	9851977-9852228	5850	ND	251	3_2	No	2	3	299_8	299_8	0_0	LOC_Os11g17650	2
PH-166	-	Chr11	9858435-9858686	6207	ND	251	3_3	No	3	3	246_36	246_36	0_0	NA	0
PH-167	-	Chr11	9933415-9933666	74729	ND	251	2_2	No	2	2	131_16	131_16	0_0	NA	0
PH-168	-	Chr11	9936588-9936839	2922	ND	251	3_0	No	0	3	43_0	43_0	0_0	NA	0
PH-169	-	Chr11	9940967-9941218	4128	ND	251	3_1	No	1	3	56_2	56_2	0_0	NA	0
PH-170	-	Chr11	9964167-9964418	22949	ND	251	3_0	No	0	3	76_0	76_0	0_0	NA	0
PH-57	-	Chr11	10007254-10007505	42836	ND	251	3_0	No	0	3	118_0	118_0	0_0	NA	0
PH-58	-	Chr11	10028547-10028798	21042	ND	251	3_1	No	1	3	73_4	73_4	0_0	NA	0
PH-59	-	Chr11	15379085-15379336	5350287	-137	388	4_3	1_1	4	4	302_16	296_10	6_6	RF01062;MIR812;AACV01019908.	1
PH-60	phasi_NO828	Chr11	15763480-15763731	384144	-74	325	7_7	1_1	8	6	811_181	774_176	37_5	NA	0
PH-61	-	Chr11	15861177-15861428	97446	-74	325	10_5	1_1	6	7	931_81	890_75	41_6	NA	0
PH-62	phasi_NO831	Chr11	15863945-15864196	2517	-53	304	5_5	No	5	5	223_25	205_23	18_2	NA	0
PH-905	phasi_NO830	Chr11	15868760-15869011	4564	-32	283	11_9	1_1	10	8	427_115	386_109	41_6	NA	0
PH-63	phasi_NO830	Chr11	15868802-15869053	209	-74	325	12_10	1_1	11	9	1474_295	1082_180	392_115	NA	0
PH-64	phasi_NO831	Chr11	15870856-15871107	1803	-137	388	4_3	2_1	4	4	101_14	77_12	24_2	NA	0
PH-65	-	Chr11	15871955-15872206	848	ND	251	5_3	No	3	3	104_13	104_13	0_0	NA	0
PH-66	phasi_NO834	Chr11	16088725-16088976	216519	-225	476	5_3	5_5	8	8	566_100	376_66	190_34	NA	0
PH-67	phasi_NO842	Chr11	16520278-16520529	431302	ND	251	4_2	No	2	4	166_18	166_18	0_0	NA	0
PH-68	phasi_NO843	Chr11	16527412-16527663	6883	ND	251	3_2	No	2	3	131_20	131_20	0_0	NA	0
PH-69	phasi_NO840	Chr11	16531783-16532034	4120	ND	251	9_9	No	9	8	1678_275	1678_275	0_0	NA	0
PH-70	phasi_NO797	Chr11	16543023-16543274	10989	ND	251	5_3	No	3	3	87_9	87_9	0_0	NA	0
PH-71	-	Chr11	16559289-16559540	16015	ND	251	3_3	No	3	3	70_12	70_12	0_0	NA	0
PH-72	-	Chr11	16566171-16566422	6631	ND	251	3_2	No	2	3	97_23	97_23	0_0	NA	0
PH-74	phasi_NO846	Chr11	16583536-16583787	17114	ND	251	4_3	No	3	4	92_8	92_8	0_0	NA	0
PH-75	-	Chr11	16599291-16599542	15504	ND	251	4_3	1_1	4	3	131_18	89_13	42_5	NA	0
PH-76	phasi_NO849	Chr11	16653092-16653343	53550	ND	251	4_4	No	4	4	2662_333	2662_333	0_0	NA	0
PH-77	-	Chr11	16670420-16670671	17077	ND	251	2_2	3_3	5	0	43_53	20_35	23_18	Rep_LOC_Os12g31040,Rep_LOC_	5
PH-78	-	Chr11	19501355-19501606	2830684	ND	251	3_3	No	3	1	974_78	974_78	0_0	NA	0
PH-79	-	Chr11	19502297-19502548	691	ND	251	3_3	1_1	4	2	1667_151	71_17	1596_134	NA	0
PH-80	phasi_NO869	Chr11	22978397-22978648	3475849	-280	531	12_9	5_3	12	12	891_85	499_62	392_23	NA	0
PH-81	-	Chr11	22988752-22989003	10104	ND	251	3_2	No	2	2	76_7	76_7	0_0	NA	0
PH-82	-	Chr11	22990366-22990617	1363	ND	251	5_4	No	4	5	164_13	164_13	0_0	NA	0
PH-83	-	Chr11	22993020-22993271	2403	ND	251	12_10	5_3	13	13	2867_342	2721_335	146_7	NA	0
PH-84	phasi_NO864	Chr11	23001541-23001792	8270	ND	251	8_6	No	6	7	474_71	474_71	0_0	NA	0
PH-85	phasi_NO861	Chr11	23005331-23005582	3539	165	416	5_0	No	0	5	104_0	104_0	0_0	NA	0
PH-86	phasi_NO862	Chr11	23008631-23008882	3049	ND	251	3_0	No	0	3	79_0	79_0	0_0	NA	0
PH-87	phasi_NO856	Chr11	23035667-23035918	26785	ND	251	4_1	No	1	3	75_3	75_3	0_0	NA	0
PH-88	phasi_NO854	Chr11	23043206-23043457	7288	ND	251	5_4	No	4	4	176_20	176_20	0_0	NA	0
PH-89	-	Chr11	23044310-23044561	853	ND	251	4_3	No	3	1	60_14	60_14	0_0	NA	0
PH-90	phasi_NO852	Chr11	23054510-23054761	9949	ND	251	5_3	2_1	4	7	350_45	303_39	47_6	NA	0
PH-91	-	Chr11	23056313-23056564	1552	ND	251	4_3	No	3	3	140_23	140_23	0_0	NA	0
PH-92	-	Chr11	24242636-24242887	1186072	-263	514	4_2	1_0	2	3	143_8	112_8	31_0	NA	0
PH-93	-	Chr11	24248173-24248424	5286	ND	251	3_3	No	3	3	444_83	444_83	0_0	NA	0
PH-94	phasi_NO901	Chr11	24335644-24335895	87220	ND	251	8_8	1_1	9	7	344_38	304_36	40_2	NA	0
PH-95	-	Chr11	24341060-24341311	5165	-116	367	3_2	No	2	2	71_17	71_17	0_0	NA	0
PH-96	-	Chr11	24345228-24345479	3917	ND	251	5_3	No	3	4	182_19	182_19	0_0	NA	0
PH-97	-	Chr11	24347135-24347386	1656	-74	325	3_3	No	3	3	260_36	260_36	0_0	NA	0
PH-98	-	Chr11	24404640-24404891	57254	ND	251	4_3	No	3	3	188_41	188_41	0_0	NA	0
PH-99	phasi_NO784	Chr11	24406940-24407191	2049	-3	254	6_4	1_0	4	5	455_72	441_72	14_0	NA	0
PH-100	-	Chr11	24411199-24411450	4008	ND	251	3_3	No	3	2	172_16	172_16	0_0	LOC_Os03g06710	1
PH-101	-	Chr11	24412137-24412388	687	ND	251	6_6	1_0	6	5	587_84	570_84	17_0	NA	0
PH-102	phasi_NO749	Chr11	24456912-24457163	44524	ND	251	3_1	No	1	3	107_3	107_3	0_0	NA	0
PH-103	-	Chr11	24459949-24460200	2786	ND	251	4_3	No	3	2	60_7	60_7	0_0	NA	0
PH-911	phasi_NO874	Chr11	24625448-24625699	165248	ND	251	4_4	3_0	4	6	378_72	310_72	68_0	NA	0
PH-104	-	Chr11	24677987-24678238	52288	-53	304	3_1	No	1	3	126_12	126_12	0_0	NA	0
PH-105	-	Chr11	24680939-24681190	2701	-32	283	4_1	No	1	3	81_3	81_3	0_0	NA	0
PH-106	-	Chr11	24682575-24682826	1385	-32	283	6_5	1_1	6	7	288_41	267_37	21_4	NA	0
PH-107	-	Chr11	24683369-24683620	543	-137	388	6_4	No	4	5	156_26	156_26	0_0	LOC_Os03g60890	1
PH-108	-	Chr11	24702226-24702477	18606	-95	346	3_3	No	3	2	210_28	210_28	0_0	NA	0
PH-109	phasi_NO879	Chr11	24703729-24703980	1252	-318	569	6_4	5_5	9	7	1582_268	667_113	915_155	NA	0
PH-110	-	Chr11	24707824-24708075	3844	-32	283	8_7	2_2	9	8	886_199	755_177	131_22	NA	0
PH-111	phasi_NO681	Chr11	24764635-24764886	56560	-32	283	4_3	No	3	3	667_58	667_58	0_0	NA	0
PH-112	phasi_NO682	Chr11	24765578-24765829	692	-53	304	3_0	No	0	3	60_0	60_0	0_0	NA	0
PH-113	-	Chr11	24767332-24767583	1503	-32	283	5_5	No	5	3	614_72	614_72	0_0	NA	0
PH-114	-	Chr11	24769604-24769855	2021	-53	304	3_1	No	1	2	52_5	52_5	0_0	NA	0
PH-115	phasi_NO683	Chr11	24770528-24770779	673	-53	304	5_5	2_1	6	4	237_67	155_43	82_24	NA	0
PH-116	-	Chr11	24771193-24771444	414	-11	262	4_3	No	3	4	95_9	95_9	0_0	NA	0
PH-117	-	Chr11	24773432-24773683	1988	-32	283	7_6	No	6	1	120_38	120_38	0_0	NA	0
PH-118	phasi_NO514	Chr11	24783557-24783808	9874	-74	325	7_6	1_0	6	6	412_74	392_74	20_0	NA	0
PH-119	-	Chr11	24789083-24789334	5275	-32	283	5_4	1_0	4	6	384_29	367_29	17_0	NA	0
PH-120	phasi_NO511	Chr11	24794119-24794370	4785	-116	367	6_5	No	5	6	296_26	296_26	0_0	NA	0
PH-121	phasi_NO510	Chr11	24796920-24797171	2550	-32	283	4_3	No	3	3	378_14	378_14	0_0	NA	0
PH-122	-	Chr11	24918751-24919002	121580	ND	251	3_2	No	2	2	462_18	462_18	0_0	NA	0
PH-123	phasi_NO509	Chr11	24919726-24919977	724	ND	251	5_5	No	5	2	341_43	341_43	0_0	NA	0
PH-124	-	Chr11	24921131-24921382	1154	ND	251	3_3	No	3	3	131_22	131_22	0_0	NA	0
PH-125	phasi_NO507	Chr11	24925845-24926096	4463	ND	251	10_8	No	8	9	572_133	572_133	0_0	NA	0
PH-126	phasi_NO30	Chr11	25027019-25027270	100923	-179	430	3_3	No	3	0	43_23	43_23	0_0	NA	0
PH-127	-	Chr11	25095394-25095645	68124	-95	346	5_5	No	5	4	796_132	796_132	0_0	NA	0
PH-128	-	Chr11	25112300-25112551	16655	-116	367	5_5	2_2	7	3	1009_118	977_112	32_6	NA	0
PH-129	-	Chr11	25118473-25118724	5922	-374	625	3_1	30_30	31	4	448_332	116_12	332_320	osa-MIR812,osa-MIR2123,osa-MIR	29

Name	Alt_name	Chr	Positions (sRNAworkbench)	Dist_w/previous (bp)	MEME motif position (bp)	Size_w/MEME (bp)	Phased clusters nb (Ob_Og)	Unphased_clusters nb (Ob_Og)	Common clusters nb	Diff exp cluster nb	Total_reads nb (Ob_Og)	Total_phased_reads (Ob_Og)	Total_unphased_reads (Ob_Og)	Annotation_Pipeline	Annotated cluster nb
PH-130	-	Chr11	25123267-25123518	4543	-116	367	4_4	1_0	4	4	210_35	200_35	10_0	NA	0
PH-131	phasi_NO889	Chr11	25126434-25126685	2916	-32	283	6_6	1_1	7	6	272_34	260_31	12_3	NA	0
PH-132	-	Chr11	25132240-25132491	5555	-53	304	6_6	No	6	5	1855_226	1855_226	0_0	NA	0
PH-133	phasi_NO895, pha	Chr11	25138032-25138283	5541	-32	283	10_10	No	10	8	317_53	317_53	0_0	NA	0
PH-134	phasi_NO890	Chr11	25140219-25140470	1936	ND	251	3_3	No	3	2	1460_146	1460_146	0_0	NA	0
PH-135	-	Chr11	25147966-25148217	7496	-32	283	3_3	No	3	3	204_50	204_50	0_0	NA	0
PH-136	phasi_NO911	Chr11	26322692-26322943	1174475	-32	283	10_8	No	8	8	1827_252	1827_252	0_0	NA	0
PH-137	phasi_NO912	Chr11	26324287-26324538	1344	-53	304	7_5	2_2	7	6	2109_356	2045_339	64_17	NA	0
PH-138	-	Chr11	26325000-26325251	462	-95	346	4_3	No	3	4	94_8	94_8	0_0	NA	0
PH-139	-	Chr11	26331335-26331586	6084	-53	304	4_3	No	3	3	137_28	137_28	0_0	NA	0
PH-140	phasi_NO913	Chr11	26332907-26333158	1321	-32	283	9_7	1_1	8	8	728_104	700_102	28_2	NA	0
PH-141	-	Chr11	26333942-26334193	784	-74	325	3_1	No	1	3	77_10	77_10	0_0	NA	0
PH-142	-	Chr11	26335925-26336176	1732	-32	283	7_4	1_1	5	6	215_22	203_20	12_2	NA	0
PH-143	phasi_NO914	Chr11	26338399-26338650	2223	-137	388	6_4	1_1	5	6	165_11	137_9	28_2	NA	0
PH-144	-	Chr11	26348012-26348263	9362	-32	283	5_4	No	4	3	122_29	122_29	0_0	NA	0
PH-145	-	Chr11	26348871-26349122	608	ND	251	5_3	No	3	5	272_28	272_28	0_0	NA	0
PH-146	-	Chr11	26385399-26385650	36277	390	641	7_6	1_1	7	6	1121_157	993_143	128_14	NA	0
PH-147	phasi_NO129	Chr11	26390581-26390832	4931	-263	514	3_3	No	3	1	68_12	68_12	0_0	NA	0
PH-358	-	Chr12	349888-350139	..	ND	251	3_3	3_3	6	1	134_179	33_49	101_130	osa-MIR819	6
PH-298	-	Chr12	2275036-2275287	1924897	ND	251	3_1	No	1	3	568_43	568_43	0_0	NA	0
PH-299	-	Chr12	2282098-2282349	6811	ND	251	3_1	1_0	1	3	77_2	58_2	19_0	Rep_LOC_Os12g05160	2
PH-300	-	Chr12	2284146-2284397	1797	ND	251	6_6	No	6	4	364_64	364_64	0_0	LOC_Os11g36780	1
PH-357	-	Chr12	3143543-3143794	859146	ND	251	4_4	No	4	3	170_25	170_25	0_0	NA	0
PH-359	-	Chr12	7558926-7559177	4415132	-137	388	3_3	1_1	4	2	378_46	45_8	333_38	NA	0
PH-360	-	Chr12	8088435-8088686	529258	-47	298	7_6	No	6	6	779_106	779_106	0_0	NA	0
PH-361	phasi_NO926	Chr12	8090253-8090504	1567	-158	409	4_3	2_1	4	6	144_13	114_11	30_2	NA	0
PH-362	-	Chr12	8126400-8126651	35896	ND	251	3_2	No	2	2	64_11	64_11	0_0	NA	0
PH-363	phasi_NO928	Chr12	8154176-8154427	27525	-221	472	4_3	No	3	2	74_25	74_25	0_0	LOC_Os12g14310.1	3
PH-364	phasi_NO929	Chr12	8180666-8180917	26239	-137	388	5_3	3_2	5	6	229_45	136_31	93_14	Rep_LOC_Os12g14360	8
PH-365	phasi_NO33	Chr12	8746171-8746422	565254	ND	251	6_6	1_1	7	4	434_111	421_108	13_3	NA	0
PH-366	-	Chr12	9190178-9190429	443756	-218	469	3_3	2_2	5	1	219_38	31_10	188_28	NA	0
PH-171	-	Chr12	10228672-10228923	1038243	-32	283	5_3	No	3	4	157_21	157_21	0_0	NA	0
PH-172	-	Chr12	10877954-10878205	649031	ND	251	2_2	1_1	3	0	25_43	17_37	8_6	Rep_LOC_Os12g31040,Rep_LOC_(	3
PH-173	-	Chr12	13755619-13755870	2877414	ND	251	4_2	No	2	3	61_12	61_12	0_0	NA	0
PH-174	-	Chr12	13970762-13971013	214892	ND	251	5_5	No	5	5	3303_362	3303_362	0_0	NA	0
PH-175	-	Chr12	14059344-14059595	88331	-53	304	3_1	No	1	3	390_8	390_8	0_0	NA	0
PH-176	phasi_NO923	Chr12	14178225-14178476	118630	ND	251	3_3	1_1	4	2	311_55	300_53	11_2	NA	0
PH-177	-	Chr12	14290466-14290717	111990	ND	251	2_2	No	2	0	22_5	22_5	0_0	NA	0
PH-178	-	Chr12	14459938-14460189	169221	-74	325	5_4	No	4	5	600_77	575_71	25_6	NA	0
PH-179	-	Chr12	14466213-14466464	6024	-53	304	5_3	No	3	5	542_68	520_68	22_0	NA	0
PH-180	-	Chr12	14546869-14547120	80405	ND	251	5_5	No	5	3	308_54	308_54	0_0	NA	0
PH-181	-	Chr12	14602391-14602642	55271	ND	251	5_5	No	5	3	199_45	199_45	0_0	NA	0
PH-182	-	Chr12	14603208-14603459	566	ND	251	4_4	1_0	4	4	209_22	194_22	15_0	NA	0
PH-183	-	Chr12	14677516-14677767	74057	-179	430	3_3	1_1	4	1	57_10	45_7	12_3	NA	0
PH-184	phasi_NO968	Chr12	14938313-14938564	260546	-74	325	4_3	No	3	4	226_24	226_24	0_0	NA	0
PH-185	phasi_NO967	Chr12	14944344-14944595	5780	-186	437	3_2	No	2	2	62_8	62_8	0_0	NA	0
PH-186	phasi_NO958	Chr12	15158603-15158854	214008	ND	251	9_6	No	6	8	257_25	105_9	152_16	NA	0
PH-187	phasi_NO957	Chr12	15164638-15164889	5784	-124	375	3_1	No	1	3	245_17	245_17	0_0	osa-MIR5506	1
PH-188	phasi_NO956	Chr12	15171729-15171980	6840	ND	251	9_7	No	7	6	297_32	255_26	42_6	NA	0
PH-189	phasi_NO955	Chr12	15176172-15176423	4192	-32	283	3_2	No	2	3	252_23	252_23	0_0	NA	0
PH-190	phasi_NO954	Chr12	15187039-15187290	10616	-53	304	6_5	No	5	5	1169_123	1169_123	0_0	NA	0
PH-191	-	Chr12	15206882-15207133	19592	-53	304	4_3	No	3	4	116_6	116_6	0_0	NA	0
PH-192	phasi_NO952	Chr12	15214289-15214540	7156	-74	325	6_3	2_2	5	5	324_37	287_32	37_5	NA	0
PH-193	-	Chr12	15215752-15216003	1212	-116	367	3_1	1_1	2	4	115_8	50_2	65_6	NA	0
PH-194	phasi_NO948	Chr12	15312743-15312994	96740	ND	251	5_3	No	3	4	152_26	152_26	0_0	NA	0
PH-195	phasi_NO949	Chr12	15317943-15318194	4949	ND	251	6_5	1_1	6	5	206_34	198_26	8_8	NA	0
PH-196	-	Chr12	15319413-15319664	1219	ND	251	4_3	No	3	2	98_20	98_20	0_0	NA	0
PH-197	-	Chr12	15533611-15533862	213947	452	703	4_1	No	1	3	423_3	0_0	423_3	NA	0
PH-198	-	Chr12	15535517-15535768	1655	-86	337	4_4	2_2	6	4	1266_149	417_42	849_107	NA	0
PH-199	-	Chr12	15543835-15544086	8067	ND	251	4_4	No	4	3	1528_95	1528_95	0_0	NA	0
PH-200	-	Chr12	15559156-15559407	15070	ND	251	4_2	No	2	4	132_10	16_0	116_10	NA	0
PH-201	-	Chr12	15561430-15561681	2023	ND	251	4_3	2_0	3	6	3771_426	3704_426	67_0	NA	0
PH-202	-	Chr12	15568353-15568604	6672	ND	251	7_4	2_0	4	8	555_35	504_35	51_0	NA	0
PH-203	-	Chr12	15600027-15600278	31423	-213	464	6_5	2_0	5	7	3795_430	3739_430	56_0	NA	0
PH-204	-	Chr12	15602322-15602573	2044	-53	304	6_3	No	3	6	166_14	150_14	16_0	NA	0
PH-205	-	Chr12	15617641-15617892	15068	-137	388	4_4	No	4	3	1528_95	263_17	1265_78	NA	0
PH-206	-	Chr12	15624445-15624696	6553	-137	388	4_1	1_1	2	4	467_10	423_3	44_7	NA	0
PH-207	-	Chr12	16210143-16210394	585447	440	691	5_5	No	5	3	201_34	201_34	0_0	NA	0
PH-208	-	Chr12	16259099-16259350	48705	-53	304	5_5	No	5	5	423_39	423_39	0_0	NA	0
PH-209	-	Chr12	16346492-16346743	87142	-32	283	5_5	No	5	4	502_117	502_117	0_0	NA	0
PH-210	-	Chr12	18022937-18023188	1676194	-74	325	7_7	1_0	7	5	315_74	301_74	14_0	NA	0
PH-211	phasi_NO978	Chr12	18039120-18039371	15932	ND	251	4_1	No	1	4	104_24	104_24	0_0	osa-MIR5488	3
PH-212	phasi_NO979	Chr12	18040350-18040601	979	-53	304	6_6	No	6	4	875_281	875_281	0_0	NA	0
PH-213	phasi_NO981	Chr12	18069786-18070037	29185	-53	304	3_3	No	3	0	36_17	36_17	0_0	NA	0
PH-214	phasi_NO983	Chr12	18076990-18077241	6953	-207	458	6_6	5_5	11	3	557_220	191_68	366_152	NA	0
PH-215	phasi_NO984	Chr12	18086718-18086969	9477	ND	251	7_7	2_2	9	4	462_153	436_142	26_11	NA	0
PH-216	-	Chr12	18102114-18102365	15145	ND	251	4_2	1_1	3	5	357_21	279_17	78_4	NA	0
PH-217	-	Chr12	18176607-18176858	74242	-116	367	5_3	1_1	4	4	122_17	86_12	36_5	NA	0
PH-218	phasi_NO990	Chr12	18179402-18179653	2544	-53	304	3_3	No	3	2	99_10	99_10	0_0	NA	0
PH-219	phasi_NO993	Chr12	18210483-18210734	30830	-116	367	5_3	1_0	3	6	317_26	306_26	11_0	NA	0
PH-220	-	Chr12	18264968-18265219	54234	-53	304	7_7	1_0	7	5	985_141	972_141	13_0	NA	0
PH-221	-	Chr12	18276918-18277169	11699	-53	304	4_2	No	2	3	114_8	114_8	0_0	NA	0
PH-222	phasi_NO996	Chr12	18280355-18280606	3186	-32	283	3_1	No	1	2	54_3	54_3	0_0	NA	0
PH-223	-	Chr12	18281938-18282189	1332	-179	430	4_2	1_1	3	4	133_15	115_12	18_3	NA	0
PH-224	phasi_NO997	Chr12	18287033-18287284	4844	-137	388	5_4	1_1	5	5	1628_212	1538_199	90_13	Rep_LOC_Os08g02000	1
PH-225	phasi_NO1000	Chr12	18292733-18292984	5449	-74	325	7_5	2_2	7	7	1857_264	1790_257	67_7	NA	0
PH-226	phasi_NO1006	Chr12	18622764-18623015	329780	ND	251	5_2	1_1	3	6	296_19	273_17	23_2	NA	0
PH-227	-	Chr12	18625840-18626091	2825	ND	251	3_2	No	2	3	126_4	126_4	0_0	NA	0
PH-228	phasi_NO1004	Chr12	18684752-18685003	58661	ND	251	3_2	No	2	2	161_29	161_29	0_0	NA	0
PH-229	-	Chr12	18703610-18703861	18607	ND	251	3_2	No	2	3	232_11	232_11	0_0	NA	0
PH-230	-	Chr12	19217380-19217631	513519	-53	304	3_1	No	1	3	95_2	95_2	0_0	NA	0
PH-231	-	Chr12	19219124-19219375	1493	-32	283	5_0	No	0	5	346_0	346_0	0_0	NA	0
PH-232	-	Chr12	19232468-19232719	13093	ND	251	6_1	No	1	6	691_10	691_10	0_0	NA	0
PH-233	-	Chr12	19238874-19239125	6155	-32	283	9_1	No	1	9	3297_3	3297_3	0_0	NA	0
PH-234	-	Chr12	19239794-19240045	669	-53	304	3_2	No	2	3	132_27	132_27	0_0	NA	0
PH-235	phasi_NO1008	Chr12	19251891-19252142	11846	-32	283	5_0	No	0	5	188_0	188_0	0_0	NA	0
PH-236	-	Chr12	19296155-19296406	44013	-32	283	3_0	0_1	0	3	71_23	71_0	0_23	NA	0
PH-237	-	Chr12	19317040-19317291	20634	-32	283	4_0	No	0	4	64_0	64_0	0_0	NA	0
PH-238	-	Chr12	21060675-21060926	1743384	-32	283	6_5	2_0	5	6	215_18	175_18	40_0	LOC_Os12g34690,LOC_Os12g346(	8
PH-239	-	Chr12	21061732-21061983	806	-32	283	4_3	No	3	4	81_13	81_13	0_0	NA	0
PH-240	-	Chr12	21063658-21063909	1675	-51	302	4_2	No	2	4	153_10	153_10	0_0	NA	0
PH-241	-	Chr12	21071305-21071556	7396	-74	325	5_4	2_1	5	6	178_34	135_32	43_2	Rep_LOC_Os12g34710	1
PH-242	-	Chr12	21072634-21072885	1078	-221	472	4_3	2_1	4	5	517_49	477_40	40_9	Rep_LOC_Os12g34710	6
PH-243	-	Chr12	21074132-21074383	1247	-74	325	3_2	No	2	3	94_10	94_10	0_0	Rep_LOC_Os12g34710	3
PH-244	-	Chr12	21114165-21114416	39782	-32	283	8_7	1_1	8	4	221_51	204_47	17_4	NA	0
PH-245	phasi_NO1023	Chr12	21118043-21118294	3627	-32	283	4_3	No	3	4	143_17	143_17	0_0	NA	0
PH-246	phasi_NO1024	Chr12	21119586-21119837	1292	-53	304	3_2	No	2	2	60_10	60_10	0_0	NA	0
PH-247	-	Chr12	21129563-21129814	9726	-11	262	7_4	No	4	6	158_12	158_12	0_0	NA	0
PH-248	phasi_NO1027	Chr12	21130727-21130978	913	-32	283	7_6	No	6	5	205_30	205_30	0_0	NA	0
PH-249	phasi_NO1028	Chr12	21132741-21132992	1763	-74	325	4_1	No	1	4	66_2	66_2	0_0	NA	0
PH-250	phasi_NO1029	Chr12	21156953-21157204	23961	-74	325	4_3	1_1	4	4	247_14	239_11	8_3	NA	0
PH-251	phasi_NO1032	Chr12	21163592-21163843	6388	ND	251	3_1	No	1	2	56_3	56_3	0_0	NA	0
PH-252	-	Chr12	21171687-21171938	7844	ND	251	4_4	No	4	3	195_44	195_44	0_0	NA	0
PH-253	-	Chr12	21173239-21173490	1301	-95	346	4_4	1_0	4	4	144_23	127_23	17_0	NA	0

Name	Alt_name	Chr	Positions (sRNAworkbench)	Dist_w/previous (bp)	MEME motif position (bp)	Size_w/MEME (bp)	Phased clusters nb (Ob_Og)	Unphased_clusters nb (Ob_Og)	Common clusters nb	Diff exp cluster nb	Total_reads nb (Ob_Og)	Total_phased_reads (Ob_Og)	Total_unphased_reads (Ob_Og)	Annotation_Pipeline	Annotated cluster nb
PH-254	phasi_NO1034	Chr12	21175560-21175811	2070	-200	451	4_3	2_0	3	5	125_13	102_13	23_0	NA	0
PH-255	-	Chr12	21178057-21178308	2246	398	649	3_3	2_2	5	4	2198_251	687_68	1511_183	NA	0
PH-256	-	Chr12	21182029-21182280	3721	-32	283	9_7	No	7	8	1412_235	1412_235	0_0	NA	0
PH-257	-	Chr12	21185743-21185994	3463	ND	251	3_1	No	1	2	47_2	47_2	0_0	NA	0
PH-258	-	Chr12	21187926-21188177	1932	ND	251	8_7	No	7	6	2196_247	2196_247	0_0	LOC_Os12g34810,LOC_Os12g348	4
PH-259	-	Chr12	21190312-21190563	2135	-326	577	3_2	5_1	3	7	250_16	61_4	189_12	NA	0
PH-260	-	Chr12	21193663-21193914	3100	ND	251	4_4	No	4	4	146_14	146_14	0_0	NA	0
PH-261	phasi_NO1038	Chr12	21194309-21194560	395	ND	251	4_4	No	4	4	211_20	211_20	0_0	NA	0
PH-262	-	Chr12	21716194-21716445	521634	-136	387	4_2	2_1	3	3	78_9	50_5	28_4	NA	0
PH-263	phasi_NO1040	Chr12	21733134-21733385	16689	-53	304	5_3	No	3	4	284_44	284_44	0_0	NA	0
PH-264	-	Chr12	21751341-21751592	17956	-116	367	4_2	1_0	2	5	123_5	104_5	19_0	NA	0
PH-265	phasi_NO141	Chr12	21768161-21768412	16569	-32	283	13_9	No	9	12	444_31	444_31	0_0	NA	0
PH-266	phasi_NO1044	Chr12	21781605-21781856	13193	-95	346	3_1	2_1	2	4	110_7	56_2	54_5	NA	0
PH-267	-	Chr12	21782359-21782610	503	-53	304	2_2	No	2	2	43_6	43_6	0_0	osa-MIR5530	1
PH-268	phasi_NO1045	Chr12	21791017-21791268	8407	-74	325	5_3	No	3	5	125_12	125_12	0_0	NA	0
PH-269	-	Chr12	21793081-21793332	1813	-32	283	5_3	No	3	5	164_11	164_11	0_0	NA	0
PH-270	phasi_NO1046	Chr12	21798178-21798429	4846	-74	325	6_4	No	4	4	118_14	118_14	0_0	NA	0
PH-271	phasi_NO1047	Chr12	21800908-21801159	2479	-32	283	9_6	1_0	6	9	492_47	474_47	18_0	NA	0
PH-272	-	Chr12	21817326-21817577	16167	-139	390	4_4	2_2	6	3	237_57	111_37	126_20	NA	0
PH-273	-	Chr12	21819659-21819910	2082	-32	283	9_7	1_1	8	9	1178_174	1157_169	21_5	NA	0
PH-274	-	Chr12	21868730-21868981	48820	-32	283	4_1	No	1	4	164_2	164_2	0_0	NA	0
PH-275	phasi_NO1050	Chr12	21878638-21878889	9657	-32	283	8_6	No	6	8	2239_191	2239_191	0_0	NA	0
PH-276	phasi_NO1052	Chr12	21892819-21893070	13930	-53	304	7_6	3_2	8	9	1769_162	1317_107	452_55	NA	0
PH-277	phasi_NO1052	Chr12	21907584-21907835	14514	-53	304	7_5	2_2	7	7	838_100	535_62	303_38	NA	0
PH-927	-	Chr12	21907836-21908087	1	-305	556	10_8	3_2	10	10	955_132	99_32	856_100	NA	0
PH-278	-	Chr12	21909231-21909482	1144	-53	304	5_3	1_1	4	6	608_63	487_50	121_13	NA	0
PH-279	-	Chr12	21912867-21913118	3385	-53	304	4_1	No	1	4	128_3	128_3	0_0	NA	0
PH-280	phasi_NO1054	Chr12	21913618-21913869	500	-32	283	6_4	2_1	5	8	685_36	591_30	94_6	NA	0
PH-281	-	Chr12	21923302-21923553	9433	-242	493	3_2	No	2	3	98_9	98_9	0_0	NA	0
PH-282	-	Chr12	22001016-22001267	77463	-32	283	4_0	No	0	4	146_0	146_0	0_0	NA	0
PH-283	phasi_NO1060	Chr12	22016881-22017132	15614	-32	283	7_5	No	5	7	444_135	444_135	0_0	NA	0
PH-284	phasi_NO1061	Chr12	22017835-22018086	703	-116	367	4_2	No	2	4	556_27	556_27	0_0	NA	0
PH-285	phasi_NO1062	Chr12	22021025-22021276	2939	-32	283	3_0	1_0	0	4	48_0	36_0	12_0	NA	0
PH-286	phasi_NO1065	Chr12	22033423-22033674	12147	-324	575	3_2	3_2	4	5	442_35	44_5	398_30	NA	0
PH-287	-	Chr12	22037519-22037770	3845	-74	325	8_4	1_0	4	8	683_46	669_46	14_0	NA	0
PH-288	-	Chr12	22040293-22040544	2523	-32	283	4_2	1_0	2	4	147_4	131_4	16_0	NA	0
PH-289	-	Chr12	22043854-22044105	3310	-95	346	6_4	1_0	4	5	313_28	244_26	69_2	NA	0
PH-290	phasi_NO1069	Chr12	22050656-22050907	6551	-32	283	5_4	No	4	4	255_33	255_33	0_0	LOC_Os12g36030.3	5
PH-291	phasi_NO1070	Chr12	22053986-22054237	3079	-53	304	5_2	No	2	5	130_10	130_10	0_0	LOC_Os12g36030.2	5
PH-292	phasi_NO1071	Chr12	22058131-22058382	3894	-32	283	6_3	2_1	4	8	951_64	756_52	195_12	NA	0
PH-293	phasi_NO1073	Chr12	22062553-22062804	4171	-53	304	8_5	1_1	6	7	707_73	686_66	21_7	NA	0
PH-294	-	Chr12	22066740-22066991	3936	-53	304	5_1	1_0	1	5	283_3	101_3	182_0	NA	0
PH-295	phasi_NO1075	Chr12	22073694-22073945	6703	-32	283	5_3	No	3	3	127_13	127_13	0_0	NA	0
PH-296	-	Chr12	22114897-22115148	40952	-32	283	6_4	No	4	6	500_44	500_44	0_0	NA	0
PH-297	phasi_NO1084	Chr12	22119274-22119525	4126	-93	344	4_1	2_2	3	6	3188_284	61_3	3127_281	NA	0
PH-301	-	Chr12	24252550-24252801	2133025	-51	302	8_5	1_0	5	5	272_63	199_63	73_0	NA	0
PH-302	-	Chr12	24254687-24254938	1886	-284	535	3_3	3_3	6	3	445_109	122_28	323_81	NA	0
PH-303	-	Chr12	24255624-24255875	686	-32	283	4_3	No	3	2	63_7	63_7	0_0	NA	0
PH-304	-	Chr12	24258820-24259071	2945	-32	283	5_5	1_1	6	2	178_40	155_33	23_7	NA	0
PH-305	phasi_NO1089	Chr12	24260273-24260524	1202	-32	283	7_7	No	7	2	185_52	185_52	0_0	NA	0
PH-306	-	Chr12	24261531-24261782	1007	-240	491	3_3	1_1	4	4	154_16	135_14	19_2	RF00005;tRNA;ABSD01000006.1/	1
PH-307	-	Chr12	24278715-24278966	16933	-171	422	3_3	No	3	2	238_75	238_75	0_0	NA	0
PH-308	-	Chr12	24283322-24283573	4356	-281	532	3_3	No	3	2	100_16	100_16	0_0	LOC_Os09g24924	1
PH-309	-	Chr12	24290567-24290818	6994	-74	325	6_5	No	5	4	427_44	427_44	0_0	NA	0
PH-310	-	Chr12	24294549-24294800	3731	-242	493	3_1	No	1	2	44_3	44_3	0_0	NA	0
PH-311	-	Chr12	24295165-24295416	365	-74	325	5_3	No	3	5	251_21	251_21	0_0	NA	0
PH-312	-	Chr12	24301557-24301808	6141	-32	283	5_5	No	5	2	150_30	150_30	0_0	NA	0
PH-313	-	Chr12	24306605-24306856	4797	-95	346	3_2	3_3	5	6	1370_124	112_12	1258_112	NA	0
PH-314	-	Chr12	24310773-24311024	3917	ND	251	4_1	No	1	4	80_3	80_3	0_0	NA	0
PH-315	-	Chr12	24311891-24312142	867	-32	283	3_3	No	3	2	845_17	845_17	0_0	NA	0
PH-316	-	Chr12	24312646-24312897	504	-32	283	7_5	1_1	6	7	696_88	681_83	15_5	NA	0
PH-317	-	Chr12	24313377-24313628	480	-53	304	8_5	1_1	6	7	405_44	344_38	61_6	NA	0
PH-318	phasi_NO1097	Chr12	24315089-24315340	1461	-223	474	10_7	3_2	9	11	856_123	804_115	52_8	LOC_Os12g39450,LOC_Os12g394	9
PH-319	-	Chr12	24317901-24318152	2561	-32	283	3_2	No	2	3	158_10	158_10	0_0	NA	0
PH-320	phasi_NO1098	Chr12	24320131-24320382	1979	-202	453	4_4	No	4	4	1563_175	1563_175	0_0	NA	0
PH-321	-	Chr12	24323452-24323703	3070	-74	325	3_2	No	2	2	98_4	98_4	0_0	LOC_Os12g39470	3
PH-322	phasi_NO1099	Chr12	24324308-24324559	605	-32	283	4_4	No	4	4	149_26	149_26	0_0	LOC_Os12g39470,LOC_Os12g394	4
PH-323	-	Chr12	24327795-24328046	3236	-32	283	3_3	No	3	2	234_18	234_18	0_0	NA	0
PH-324	phasi_NO1100	Chr12	24337794-24338045	9748	-32	283	5_3	No	3	3	229_25	229_25	0_0	Rep_LOC_Os12g39490	5
PH-325	phasi_NO1105	Chr12	24351022-24351273	12977	-32	283	7_6	No	6	6	294_26	294_26	0_0	NA	0
PH-326	phasi_NO1106	Chr12	24357145-24357396	5872	-74	325	5_2	5_3	5	8	408_70	109_10	299_60	NA	0
PH-327	phasi_NO1107	Chr12	24358237-24358488	841	-32	283	9_7	No	7	9	2674_315	2674_315	0_0	osa-MIR5519	2
PH-328	phasi_NO1108	Chr12	24370251-24370502	11763	ND	251	5_3	No	3	5	210_11	210_11	0_0	NA	0
PH-329	phasi_NO1109	Chr12	24371641-24371892	1139	-242	493	3_3	3_2	5	5	523_81	122_21	401_60	NA	0
PH-330	phasi_NO1110	Chr12	24374520-24374771	2628	-53	304	7_6	4_4	10	8	882_147	674_127	208_20	NA	0
PH-331	phasi_NO1112	Chr12	24380669-24380920	5898	-32	283	3_3	No	3	3	1217_122	1217_122	0_0	NA	0
PH-332	phasi_NO1113	Chr12	24385646-24385897	4726	-53	304	3_3	No	3	3	148_24	148_24	0_0	NA	0
PH-333	phasi_NO1115	Chr12	24388051-24388302	2154	-116	367	3_3	No	3	2	109_26	109_26	0_0	NA	0
PH-334	-	Chr12	24388875-24389126	573	-32	283	6_3	No	3	6	369_17	369_17	0_0	NA	0
PH-335	phasi_NO1116	Chr12	24391101-24391352	1975	-173	424	4_0	3_3	3	6	243_53	142_0	101_53	LOC_Os12g39550	7
PH-336	phasi_NO1117	Chr12	24395915-24396166	4563	ND	251	4_1	No	1	4	348_2	348_2	0_0	NA	0
PH-337	phasi_NO1118	Chr12	24398718-24398969	2552	-53	304	6_6	1_0	6	5	435_51	417_51	18_0	LOC_Os12g39560	7
PH-338	phasi_NO1119	Chr12	24401076-24401327	2107	-74	325	2_2	No	2	2	210_26	210_26	0_0	NA	0
PH-339	-	Chr12	24409832-24410083	8505	-32	283	6_6	No	6	5	665_102	665_102	0_0	NA	0
PH-340	-	Chr12	24412467-24412718	2384	ND	251	3_2	No	2	1	43_9	43_9	0_0	NA	0
PH-341	phasi_NO1122	Chr12	24413363-24413614	645	ND	251	5_4	No	4	2	94_20	94_20	0_0	NA	0
PH-342	-	Chr12	24435408-24435659	21794	-53	304	4_4	No	4	3	116_20	116_20	0_0	NA	0
PH-343	phasi_NO1125	Chr12	24828268-24828519	392609	ND	251	8_4	1_1	5	9	453_21	408_18	45_3	LOC_Os12g40150	6
PH-344	phasi_NO1126	Chr12	24828963-24829214	444	ND	251	7_7	2_2	9	2	567_185	528_165	39_20	NA	0
PH-345	phasi_NO1127	Chr12	24831707-24831958	2493	ND	251	11_11	2_2	13	13	1750_352	1230_278	520_74	NA	0
PH-346	phasi_NO1128	Chr12	24833494-24833745	1536	ND	251	3_3	No	3	3	940_112	940_112	0_0	NA	0
PH-347	phasi_NO1130	Chr12	24838646-24838897	4901	ND	251	4_3	1_0	3	5	208_27	197_27	11_0	NA	0
PH-348	phasi_NO1131	Chr12	24840313-24840564	1416	ND	251	6_6	No	6	6	275_53	275_53	0_0	NA	0
PH-349	-	Chr12	24844966-24845217	4402	ND	251	3_3	No	3	2	103_16	103_16	0_0	NA	0
PH-350	-	Chr12	24849343-24849594	4126	ND	251	4_3	No	3	4	181_17	181_17	0_0	NA	0
PH-351	phasi_NO490	Chr12	24850547-24850798	953	ND	251	4_3	No	3	3	533_104	533_104	0_0	NA	0
PH-352	-	Chr12	24851775-24852026	977	ND	251	4_4	No	4	3	298_32	298_32	0_0	NA	0
PH-353	phasi_NO1133	Chr12	24862194-24862445	10168	ND	251	3_2	No	2	3	3316_338	3316_338	0_0	NA	0
PH-354	phasi_NO1134	Chr12	24865805-24866056	3360	ND	251	5_5	No	5	4	734_97	734_97	0_0	NA	0
PH-355	phasi_NO1135	Chr12	24867519-24867770	1463	ND	251	13_12	No	12	10	564_135	564_135	0_0	NA	0
PH-356	phasi_NO1139	Chr12	26988661-26988912	2120891	ND	251	8_7	No	7	5	733_107	733_107	0_0	NA	0

Additional file 6: 21-nt small RNAs associated to phased loci and mature miRNA families detected in young panicles of *O. glaberrima* and *O. barthii*.
(b) Normalized count of reads (relative abundance for 2 millions of total reads) associated with mature miRNA families in relation to small RNA size. #MIR: name of miRNA family; species: *O. barthii* and *O. glaberrima* species lines; 18 to 28: numbers of reads for each size class related to reported mature sequence; Total_per_mir: total number of reads related to reported mature sequences; Class: annotation classes as defined by Jeong et al (2011) based on S-plot patterns, (canonical, variant, siRNA-like).

#MIR	Species	18	19	20	21	22	23	24	25	26	27	28	Total_per_mir	Class
**osa-MIR1318**	*O. barthii*	0	0	0	0	27	0	0	0	0	0	0	27	canonical
	O. glaberrima	0	0	0	0	21	0	0	0	0	0	0	21	
**osa-MIR1320**	*O. barthii*	0	0	0	34	0	0	0	0	0	0	0	34	canonical
	O. glaberrima	0	0	0	48	0	0	0	0	0	0	0	48	
**osa-MIR1423**	*O. barthii*	0	0	0	0	0	0	114	173	0	0	0	287	variant
	O. glaberrima	0	0	0	0	0	11	47	212	0	0	0	271	
**osa-MIR1425**	*O. barthii*	18	47	104	4621	0	0	0	0	0	0	0	4791	canonical
	O. glaberrima	39	92	186	7190	0	0	0	0	0	0	0	7506	
**osa-MIR1426**	*O. barthii*	7	40	0	0	0	0	8	0	0	0	0	55	siRNA-like
	O. glaberrima	6	56	0	0	0	0	3	0	0	0	0	65	
**osa-MIR1428**	*O. barthii*	0	0	0	15	33	0	0	0	0	0	0	48	canonical
	O. glaberrima	0	0	0	12	28	0	0	0	0	0	0	40	
**osa-MIR1429**	*O. barthii*	0	0	0	0	0	0	25	0	0	0	0	25	canonical
	O. glaberrima	0	0	0	0	0	0	31	0	0	0	0	31	
**osa-MIR1430**	*O. barthii*	0	0	0	11	0	0	0	0	0	0	0	11	variant
	O. glaberrima	0	0	0	10	0	0	0	0	0	0	0	10	
**osa-MIR1433**	*O. barthii*	0	0	0	66	0	0	0	0	0	0	0	66	variant
	O. glaberrima	0	0	0	67	0	0	0	0	0	0	0	67	
**osa-MIR1436**	*O. barthii*	0	0	0	0	0	18	8	0	0	0	0	26	siRNA-like
	O. glaberrima	0	0	0	0	0	12	5	0	0	0	0	17	
**osa-MIR1439**	*O. barthii*	0	0	8	0	0	11	439	0	0	0	0	458	siRNA-like
	O. glaberrima	0	0	5	0	0	11	518	0	0	0	0	533	
**osa-MIR1440**	*O. barthii*	0	0	0	0	0	0	23	0	0	0	0	23	siRNA-like
	O. glaberrima	0	0	0	0	0	0	18	0	0	0	0	18	
**osa-MIR1441**	*O. barthii*	0	0	0	5	8	76	86	0	0	0	0	174	siRNA-like
	O. glaberrima	0	0	0	7	4	60	70	0	0	0	0	141	
**osa-MIR1442**	*O. barthii*	0	0	0	0	0	0	3	0	0	0	0	3	siRNA-like
	O. glaberrima	0	0	0	0	0	0	8	0	0	0	0	8	
**osa-MIR156**	*O. barthii*	64	142	2255	4102	0	0	13	0	0	0	0	6577	canonical
	O. glaberrima	73	129	1956	2935	0	0	7	0	0	0	0	5100	
**osa-MIR159**	*O. barthii*	6165	27856	191430	987942	59638	8989	4925	1361	298	191	32	1288827	canonical
	O. glaberrima	7469	31367	200231	1026921	61983	10667	5665	1509	323	186	17	1346338	
**osa-MIR160**	*O. barthii*	51	173	490	16269	34	29	14	18	0	0	0	17079	canonical
	O. glaberrima	54	219	642	17638	62	57	13	13	0	0	0	18699	
**osa-MIR162**	*O. barthii*	12	139	126	5738	151	51	7	0	0	0	0	6225	canonical
	O. glaberrima	18	143	132	5219	129	38	14	0	0	0	0	5693	
**osa-MIR164**	*O. barthii*	49	484	841	18940	63	13	21	24	9	28	0	20471	canonical
	O. glaberrima	45	517	856	17486	47	23	15	30	13	29	0	19060	
**osa-MIR166**	*O. barthii*	420	1860	3322	117220	678	430	163	90	23	31	0	124236	canonical
	O. glaberrima	477	1942	3251	102099	670	490	204	77	28	27	0	109264	
**osa-MIR167**	*O. barthii*	17	41	215	4107	778	0	0	0	0	0	0	5158	canonical
	O. glaberrima	18	52	253	5201	297	0	0	0	0	0	0	5822	
**osa-MIR168**	*O. barthii*	107	1111	475	18675	1397	38	553	9	0	0	0	22365	canonical
	O. glaberrima	125	1160	475	19628	859	28	332	11	0	0	0	22618	
**osa-MIR169**	*O. barthii*	11	152	68	2936	124	5	0	0	0	0	0	3297	canonical
	O. glaberrima	12	94	60	2881	102	7	0	0	0	0	0	3156	
**osa-MIR171**	*O. barthii*	79	172	496	13460	121	14	0	0	0	0	0	14342	canonical
	O. glaberrima	107	147	445	12567	114	23	0	0	0	0	0	13403	
**osa-MIR172**	*O. barthii*	0	0	77	803	0	0	0	0	0	0	0	880	canonical
	O. glaberrima	0	0	119	1208	0	0	0	0	0	0	0	1327	

#MIR	Species	18	19	20	21	22	23	24	25	26	27	28	Total_per_mir	Class
**osa-MIR1846**	*O. barthii*	0	0	16	71	64	184	149	12	0	0	0	497	canonical
	O. glaberrima	0	0	22	43	40	115	46	5	0	0	0	271	
**osa-MIR1849**	*O. barthii*	0	0	0	3	0	0	0	0	0	0	0	3	siRNA-like
	O. glaberrima	0	0	0	8	0	0	0	0	0	0	0	8	
**osa-MIR1850**	*O. barthii*	0	0	0	12	41	6	75	8	0	0	0	142	canonical
	O. glaberrima	0	0	0	21	39	14	102	9	0	0	0	184	
**osa-MIR1852**	*O. barthii*	0	0	0	13	0	0	0	0	0	0	0	13	canonical
	O. glaberrima	0	0	0	6	0	0	0	0	0	0	0	6	
**osa-MIR1859**	*O. barthii*	21	59	397	562	14187	85	32	18	0	0	0	15362	canonical
	O. glaberrima	40	95	529	643	14822	93	35	13	0	0	0	16270	
**osa-MIR1860**	*O. barthii*	0	0	0	30	0	0	0	0	0	0	0	30	variant
	O. glaberrima	0	0	0	7	0	0	0	0	0	0	0	7	
**osa-MIR1861**	*O. barthii*	12	0	54	39	925	0	0	0	0	0	0	1030	canonical
	O. glaberrima	0	0	55	44	1058	0	0	0	0	0	0	1158	
**osa-MIR1862**	*O. barthii*	49	288	1189	1304	1813	4328	115812	725	64	41	30	125644	canonical
	O. glaberrima	83	274	1442	1490	1651	3760	111770	784	57	34	29	121373	
**osa-MIR1863**	*O. barthii*	0	0	0	0	24	36	372	50	0	0	0	482	variant
	O. glaberrima	0	0	0	0	22	58	422	39	0	0	0	540	
**osa-MIR1864**	*O. barthii*	0	0	0	0	0	0	15	0	0	0	0	15	siRNA-like
	O. glaberrima	0	0	0	0	0	0	13	0	0	0	0	13	
**osa-MIR1865**	*O. barthii*	0	0	0	0	0	0	16	0	0	0	0	16	variant
	O. glaberrima	0	0	0	0	0	0	0	0	0	0	0	0	
**osa-MIR1868**	*O. barthii*	0	8	0	0	0	11	35	0	0	0	0	54	siRNA-like
	O. glaberrima	0	4	0	0	0	4	37	0	0	0	0	44	
**osa-MIR1869**	*O. barthii*	0	0	0	0	10	0	30	0	0	0	0	39	siRNA-like
	O. glaberrima	0	0	0	0	15	0	45	0	0	0	0	60	
**osa-MIR1871**	*O. barthii*	0	0	0	0	0	11	160	25	0	0	0	195	variant
	O. glaberrima	0	0	0	0	0	12	162	15	0	0	0	189	
**osa-MIR1872**	*O. barthii*	0	0	0	0	0	6	58	0	0	0	0	64	variant
	O. glaberrima	0	0	0	0	0	7	46	0	0	0	0	53	
**osa-MIR1875**	*O. barthii*	0	0	0	0	0	14	28	0	0	0	0	42	canonical
	O. glaberrima	0	0	0	0	0	15	18	0	0	0	0	33	
**osa-MIR1876**	*O. barthii*	3	0	10	11	16	33	1328	13	0	0	0	1414	canonical
	O. glaberrima	10	0	10	12	24	20	1458	13	0	0	0	1546	
**osa-MIR1877**	*O. barthii*	0	0	0	0	0	0	11	0	0	0	0	11	siRNA-like
	O. glaberrima	0	0	0	0	0	0	6	0	0	0	0	6	
**osa-MIR1878**	*O. barthii*	0	0	0	10	25	214	264	9	0	0	0	521	canonical
	O. glaberrima	0	0	0	6	21	155	177	3	0	0	0	362	
**osa-MIR1880**	*O. barthii*	0	0	0	0	0	6	0	0	0	0	0	6	siRNA-like
	O. glaberrima	0	0	0	10	0	7	0	0	0	0	0	17	
**osa-MIR1883**	*O. barthii*	0	0	0	27	42	169	1327	27	0	0	0	1593	variant
	O. glaberrima	0	0	0	26	38	191	1196	25	0	0	0	1475	
**osa-MIR1884**	*O. barthii*	20	47	136	502	892	2311	3789	486	109	94	13	8398	siRNA-like
	O. glaberrima	32	71	142	460	873	2080	3368	408	78	51	14	7578	
**osa-MIR2055**	*O. barthii*	0	0	35	427	0	0	0	0	0	0	0	463	siRNA-like
	O. glaberrima	0	0	38	471	0	0	0	0	0	0	0	509	
**osa-MIR2098**	*O. barthii*	0	0	0	6	5	0	0	0	0	0	0	11	variant?
	O. glaberrima	0	0	0	11	7	0	0	0	0	0	0	18	
**osa-MIR2106**	*O. barthii*	0	0	0	0	0	0	0	12	0	0	0	12	canonical
	O. glaberrima	0	0	0	0	0	0	0	7	0	0	0	7	
**osa-MIR2118**	*O. barthii*	0	0	0	228	4847	0	0	0	0	0	0	5075	canonical
	O. glaberrima	0	0	0	74	1440	0	0	0	0	0	0	1514	
**osa-MIR2120**	*O. barthii*	0	0	5	0	0	16	93	0	0	0	0	114	siRNA-like
	O. glaberrima	0	0	6	0	0	16	81	0	0	0	0	103	
**osa-MIR2121**	*O. barthii*	0	0	5	9	0	15	1394	0	0	0	0	1423	siRNA-like
	O. glaberrima	0	0	7	6	0	8	1336	0	0	0	0	1357	

#MIR	Species	18	19	20	21	22	23	24	25	26	27	28	Total_per_mir	Class
**osa-MIR2124**	*O. barthii*	0	0	0	0	0	0	71	0	0	0	0	71	siRNA-like
	O. glaberrima	0	0	0	0	0	0	86	0	0	0	0	86	
**osa-MIR2125**	*O. barthii*	25	19	30	28	109	11	462	19	0	0	0	703	siRNA-like
	O. glaberrima	38	14	47	24	89	17	419	11	0	0	0	658	
**osa-MIR2275**	*O. barthii*	0	0	38	138	1430	0	0	0	0	0	0	1606	canonical
	O. glaberrima	0	0	0	4	29	0	0	0	0	0	0	33	
**osa-MIR2862**	*O. barthii*	0	0	0	0	0	0	34	0	0	0	0	34	siRNA-like
	O. glaberrima	0	0	0	0	0	0	17	0	0	0	0	17	
**osa-MIR2863**	*O. barthii*	0	0	0	21	0	0	18	0	0	0	0	39	siRNA-like
	O. glaberrima	0	0	0	11	0	0	15	0	0	0	0	26	
**osa-MIR2867**	*O. barthii*	0	0	0	8	0	0	0	0	0	0	0	8	variant
	O. glaberrima	0	0	0	6	0	0	0	0	0	0	0	6	
**osa-MIR2871**	*O. barthii*	0	11	10	220	5	0	0	0	0	0	0	246	variant
	O. glaberrima	0	8	13	311	8	0	0	0	0	0	0	340	
**osa-MIR2872**	*O. barthii*	0	0	0	0	0	0	26	0	0	0	0	26	siRNA-like
	O. glaberrima	0	0	0	0	0	0	26	0	0	0	0	26	
**osa-MIR2873**	*O. barthii*	0	0	0	147	7	0	135	0	0	0	0	289	siRNA-like
	O. glaberrima	0	0	0	81	12	12	134	0	0	0	0	239	
**osa-MIR2874**	*O. barthii*	0	0	0	0	0	0	74	4	0	0	0	78	canonical
	O. glaberrima	0	0	0	0	0	0	81	9	0	0	0	90	
**osa-MIR2877**	*O. barthii*	0	0	0	6	10	9	44	0	0	0	0	68	canonical
	O. glaberrima	0	0	0	9	7	3	54	0	0	0	0	73	
**osa-MIR2879**	*O. barthii*	0	0	0	0	0	0	58	0	0	0	0	58	canonical
	O. glaberrima	0	0	0	0	0	0	38	0	0	0	0	38	
**osa-MIR2880**	*O. barthii*	0	0	0	5	0	0	451	0	0	0	0	456	siRNA-like
	O. glaberrima	0	0	0	7	0	0	409	0	0	0	0	416	
**osa-MIR2905**	*O. barthii*	0	0	0	0	0	0	491	0	0	0	0	491	siRNA-like
	O. glaberrima	0	0	0	0	0	0	527	0	0	0	0	527	
**osa-MIR390**	*O. barthii*	11	13	42	1240	0	0	35	0	0	0	0	1342	canonical
	O. glaberrima	8	10	53	1152	0	0	23	0	0	0	0	1246	
**osa-MIR393**	*O. barthii*	15	6	65	257	0	0	0	0	0	0	0	343	canonical
	O. glaberrima	24	6	103	410	0	0	0	0	0	0	0	542	
**osa-MIR394**	*O. barthii*	19	149	5661	349	44	65	39	12	0	0	0	6339	canonical
	O. glaberrima	40	227	8629	496	69	116	91	14	0	0	0	9682	
**osa-MIR395**	*O. barthii*	0	0	192	0	0	0	0	0	0	0	0	192	canonical
	O. glaberrima	0	0	209	10	0	0	0	0	0	0	0	219	
**osa-MIR396**	*O. barthii*	11	34	132	4627	36	0	0	0	0	0	0	4840	variant
	O. glaberrima	4	25	109	3761	26	0	0	0	0	0	0	3924	
**osa-MIR397**	*O. barthii*	0	0	11	255	76	0	0	0	0	0	0	341	canonical
	O. glaberrima	0	0	11	182	57	0	0	0	0	0	0	250	
**osa-MIR3979**	*O. barthii*	0	0	92	24	0	0	0	0	0	0	0	116	canonical
	O. glaberrima	0	0	144	23	0	0	0	0	0	0	0	167	
**osa-MIR3981**	*O. barthii*	0	0	0	0	0	0	6	0	0	0	0	6	siRNA-like
	O. glaberrima	0	0	0	0	0	0	5	0	0	0	0	5	
**osa-MIR399**	*O. barthii*	0	0	10	98	0	0	0	0	0	0	0	107	canonical
	O. glaberrima	0	0	12	109	0	0	0	0	0	0	0	121	
**osa-MIR408**	*O. barthii*	0	0	10	55	0	0	0	0	0	0	0	64	canonical
	O. glaberrima	0	0	11	73	0	0	0	0	0	0	0	84	
**osa-MIR419**	*O. barthii*	0	0	0	0	0	0	11	0	0	0	0	11	canonical
	O. glaberrima	0	0	0	0	0	0	7	0	0	0	0	7	
**osa-MIR435**	*O. barthii*	0	0	0	11	55	0	12	0	0	0	0	78	canonical
	O. glaberrima	0	0	0	14	77	0	3	0	0	0	0	94	
**osa-MIR437**	*O. barthii*	0	0	0	3	0	0	370	0	0	0	0	373	variant
	O. glaberrima	0	0	0	9	0	0	353	0	0	0	0	362	
**osa-MIR439**	*O. barthii*	0	0	0	0	0	0	13	0	0	0	0	13	siRNA-like
	O. glaberrima	0	0	0	0	0	0	10	0	0	0	0	10	

#MIR	Species	18	19	20	21	22	23	24	25	26	27	28	Total_per_mir	Class
**osa-MIR441**	*O. barthii*	0	0	0	5	0	34	1195	0	0	0	0	1234	siRNA-like
	O. glaberrima	0	0	0	7	0	35	983	0	0	0	0	1024	
**osa-MIR442**	*O. barthii*	0	0	0	14	0	41	54	0	0	0	0	109	siRNA-like
	O. glaberrima	0	0	0	11	0	29	54	0	0	0	0	94	
**osa-MIR443**	*O. barthii*	0	0	0	0	0	9	25	0	0	0	0	34	siRNA-like
	O. glaberrima	0	0	0	0	0	15	35	0	0	0	0	49	
**osa-MIR444**	*O. barthii*	9	36	57	2483	14	0	0	0	0	0	0	2600	canonical
	O. glaberrima	20	64	96	3575	26	0	0	0	0	0	0	3780	
**osa-MIR445**	*O. barthii*	0	0	0	0	0	5	146	0	0	0	0	150	siRNA-like
	O. glaberrima	0	0	0	0	0	8	141	0	0	0	0	149	
**osa-MIR5072**	*O. barthii*	14	441	290	490	140	172	0	0	0	0	0	1548	siRNA-like
	O. glaberrima	17	623	507	947	312	293	0	0	0	0	0	2699	
**osa-MIR5073**	*O. barthii*	0	0	0	0	0	0	0	0	13	0	0	13	siRNA-like
	O. glaberrima	0	0	0	0	0	0	0	0	8	0	0	8	
**osa-MIR5076**	*O. barthii*	0	27	0	0	0	0	0	0	0	0	0	27	canonical
	O. glaberrima	0	0	0	0	0	0	0	0	0	0	0	0	
**osa-MIR5083**	*O. barthii*	10	200	65	122	336	617	72	5	0	0	0	1426	canonical
	O. glaberrima	27	256	92	141	292	846	84	7	0	0	0	1744	
**osa-MIR5143**	*O. barthii*	0	0	0	0	0	0	7	0	0	0	0	7	variant
	O. glaberrima	0	0	0	0	0	0	5	0	0	0	0	5	
**osa-MIR5145**	*O. barthii*	0	0	0	46	0	15	617	10	0	0	0	688	siRNA-like
	O. glaberrima	0	0	0	9	0	7	386	7	0	0	0	409	
**osa-MIR5146**	*O. barthii*	0	0	0	0	0	0	11	0	0	0	0	11	canonical
	O. glaberrima	0	0	0	0	0	0	0	0	0	0	0	0	
**osa-MIR5148**	*O. barthii*	0	0	0	0	39	76	249	6	0	0	0	370	siRNA-like
	O. glaberrima	0	0	0	0	34	73	297	5	0	0	0	409	
**osa-MIR5149**	*O. barthii*	0	0	0	0	0	5	377	0	0	0	0	382	siRNA-like
	O. glaberrima	0	0	0	0	0	8	294	0	0	0	0	302	
**osa-MIR5150**	*O. barthii*	0	0	0	0	0	0	251	4	0	0	0	255	siRNA-like
	O. glaberrima	0	0	0	0	0	0	200	8	0	0	0	207	
**osa-MIR5151**	*O. barthii*	0	0	0	0	11	0	0	0	0	0	0	11	siRNA-like
	O. glaberrima	0	0	0	0	2	0	0	0	0	0	0	2	
**osa-MIR5153**	*O. barthii*	0	0	0	0	9	0	210	11	0	0	0	230	siRNA-like
	O. glaberrima	0	0	0	0	4	0	232	8	0	0	0	244	
**osa-MIR5154**	*O. barthii*	0	0	0	0	0	0	35	0	0	0	0	35	siRNA-like
	O. glaberrima	0	0	0	0	0	0	46	0	0	0	0	46	
**osa-MIR5155**	*O. barthii*	0	0	0	0	0	0	18	0	0	0	0	18	canonical
	O. glaberrima	0	0	0	0	0	0	17	0	0	0	0	17	
**osa-MIR5159**	*O. barthii*	0	0	0	2	0	6	126	0	0	0	0	134	canonical
	O. glaberrima	0	0	0	10	0	12	181	0	0	0	0	202	
**osa-MIR5160**	*O. barthii*	0	0	0	0	6	0	21	0	0	0	0	27	siRNA-like
	O. glaberrima	0	0	0	0	5	0	22	0	0	0	0	27	
**osa-MIR5161**	*O. barthii*	0	0	10	0	0	0	0	0	0	0	0	10	siRNA-like
	O. glaberrima	0	0	11	0	0	0	0	0	0	0	0	11	
**osa-MIR528**	*O. barthii*	30	344	99	5540	0	0	0	0	0	0	0	6012	canonical
	O. glaberrima	17	206	59	3211	0	0	0	0	0	0	0	3494	
**osa-MIR529**	*O. barthii*	0	12	42	1688	13	0	0	0	0	0	0	1756	canonical
	O. glaberrima	0	8	46	1561	14	0	0	0	0	0	0	1629	
**osa-MIR530**	*O. barthii*	0	0	0	10	0	0	0	0	0	0	0	10	canonical
	O. glaberrima	0	0	0	11	0	0	0	0	0	0	0	11	
**osa-MIR5337**	*O. barthii*	0	0	0	0	0	0	21	0	0	0	0	21	siRNA-like
	O. glaberrima	0	0	0	0	0	0	23	0	0	0	0	23	
**osa-MIR5340**	*O. barthii*	0	0	0	0	0	0	39	0	0	0	0	39	siRNA-like
	O. glaberrima	0	0	0	0	0	0	64	0	0	0	0	64	
**osa-MIR535**	*O. barthii*	466	1051	2484	122449	83	52	102	74	16	0	0	126777	canonical
	O. glaberrima	417	968	2614	110440	66	38	75	60	21	0	0	114698	

#MIR	Species	18	19	20	21	22	23	24	25	26	27	28	Total_per_mir	Class
**osa-MIR5486**	*O. barthii*	0	0	0	9	0	0	0	0	0	0	0	9	siRNA-like
	O. glaberrima	0	0	0	5	0	0	0	0	0	0	0	5	
**osa-MIR5488**	*O. barthii*	0	0	0	18	0	0	0	0	0	0	0	18	canonical
	O. glaberrima	0	0	0	0	0	0	0	0	0	0	0	0	
**osa-MIR5490**	*O. barthii*	0	0	0	0	0	0	8	0	0	0	0	8	siRNA-like
	O. glaberrima	0	0	0	0	0	0	21	0	0	0	0	21	
**osa-MIR5495**	*O. barthii*	0	0	8	348	0	0	0	0	0	0	0	355	canonical
	O. glaberrima	0	0	3	99	0	0	0	0	0	0	0	102	
**osa-MIR5497**	*O. barthii*	0	21	0	424	0	0	0	0	0	0	0	445	canonical
	O. glaberrima	0	0	0	41	0	0	0	0	0	0	0	41	
**osa-MIR5498**	*O. barthii*	0	0	0	0	0	0	12	0	0	0	0	12	canonical
	O. glaberrima	0	0	0	0	0	0	17	0	0	0	0	17	
**osa-MIR5500**	*O. barthii*	0	0	0	0	0	0	7	0	0	0	0	7	canonical
	O. glaberrima	0	0	0	0	0	0	6	0	0	0	0	6	
**osa-MIR5502**	*O. barthii*	0	0	0	0	0	0	5	0	0	0	0	5	canonical
	O. glaberrima	0	0	0	0	0	0	13	0	0	0	0	13	
**osa-MIR5505**	*O. barthii*	0	11	11	0	0	0	0	0	0	0	0	22	variant
	O. glaberrima	0	7	7	0	0	0	0	0	0	0	0	14	
**osa-MIR5508**	*O. barthii*	0	0	0	11	0	0	22	0	0	0	0	33	canonical
	O. glaberrima	0	0	0	15	0	0	14	0	0	0	0	29	
**osa-MIR5514**	*O. barthii*	0	0	0	27	0	0	0	0	0	0	0	27	variant
	O. glaberrima	0	0	0	2	0	0	0	0	0	0	0	2	
**osa-MIR5516**	*O. barthii*	0	0	0	151	0	0	0	0	0	0	0	151	canonical
	O. glaberrima	0	0	0	6	0	0	0	0	0	0	0	6	
**osa-MIR5517**	*O. barthii*	0	0	0	32	0	0	0	0	0	0	0	32	canonical
	O. glaberrima	0	0	0	3	0	0	0	0	0	0	0	3	
**osa-MIR5519**	*O. barthii*	0	0	11	1097	0	0	0	0	0	0	0	1109	canonical
	O. glaberrima	0	0	2	133	0	0	0	0	0	0	0	135	
**osa-MIR5522**	*O. barthii*	0	0	0	0	0	0	0	26	0	0	0	26	siRNA-like
	O. glaberrima	0	0	0	0	0	0	0	19	0	0	0	19	
**osa-MIR5526**	*O. barthii*	0	0	0	0	11	134	1435	19	0	0	0	1599	siRNA-like
	O. glaberrima	0	0	0	0	9	101	1243	12	0	0	0	1364	
**osa-MIR5529**	*O. barthii*	0	0	0	34	0	0	0	0	0	0	0	34	variant
	O. glaberrima	0	0	0	6	0	0	0	0	0	0	0	6	
**osa-MIR5532**	*O. barthii*	0	0	0	0	0	0	8	0	0	0	0	8	siRNA-like
	O. glaberrima	0	0	0	0	0	0	17	0	0	0	0	17	
**osa-MIR5533**	*O. barthii*	0	0	0	0	0	0	7	0	0	0	0	7	siRNA-like
	O. glaberrima	0	0	0	0	0	0	9	0	0	0	0	9	
**osa-MIR5534**	*O. barthii*	0	0	0	0	0	0	12	0	0	0	0	12	siRNA-like
	O. glaberrima	0	0	0	0	0	0	0	0	0	0	0	0	
**osa-MIR5536**	*O. barthii*	0	0	0	0	18	28	408	0	0	0	0	454	siRNA-like
	O. glaberrima	0	0	0	0	21	24	328	0	0	0	0	372	
**osa-MIR5537**	*O. barthii*	0	0	0	0	0	0	4	0	0	0	0	4	siRNA-like
	O. glaberrima	0	0	0	0	0	0	10	0	0	0	0	10	
**osa-MIR5540**	*O. barthii*	0	0	0	0	0	0	35	0	0	0	0	35	siRNA-like
	O. glaberrima	0	0	0	0	0	0	32	0	0	0	0	32	
**osa-MIR806**	*O. barthii*	0	0	24	59	130	188	4813	43	0	0	0	5257	siRNA-like
	O. glaberrima	0	0	36	58	129	179	4924	78	0	0	0	5404	
**osa-MIR807**	*O. barthii*	0	0	0	14	0	5	75	0	0	0	0	94	siRNA-like
	O. glaberrima	0	0	0	4	0	6	70	0	0	0	0	80	
**osa-MIR808**	*O. barthii*	12	27	168	201	283	1469	34416	396	0	21	0	36994	siRNA-like
	O. glaberrima	23	32	182	184	236	1300	30364	330	0	26	0	32675	
**osa-MIR810**	*O. barthii*	0	0	0	11	0	0	0	0	0	0	0	11	variant
	O. glaberrima	0	0	0	23	0	0	0	0	0	0	0	23	
**osa-MIR811**	*O. barthii*	0	0	8	11	11	67	1145	29	0	0	0	1271	siRNA-like
	O. glaberrima	0	0	3	14	9	37	939	25	0	0	0	1026	

#MIR	Species	18	19	20	21	22	23	24	25	26	27	28	Total_per_mir	Class
**osa-MIR812**	*O. barthii*	0	24	67	88	387	1232	41480	305	0	0	0	43584	siRNA-like
	O. glaberrima	0	21	80	99	395	1163	35874	230	0	0	0	37862	
**osa-MIR813**	*O. barthii*	0	0	0	0	0	0	40	0	0	0	0	40	siRNA-like
	O. glaberrima	0	0	0	0	0	0	56	0	0	0	0	56	
**osa-MIR814**	*O. barthii*	0	0	8	8	41	59	1523	0	0	0	0	1638	siRNA-like
	O. glaberrima	0	0	6	9	30	33	1162	0	0	0	0	1239	
**osa-MIR815**	*O. barthii*	0	0	0	0	60	861	5434	229	0	0	0	6585	siRNA-like
	O. glaberrima	0	0	0	0	50	464	4160	198	0	0	0	4872	
**osa-MIR816**	*O. barthii*	0	0	0	0	0	0	25	0	0	0	0	25	siRNA-like
	O. glaberrima	0	0	0	0	0	0	28	0	0	0	0	28	
**osa-MIR817**	*O. barthii*	0	0	0	0	0	0	129	0	0	0	0	129	siRNA-like
	O. glaberrima	0	0	0	0	0	0	115	0	0	0	0	115	
**osa-MIR818**	*O. barthii*	0	0	0	0	0	0	26	0	0	0	0	26	siRNA-like
	O. glaberrima	0	0	0	0	0	0	15	0	0	0	0	15	
**osa-MIR820**	*O. barthii*	13	388	373	1973	2848	1093	52220	320	40	36	127	59431	canonical
	O. glaberrima	3	391	335	1956	2550	853	43166	238	33	53	172	49750	
**osa-MIR827**	*O. barthii*	0	0	0	79	0	0	0	0	0	0	0	79	siRNA-like
	O. glaberrima	0	0	0	49	0	0	0	0	0	0	0	49	
**Total per size**	*O. barthii*	7741	35435	211769	1343133	92156	23372	280606	4572	573	441	202	2000000	
	O. glaberrima	9244	39228	224281	1353772	88921	23646	255283	4429	560	405	231	2000000	
