# Supplementary material for: miR2118-triggered phased siRNAs are differentially expressed during the panicle development of wild and domesticated African rice species
Source: Rice (N Y). 2016 Mar 12;9:10. doi: 10.1186/s12284-016-0082-9 (PMC4788661; doi:10.1186/s12284-016-0082-9)

Additional file 7. Genomic distribution, abundance and complexity of *O. barthii* and *O. glaberrima* 21-nt phased small RNAs on *O. sativa nipponbare* genome (MSU 7.0).

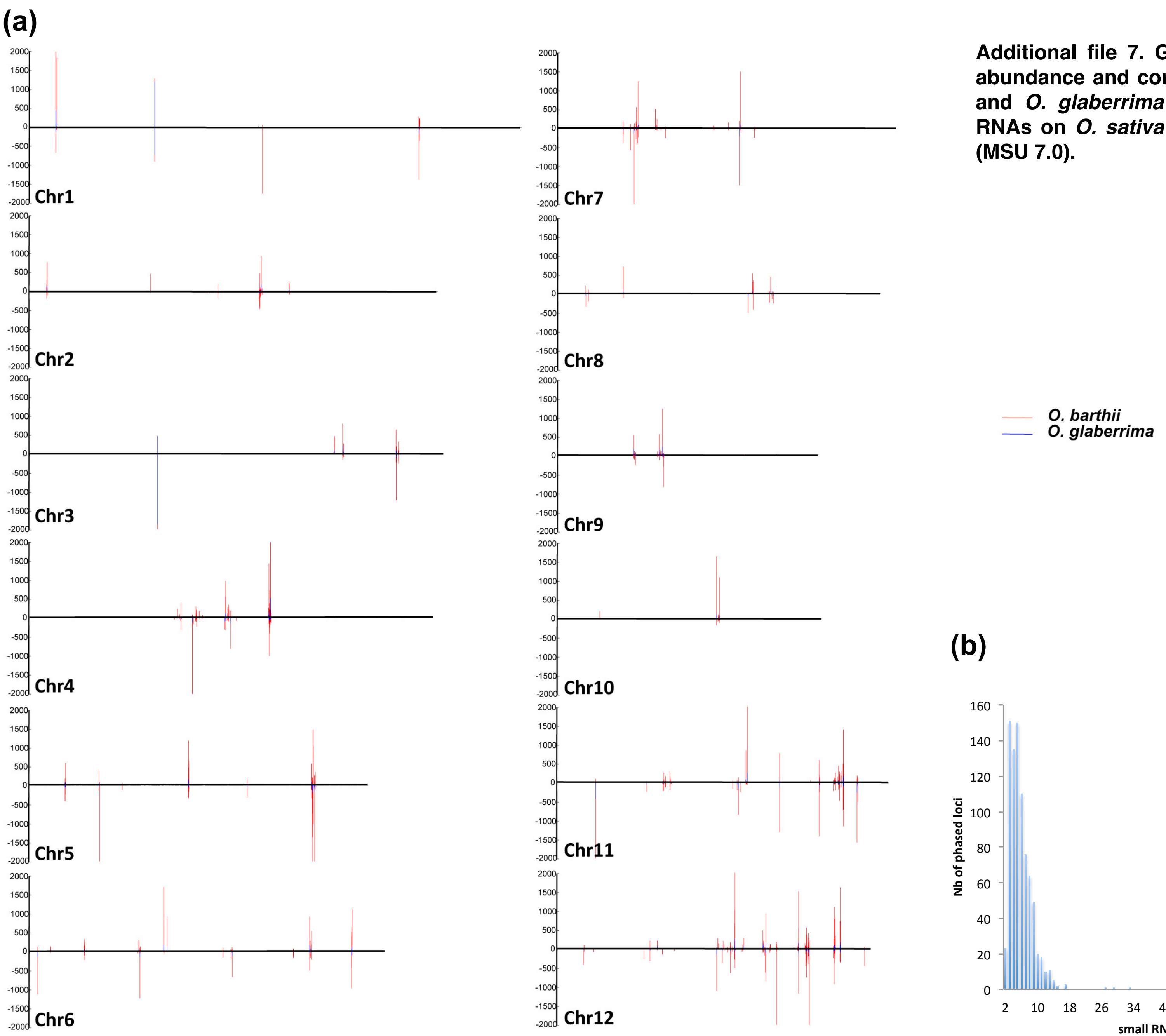

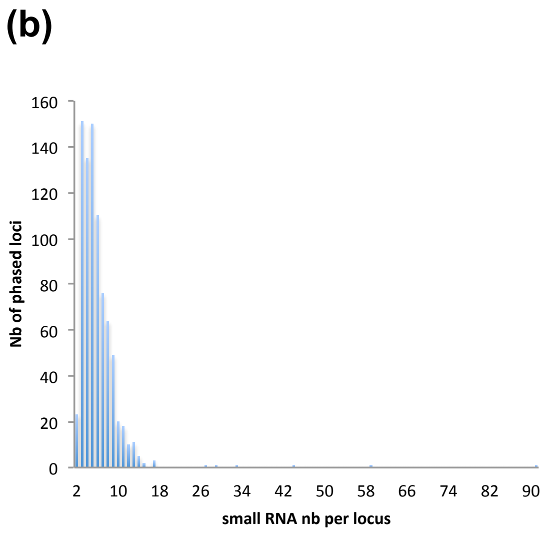

Supplement: Additional file 7: — Genomic distribution, abundance and complexity of O. barthii and O. glaberrima 21-nt phased small RNAs on O. sativa nipponbare genome (MSU 7.0). (a) Genomic distribution and abundance of O. barthii and O. glaberrima 21-nt phasiRNAs on O. sativa nipponbare genome (MSU 7.0). (b) Distribution of number of detected 21-nt phasiRNAs from African species per phased locus on O. sativa nipponbare genome (MSU 7.0). (PDF 547 kb) [file 12284_2016_82_MOESM7_ESM.pdf]
