# Supplementary material for: miR2118-triggered phased siRNAs are differentially expressed during the panicle development of wild and domesticated African rice species
Source: Rice (N Y). 2016 Mar 12;9:10. doi: 10.1186/s12284-016-0082-9 (PMC4788661; doi:10.1186/s12284-016-0082-9)

Additional file 8. Sequences and phasiRNA abundances of the 4 phased loci used for validation.

>phased_12 Chr1-2244143..2244489

5'AAUAGGCUUUGGAGACACCGGGAAUGGAAAACAUAGUUAGAUAUCAAUCUCUCGAUCGUUGAUGCCAUAAGCUACUUGGCUUG
    ||||..|.||||.||.|.||||
 3' AUCCUUACCCUCCGUAGUCCUU 5' ***miR2118f***

GUUUCCAUCCUGCGAGCUGUUAACCAGAAAGGGGAUGGUUGACAGUAGCCUGGAGUUUGGAUCGUUAUUGUGGAACUUGAAUU

AUGAAUCUGUCAUACAUUCCAAAAUUUACGUUGAGAAAUUGAGCUGAAAUCUGCUCGUUGAAGGAACCAACGACUCCUGCCAC

GUUCAAUCUUUUUGCCAGAGAAUGAAGAGUAUGCAUGGAGGUGAAUUCUGAGCUUAUCGGAUCAGUUACUCAAUUGUUUGAAG

AUUAUACCUUGAAGA 3'

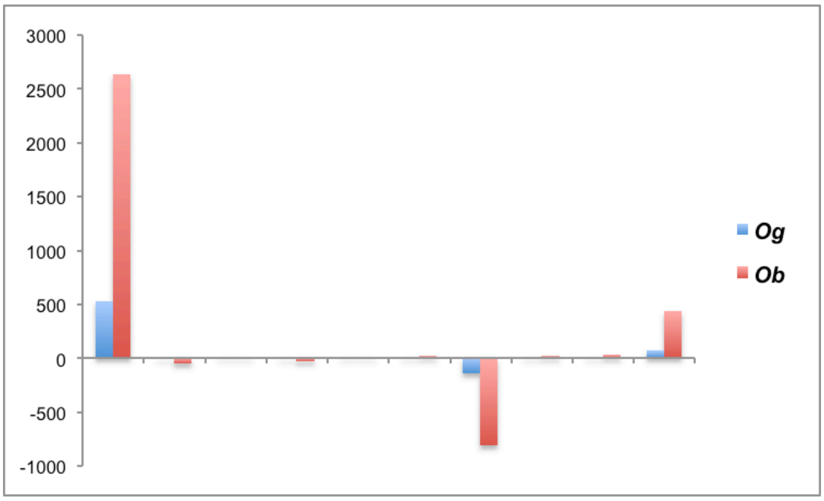

>phased_557 Chr4-21018743-21018938

```
5'AUAGGGCAUGGGAUGCGUC-GGAAAUUCAGGCAGCUCGUUCUGAUGAUUUGCGGCAUAGGAAACGUUGGGGAGCAGCGGGUAC
     ..||.|.||||.||.||.||||
 3' AUCCUUACCCUCCGUAGUCCUU 5' miR2118f
```

UCUGCUGUUUGUUAAUUCCUCGACCGUCGACUGGUGGUUUGUCGGUGUGACCAGAUGUGUCCUGUUCUAUCCUUUGGUACGCG

ACAAAUUUCUGCAACCAUAAAACCCCAA 3′

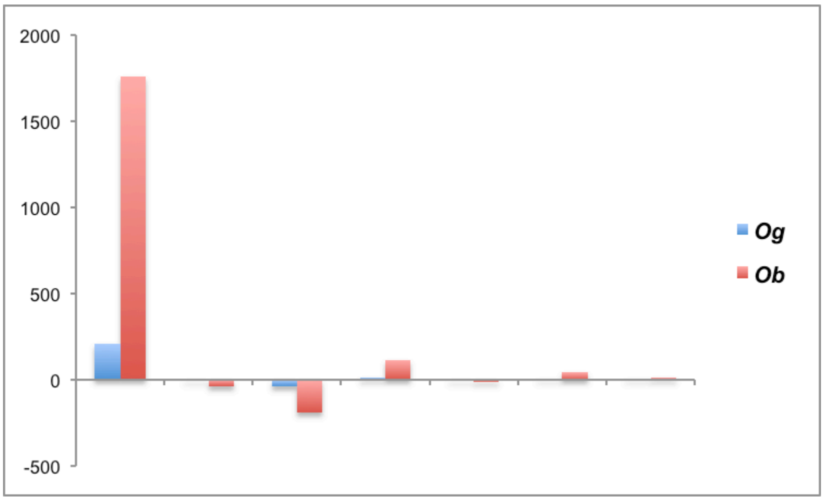

>phased_612 Chr5-24872805..24873056

```
5′
GUUGCGAAUGUGAUGCAUUGGGAAUACGAGUGGAACGAACCAGCCAUCAGAAAGCUCAUGCCAACCAGCGAGGUGUACUAAUAAG
      |..|||||.||.||||..||||
   3′ AUCCUUACCCUCCGUAGUCCUU 5' miR2118f
```

UAGCUACUAUGAGCGACGCUCUUCAGGGAUGCCAACCAACGUGUAGGUUUCUCGUCAAUCCAAUCCUUGGUCACCGUUCCCCA

AGUCCUGCUGGACACUUUUGGUAAAGAAAAAGAUAAUGAAAUUCUUCUGAUCCCGUUAUGUCUCUUCUGCAAAUGUUAAAUUU

UUCAUUAUAAGCUGCAAUUCAGAUUCUUUGUAUAG 3′

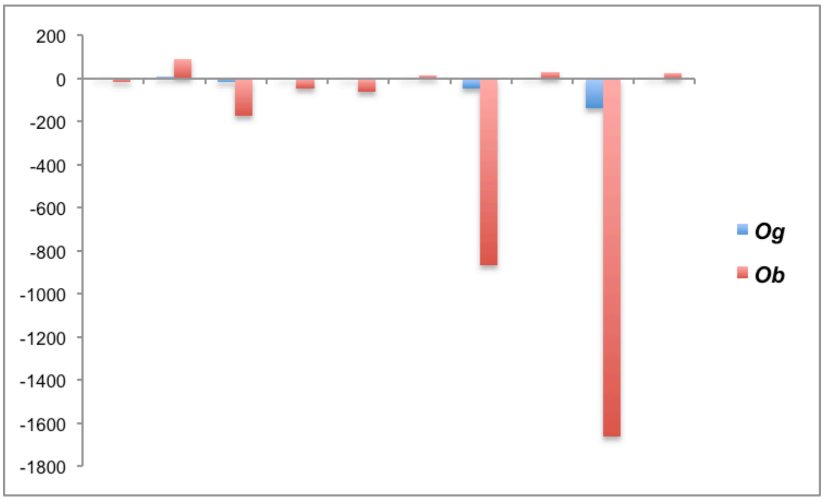

>phased_779 Chr7-16046295..16046641

```
5′
UUUGGGUUUGGGAGGCUUGGGGAAGUGAGGUGCUACUAUCUUGCAUUUCUUGCUUAGCUGCUGCGAAGAUUUCAGCUGCAUCAUU
     |.||..||||||||.|..||||
  3' AUCCUUACCCUCCGUAGUCCUU 5' miR2118f
```

GCUCAGCUGGUGUGAAGAUUCCUGCUCACUCGCUUUGUCCACAAUAGGGCAUCACUAUCGGCAAUGACAAGAGUAGUGGAAAA

AAUAGAAGCAGUACAUGAAAGCUUCUACAGUGACCGUAUCGAACGCCAUAGAUGAUCAUGAAUCAGCAGUAGAUGAUAUCAGA

UGAUCUAUCUCCUGGCUUUUAGUUGUAUGAACGCUGGAUUUGAGGUGAAUUACUUGUUUGUGUUGCUGAUCGAGUUUUGAGCG

UGCGCCUAACUAGUU 3′

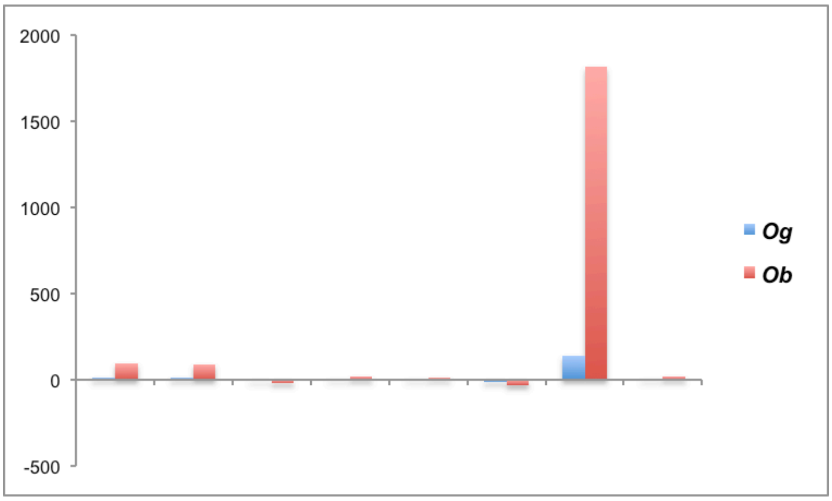

Supplement: Additional file 8: — Sequences and phasiRNA abundances of the 4 phased loci used for validation. The 4 phased loci reported here were used for stem-loop RT-PCR (phasiRNAs) and classical RT-PCR (lncRNAs) validation. The positions of the phased loci on MSU7.0 chromosomes are indicated. The alignment between miR2118f and the phased locus is indicated. The miR2118 recognition site is in blue. The phasiRNAs detected in the plus strand are highlighted alternately in green and in red. The phasiRNAs detected in the minus strand are underlined alternately in green and in red. The phasiRNA boxed in yellow is the most abundant one and the one used for stem-loop RT-PCR validation and qRT-PCR analysis. Histograms illustrate the relative abundance of the detected phasiRNAs per locus in the two species (PDF 159 kb) [file 12284_2016_82_MOESM8_ESM.pdf]
