# Supplementary material for: miR2118-triggered phased siRNAs are differentially expressed during the panicle development of wild and domesticated African rice species
Source: Rice (N Y). 2016 Mar 12;9:10. doi: 10.1186/s12284-016-0082-9 (PMC4788661; doi:10.1186/s12284-016-0082-9)
Supplement: Additional file 9: — Features of O. barthii and O. glaberrima panicle-derived microRNAs and abundance of sequences related to microRNA precursor sequences. (a) Size distribution of clusters (distinct) and reads (abundance) related to O. sativa microRNAs in O. barthii and O. glaberrima. (b) Relative abundance of O. barthii and O. glaberrima reads related to O. sativa microRNA precursors. The precursors are classified according to the abundance distribution pattern as defined by Jeong et al. (2011) (i.e. canonical, variant, siRNA-like). (PDF 200 kb) [file 12284_2016_82_MOESM9_ESM.pdf]

Additional file 9. Features of *O. barthii* and *O. glaberrima* panicle-derived microRNAs and abundance of sequences related to microRNA precursor sequences.

(a)

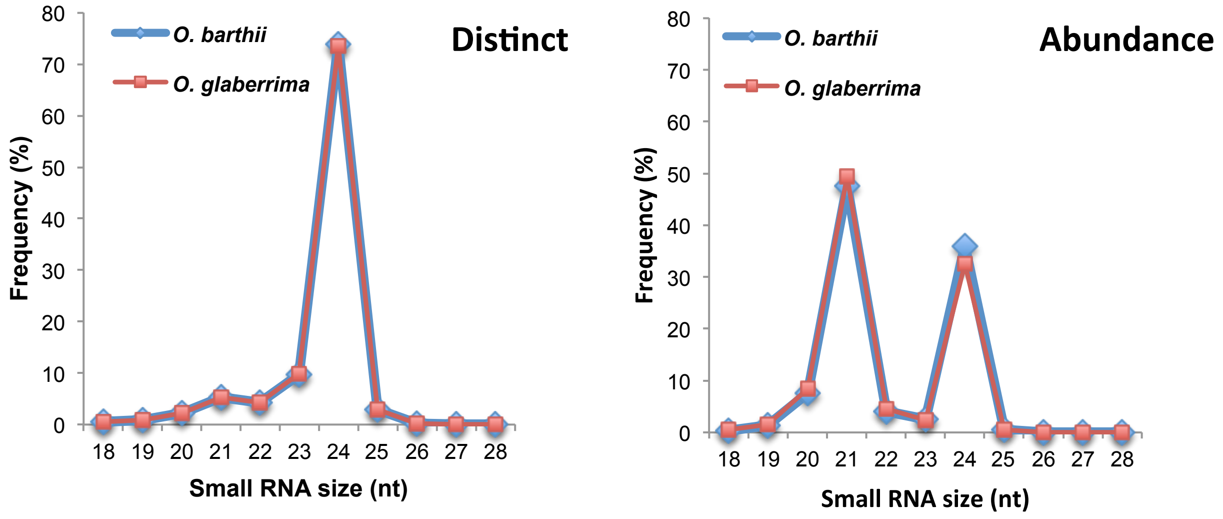

(b)

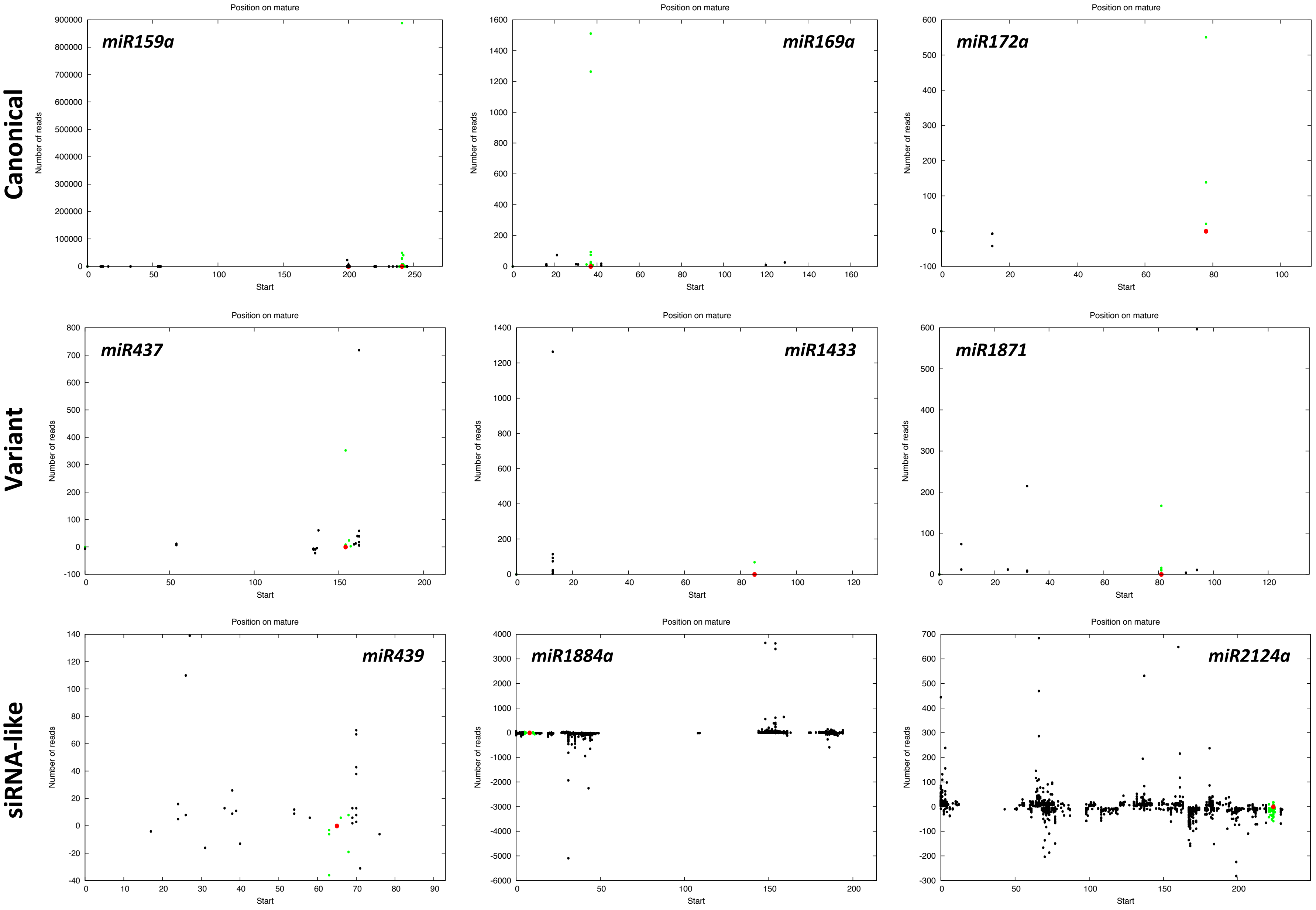
Canonical
Variant
siRNA-like
Position on mature
Number of reads
Start
miR159a
miR169a
miR172a
miR437
miR1433
miR1871
miR439
miR1884a
miR2124a
