# Supplementary material for: miR2118-triggered phased siRNAs are differentially expressed during the panicle development of wild and domesticated African rice species
Source: Rice (N Y). 2016 Mar 12;9:10. doi: 10.1186/s12284-016-0082-9 (PMC4788661; doi:10.1186/s12284-016-0082-9)

Additional file 10. Histological description of selected developmental stages of African rice panicles.

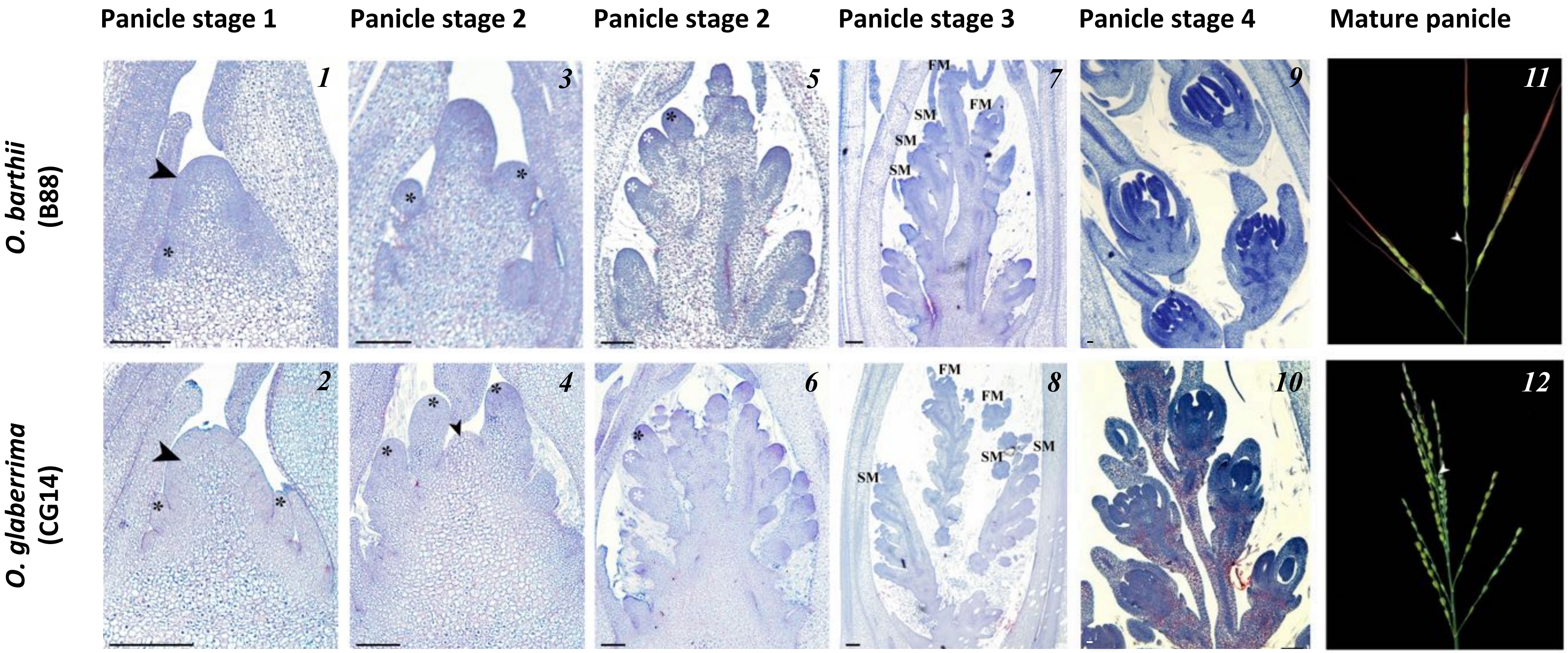

Supplement: Additional file 10: — Histological description of selected developmental stages of African rice panicles. O. barthii: 1,3,5,7,9,11; O. glaberrima: 2,4,6,8,10,12; stage 1: unbranched stage with elongation of rachis meristem (arrowheads) and formation of primary branch meristems (*) (1,2); stage 2: early branching stage with rachis meristem (arrowheads) and elongating primary branches (3,4). At the end of this stage, secondary branches (white *) are initiated from PBs (*) (5,6); stage 3: late branching stage with elongated secondary branch and spikelet meristem (SM) and floret meristem (FM) differentiation (7,8); stage 4: floret organ differentiation/development (9,10); mature stage: 11 and 12. White arrowhead: vestige of aborted rachis meristem. Scale bar: 100 μm. (PDF 1544 kb) [file 12284_2016_82_MOESM10_ESM.pdf]
