# Supplementary material for: miR2118-triggered phased siRNAs are differentially expressed during the panicle development of wild and domesticated African rice species
Source: Rice (N Y). 2016 Mar 12;9:10. doi: 10.1186/s12284-016-0082-9 (PMC4788661; doi:10.1186/s12284-016-0082-9)
Supplement: Additional file 11: — RNA-seq data validation by northern-blotting and semi-quantitative RT-PCR for miRNAs, phasiRNAs and ncRNAs associated with phased loci in panicle-derived small RNA bulks from O. barthii (Ob) and O. glaberrima (Og). (a) Northern blot hybridization using miR2118 as probe. U6 probe was used as control. (b) Stem-loop RT- PCR analysis of various miRNAs and phasiRNAs. miR159a probe was used as a loading control. (c) Classic RT-PCR analysis of ncRNAs associated with 21-nt phased small RNA loci using from polydT primer (dT) or random hexamer primers (RH) for the RTs. The ACTIN gene (Os03g50885) was used as a loading control. (d) Test of specificity of stem-loop RT-PCRs against phasiRNAs on PH12 and PH779 loci, in conjunction with phasiRNA specific forward primer (PH12-F and PH779-F primers; phasiRNA label) and long ncRNA forward primer (PH12-F2 and PH779-F2 primers; long ncRNA label). Controls: 1. RT reaction using O. glaberrima RNA bulk without stem-loop RT primer; 2. RT reaction using O. barthii RNA bulk without stem-loop RT primer; 3. RT-PCR without stem-loop RT matrix; Ø: RT-PCR without RT. See Additional file 1: Table S5 for primer and probe sequences. (PDF 1888 kb) [file 12284_2016_82_MOESM11_ESM.pdf]

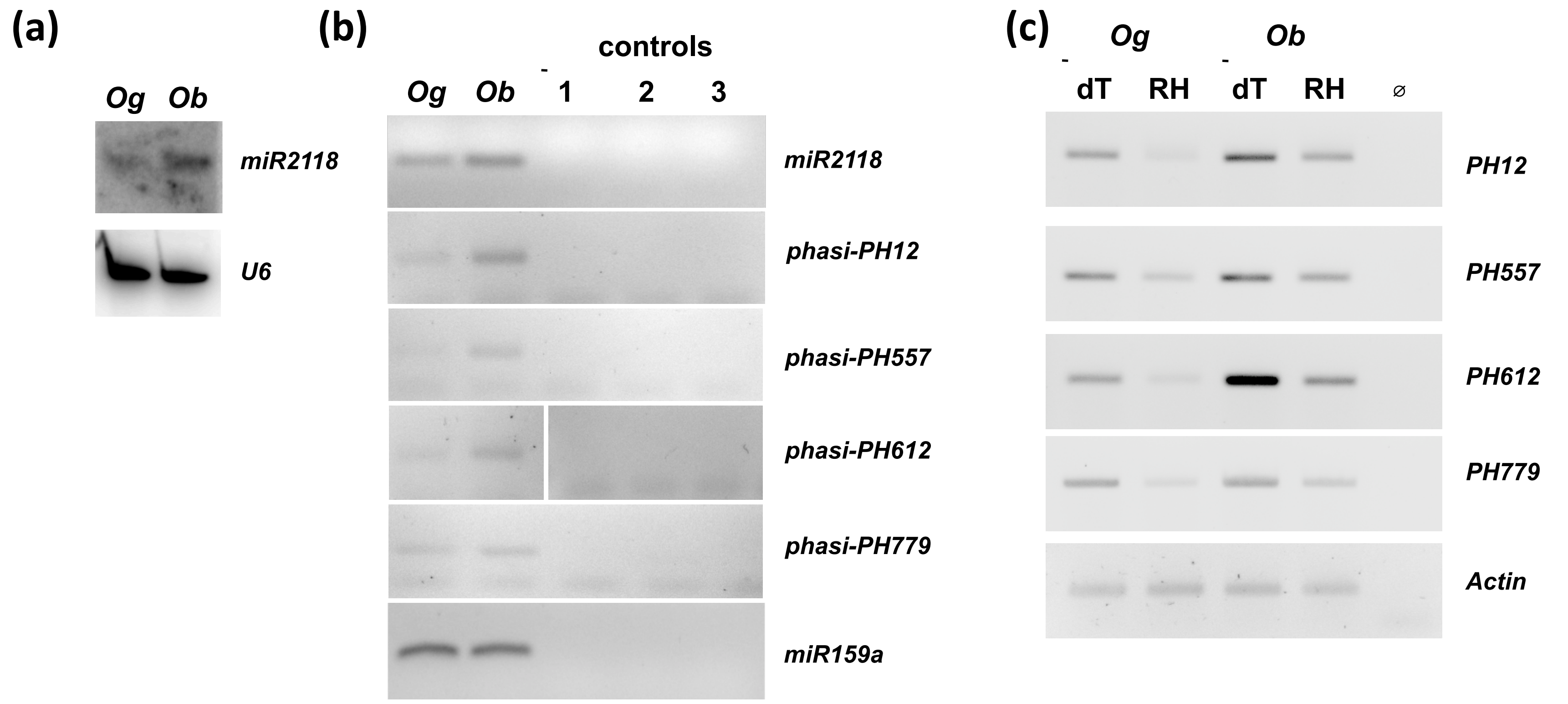

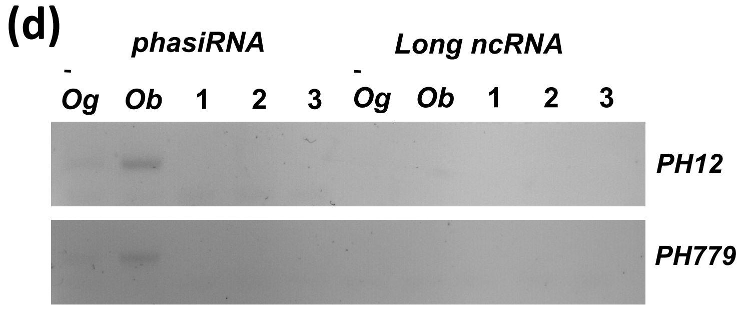

Additional file 11. RNA-seq data validation by northern-blotting and semi-quantitative RT-PCR for miRNAs, phasiRNAs and ncRNAs associated with phased loci in panicle-derived small RNA bulks from *O. barthii* (*Ob*) and *O. glaberrima* (*Og*).
