# Supplementary material for: miR2118-triggered phased siRNAs are differentially expressed during the panicle development of wild and domesticated African rice species
Source: Rice (N Y). 2016 Mar 12;9:10. doi: 10.1186/s12284-016-0082-9 (PMC4788661; doi:10.1186/s12284-016-0082-9)
Supplement: Additional file 12: — Expression patterns of phasiRNAs and lncRNA precursors associated with 21-nucleotide phased loci in O. glaberrima and O. barthii. qRT-PCR analysis of 21-nucletiode phasiRNAs expression levels (a) and lncRNAs (b) from 3 distinct phased loci (PH557, PH612 and PH779) during panicle development in O. barthii (red) and O. glaberrima (blue). Expression values are relative to O. glaberrima stage 4 and the ACTIN gene or miR159 miRNA used as reference is indicated. Panicle stages: Stage 1, Inflorescence meristem stage, after initiation of primary branch (i.e. rachis and primary branch meristems); stage 2, early branching stage (panicle with elongated primary and higher order branch development); stage 3, late branching stage (i.e. panicle with elongated primary and secondary branches with spikelets and beginning of floret differentiation); stage 4, young flowers with differentiated organs. See Additional file 1: Table S5 for primer sequence. (PDF 56 kb) [file 12284_2016_82_MOESM12_ESM.pdf]

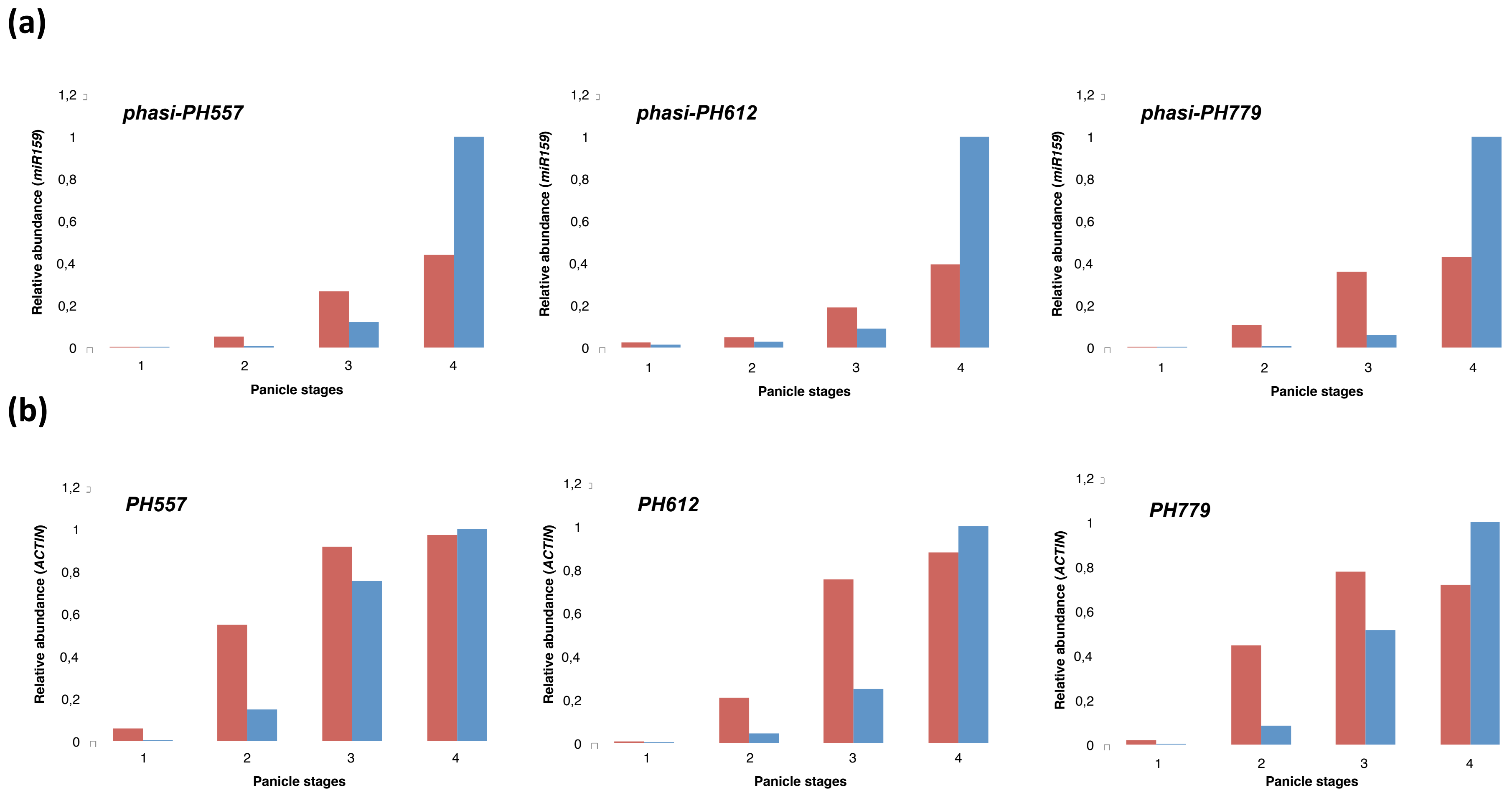

Additional file 12. Expression patterns of phasiRNAs and lncRNA precursors associated with 21-nucleotide phased loci in *O. glaberrima* and *O. barthii*.
